# Effect of Portable Rent Subsidies and Mentorship on Socioeconomic Inclusion for Young People Exiting Homelessness: A Community-Based Pilot Randomized Clinical Trial

**DOI:** 10.1001/jamanetworkopen.2022.38670

**Published:** 2022-10-27

**Authors:** Naomi S. Thulien, Alexandra Amiri, Stephen W. Hwang, Nicole Kozloff, Andrea Wang, Alex Akdikmen, Julia Roglich, Rosane Nisenbaum

**Affiliations:** 1 MAP Centre for Urban Health Solutions, Li Ka Shing Knowledge Institute of St Michael’s Hospital, Unity Health Toronto, Toronto, Ontario, Canada; 2 Dalla Lana School of Public Health, University of Toronto, Toronto, Ontario, Canada; 3 Centre for Critical Qualitative Health Research, University of Toronto, Toronto, Ontario, Canada; 4 Division of General Internal Medicine, Department of Medicine, University of Toronto, Toronto, Ontario, Canada; 5 Department of Psychiatry, University of Toronto, Toronto, Ontario, Canada; 6 Slaight Family Centre for Youth in Transition, Centre for Addiction and Mental Health, Toronto, Ontario, Canada; 7 School of Medicine, Boston University, Boston, Massachusetts; 8 Lawrence S. Bloomberg Faculty of Nursing, University of Toronto, Toronto, Ontario, Canada; 9 Applied Health Research Centre, Li Ka Shing Knowledge Institute of St Michael’s Hospital, Unity Health Toronto, Toronto, Ontario, Canada

## Abstract

**Importance:**

There have been no published randomized clinical trials with a primary outcome of socioeconomic inclusion for young people who have experienced homelessness.

**Objective:**

To explore whether young people exiting homelessness who received rent subsidies and adult mentorship experienced more socioeconomic inclusion relative to young people who received only rent subsidies.

**Design, Setting, and Participants:**

This was a convergent mixed-methods, unblinded, 2-group, parallel randomized clinical trial with 1:1 allocation embedded within a community-based framework in 3 cities in Ontario, Canada. Participants were enrolled between March 1 and September 30, 2019, and were followed up through March 31, 2022.

**Interventions:**

Participants (n = 24) were randomly assigned adult mentors (n = 13) who had been recruited and screened by community partner agencies. All participants received portable rent subsidies (subsidy not tied to a specific location) for 2 years.

**Main Outcomes and Measures:**

Primary quantitative outcomes were self-reported measures of community integration (psychological and physical) and self-esteem—proxy indicators of socioeconomic inclusion. Community integration was measured with the Community Integration Scale, with a score range of 1 to 7 for the physical component and 4 to 20 for the psychological component; higher scores indicate higher integration. Self-esteem was measured with the Rosenberg Self-Esteem Scale, with a score range of 0 to 30; higher scores indicate greater self-esteem. Secondary quantitative outcomes included social connectedness, hopelessness, and academic and vocational participation. All analyses followed the intention-to-treat principle.

**Results:**

A total of 24 youths (12 women [50.0%]; mean [SD] age, 21.8 [2.2] years [range, 18-26 years]; race and ethnicity: 10 White [41.7%], 8 Black [33.3%], 2 Asian [8.3%], 2 Indigenous [8.3%], and 2 different choice [8.3%]) transitioned out of homelessness and into market-rent housing. All youths in the group that received mentorship and in the group that did not receive mentorship had stable or nonsignificant improvements in all study outcomes at the primary end point of 18 months compared with baseline (mean [SD] Community Integration Scale psychological score: mentorship group, 11.3 [2.6] at baseline and 11.2 [3.9] at 18 months; no-mentorship group, 10.8 [4.1] at baseline and 13.2 [2.9] at 18 months; mean [SD] Rosenberg Self-Esteem Scale score: mentorship group, 16.0 [4.6] at baseline and 18.1 [5.2] at 18 months; no-mentorship group, 16.3 [6.1] at baseline and 19.6 [5.7] at 18 months). However, there were no significant differences between the 2 groups in the Community Integration Scale psychological score (adjusted mean difference, −2.0; 95% CI, −5.0 to 1.0; *P* = .18) and Rosenberg Self-Esteem Scale score (adjusted mean difference, −1.4; 95% CI, −5.0 to 2.3; *P* = .44) 18 months after randomization. Ancillary analysis suggested that youths with informal mentors (mentors outside the study) at baseline felt more psychologically integrated at 18 months relative to those with no informal mentors at baseline (adjusted mean difference, 3.6; 95% CI, 0.4-6.8; *P* = .03).

**Conclusions and Relevance:**

In this randomized clinical trial, COVID-19 pandemic–related restrictions made it challenging for mentors and mentees to connect, which may have affected the findings. Steady socioeconomic outcomes—potentially attributable to portable rent subsidies—are noteworthy, given the socioeconomic inequities this population has faced during the COVID-19 pandemic. The possible benefit of informal mentorship warrants further investigation. This small pilot study was designed with the intention of generating data and hypotheses for a full-scale study; findings should be interpreted with caution.

**Trial Registration:**

ClinicalTrials.gov Identifier: NCT03779204

## Key Points

**Question:**

Do young people exiting homelessness with 24 months of portable rent subsidies and adult mentorship experience more socioeconomic inclusion relative to young people who receive only 24 months of portable rent subsidies?

**Findings:**

In this randomized clinical trial of 24 youths who transitioned out of homelessness and into market-rent housing, all socioeconomic inclusion outcomes were stable or showed nonsignificant improvements at 18 months compared with baseline; however, there were no significant improvements within the group that received mentorship relative to the group that did not receive mentorship.

**Meaning:**

The effectiveness of mentorship for young people exiting homelessness—especially under pandemic-related restrictions—is uncertain; stable socioeconomic inclusion outcomes during the COVID-19 pandemic may be attributable to portable rent subsidies.

## Teaser

This randomized clinical trial explores whether young people exiting homelessness who received rent subsidies and adult mentorship experienced more socioeconomic inclusion relative to young people who received only rent subsidies.

## Introduction

The risk factors and challenges associated with homelessness among young people are well documented and include childhood abuse, family dysfunction, involvement in the foster care system, identifying as 2SLGBTQ+ (two-spirit, lesbian, gay, bisexual, transgender, and/or queer), poverty, struggles with mental health, incomplete secondary education, and limited employment opportunities.^1,2,3,4,5,6^ Although youth posthomelessness experiences are still underexplored, the findings are discouraging. Data from longitudinal studies suggest that, despite the attainment of housing stability and irrespective of the type of housing acquired (market rent with limited or no social service supports vs subsidized with social services supports), many youths struggle with a sense of socioeconomic exclusion: poverty-level incomes, meaninglessness, loneliness, “outsiderness,” boredom, hopelessness, and a sense of being “stuck.”^7,8,9,10,11^

In recent years, there have been calls to move beyond the identification of housing stability as a primary outcome for those experiencing homelessness and focus on outcomes related to socioeconomic inclusion.^12,13,14^ From the perspective of the social determinants of health, defining housing stability as the key measure of success in clinical trials risks oversimplifying the issue of homelessness and downplays the importance of focusing on upstream or causal determinants of health, such as socioeconomic position—influenced by factors such as income and employment—and relationships that help connect people to resources (social capital) and facilitate a sense of belonging (social cohesion).^15^ To our knowledge, there have been no published clinical trials with a primary outcome of socioeconomic inclusion for young people who have experienced homelessness.

The overarching objective of this study was to see whether young people who received 24 months of rent subsidies and adult mentorship experienced more socioeconomic inclusion relative to young people who received only 24 months of rent subsidies. We were aware at the outset of this study that, from a socioeconomic inclusion perspective, peer-reviewed literature on youth mentorship signaled promise for informal mentorship (a coachlike nonparental adult naturally existing in a youth’s social network)—especially for youths who had experienced homelessness or were involved in foster care—while peer-reviewed evidence for formal mentorship programs was less compelling.^16^ Thus, our aim was to incorporate features of informal mentorship into a formal mentorship role.

We hypothesized that young people in the intervention (mentorship) group would show a significant increase in self-esteem and community integration (proxy indicators of socioeconomic inclusion) 18 months after randomization relative to the control (no-mentorship) group.^16^ We began this clinical trial in March 2019—1 year before the province of Ontario, Canada, implemented sweeping lockdowns to curb the spread of COVID-19. In this study, we present 18-month quantitative outcome data and discuss the potential effect of the COVID-19 pandemic on our results.

## Methods

### Trial Design

The Transitioning Youth Out of Homelessness study was a mixed-methods, unblinded, 2-group, parallel randomized clinical trial with 1:1 allocation that took place in 3 cities in the province of Ontario, Canada. The study was embedded within a community-based participatory action design, meaning that we worked alongside dedicated community partners from the inception of the study, communicated formally (eg, community advisory board meetings and newsletters) and informally on a regular basis, and remain committed to working with them to incorporate findings from this study into a future project.^17^ All participants provided written informed consent prior to enrollment. Ethical approval for the initial protocol and subsequent amendments were obtained from the St Michael’s Hospital Unity Health Toronto Research Ethics Board. This study followed the Consolidated Standards of Reporting Trials (CONSORT) reporting guidelines. For more detailed information on the study protocol (including amendments), see the trial protocol in Supplement 1.

Participants were enrolled between March 1 and September 30, 2019. Quantitative (self-report questionnaires) and qualitative (semistructured or informal interviews) data collection (Table 1)^18,19,20,21,22,23,24,25,26^ took place every 6 months for 30 months (ie, until 6 months after the 24-month intervention concluded). A self-report baseline demographic questionnaire was administered before randomization and included questions about race and ethnicity (see trial protocol in Supplement 1). We collected this information to assess whether the race and ethnicity of study participants was similar to the population of young people typically served by our community partners and to assess whether there were notable differences between the control and intervention groups. Initially, data collection took place at locations chosen by the participants (eg, local coffee shops or insides their homes); however, starting in March 2020, data collection moved from in person to telephone or video because of the COVID-19 pandemic. Data collection concluded March 31, 2022. The trial was prospectively registered on ClinicalTrials.gov (NCT03779204), and the protocol published.^16^

**Table 1.** Quantitative Instruments: Baseline to 18 Months^a^

Instrument	Psychometric information
Baseline Demographic Questionnaire	This 11-item questionnaire was developed for the study and probed a broad range of demographic information including age, gender, race and ethnicity, immigration status, child welfare involvement, experiences of homelessness, education, and social support.
Beck Hopelessness Scale^18^	This 20-item scale measures motivation, expectations, and feelings about the future (internal consistency α = .93).
	Possible score range: 0-20. Higher scores indicate higher levels of hopelessness (0-3 = none or minimal hopelessness).
Community Integration Scale^19,20^	This 11-item scale measures physical (eg, participation in activities) and psychological (eg, sense of belonging) aspects of community integration (internal consistency α = 0.61 for the 7-item physical component and α = 0.75 for the 4-item psychological component).
	Possible score range: 1-7 (physical component) and 4-20 (psychological component). Higher scores indicate higher integration.
Education, Employment, and Income Questionnaire	This 13-item questionnaire was developed for the study and assessed education (grouped by level), employment (grouped by type), and income (grouped by amount).
Modified Colorado Symptom Index^21^	This 14-item scale measures the presence and frequency of psychiatric symptoms experienced in the past month (internal consistency α = 0.90-0.92).
	Possible score range: 0-56. Higher scores indicate more severe psychiatric symptoms (0-30 = psychiatric disorder unlikely).
Modified Engulfment Scale^22^	This 30-item scale was originally developed to measure the degree to which an individual’s self-concept is defined by their experience of illness (internal consistency α = 0.91). This scale was adapted for this study, with “experience of homelessness” substituted for “illness.”
	Possible score range: 30-150. Higher scores indicate higher sense of engulfment.
Perceived Housing Quality Scale^23,24^	This 7-item scale was shorted from a 10-item scale and measured participant perception of housing choice and quality. The original 10-item scale was used extensively in the Chez Soi/At Home study, but psychometric properties have yet to be reported.
	Possible score range: 7-35. Higher scores indicate higher perception of housing quality.
Rosenberg Self-Esteem Scale^25^	This 10-item scale measures global self-worth (internal consistency α = 0.77-0.88).
	Possible score range: 0-30. Higher scores indicate greater self-esteem (15-25 = self-esteem within normal range).
Social Connectedness Scale–Revised^26^	This 20-item scale measures belongingness—the degree to which an individual feels connected to others (internal consistency α = 0.92).
	Possible score range: 20-120. Higher scores indicate greater sense of connectedness.

^a^ These self-report questionnaires were administered to all study participants at baseline and then every 6 months (Baseline Demographic Questionnaire administered only at baseline).

### Participants

Participants were recruited collaboratively with 3 community partners (The RAFT, Living Rock Ministries, and Covenant House Toronto) and from the cities in which the partners were located—St Catharines (population 133 000), Hamilton (population 552 000), and Toronto (population 2 800 000). Eligible participants were 16 to 26 years of age living in market-rent housing and had experienced homelessness within the past 12 months. Participants had to be fluent in English (primary language of the research team) and able to provide free and informed consent. Participants were excluded if they were in imminent danger of losing their housing (eg, facing jail time or eviction) or were enrolled in a study or program with enhanced economic and social supports.

### Intervention

Young people in both the control and intervention groups (n = 24) received rent subsidies for 24 months. The money was paid directly to their landlords and facilitated by community partners. Rent subsidies were portable because we wanted to provide participants with a sense of ownership and control about where they wanted to live.^27^ Because of differences in the cost of housing, participants in Toronto received CAD$500 per month (US$379), and participants in St Catharines and Hamilton received CAD$400 per month (US$303).

Participants randomly assigned to the intervention group (n = 13) were matched with adult mentors at least 5 years older than the participants. Mentors were recruited and screened by community partners. A total of 14 mentors were included for the 13 participants because 1 mentor was replaced as they could not complete the 2-year commitment. The mean (SD) age of the mentors was 41 (13.7) years, and most (10 of 14 [71.4%]) had no formal mentorship experience. Most mentors (9 of 14 [64.3%]) identified their gender as woman, and most (12 of 14 [85.7%]) identified their race and ethnicity as White (self-identified based on options listed in the baseline questionnaire in the trial protocol in Supplement 1). Almost all mentors (12 of 14 [85.7%]) were employed and represented a wide range of occupations. Covenant House Toronto has an established mentorship program and shared mentorship screening and training resources with the other 2 community partners who did not have an established mentorship program in place. Each community partner designated 1 person to conduct monthly mentor check-ins. Covenant House Toronto hosted quarterly mentor education sessions, and mentors from partner sites were encouraged to attend.

Prior to pandemic-related restrictions, mentors were required to meet in person every month with their mentees and reach out weekly via telephone, e-mail, or text message. After March 2020 (7-10 months after the intervention began, depending on enrollment date), in-person meetings were suspended because of the pandemic. Although there were small windows of opportunity to meet in person between pandemic waves, almost all of the mentor-mentee interactions were through telephone, e-mail, text message, or video chat for the remainder of the 24-month intervention. Our instructions to mentors were relatively simple: act like a “coach” or “cheerleader” (mimicking informal mentorship) and try to connect youths to resources that help facilitate socioeconomic inclusion (eg, visiting a local library or discussing education or employment opportunities).

### Outcomes

We selected community integration (psychological and physical) and self-esteem—proxy indicators of socioeconomic inclusion—as primary outcomes based on our frontline clinical and research experience with this population.^8,10,28,29^ Community integration was measured with the Community Integration Scale,^19,20^ with a score range of 1 to 7 for the physical component and 4 to 20 for the psychological component; higher scores indicate higher integration (Table 1).^18,19,20,21,22,23,24,25,26^ Self-esteem was measured with the Rosenberg Self-Esteem Scale, with a score range of 0 to 30; higher scores indicate greater self-esteem.^25^ Secondary outcomes included social connectedness (measured with the Social Connectedness Scale–Revised, with a score range of 20-120; higher scores indicate a greater sense of connectedness),^26^ hopelessness (measured with the Beck Hopelessness Scale, with a score range of 0-20; higher scores indicate higher levels of hopelessness),^18^ and academic and vocational participation (measured with the Education, Employment, and Income Questionnaire; education was grouped by level, employment was grouped by type, and income was grouped by amount). Exploratory quantitative outcomes included engulfment (measured with the Modified Engulfment Scale, with a score range of 30-150; higher scores indicate a higher sense of engulfment),^22^ psychiatric symptoms (measured with the Modified Colorado Symptom Index, with a score range of 0-56; higher scores indicate more severe psychiatric symptoms),^21^ income (measured with the Education, Employment, and Income Questionnaire), and perceived housing quality (measured with the Perceived Housing Quality Scale, with a score range of 7-35; higher scores indicate higher perception of housing quality).^23,24^ An adverse childhood experiences (ACEs) self-report questionnaire^30^ was added after the study protocol was published because qualitative interviews revealed that most participants were, to varying degrees, still working through the effects of childhood trauma.

### Statistical Analysis

This pilot study was designed with the intention of generating data and hypotheses for a full-scale study. The sample size was pragmatic, chosen in collaboration with community partners based on financial resources available to provide rent supplements for a 2-year period and the feasibility of recruiting mentors willing to make a 2-year commitment; thus, no formal sample size calculation was performed.

After the baseline interview, participants at each of the 3 study cities were randomly assigned using block randomization (blocks of sizes of 2 and 4) to either the intervention group (rent subsidies plus mentoring) or control group (rent subsidies only). In keeping with typical community-based randomized clinical trials with psychosocial interventions, blinding in this study was not feasible, and therefore participants, social service professionals, and mentors knew whether participants were randomly assigned to the intervention or control group.^31^

Study outcomes were analyzed per study protocol.^16^ All analyses followed the intention-to-treat principle. Mean and SD values, median and IQR values, and frequency and proportion summarized baseline characteristics. Analysis of covariance including an indicator of intervention group and the outcome baseline value estimated adjusted mean differences and 95% CIs in continuous outcomes. The Wilcoxon rank sum test was performed for count outcomes. Differences in proportions were estimated and tested using the χ^2^ test or the Fisher exact test. All statistical tests were 2-tailed, and significance was defined as *P* ≤ .05. All analyses were performed using SAS, version 9.4 (SAS Institute Inc).

## Results

A total of 24 youths (12 women [50.0%]; mean [SD] age, 21.8 [2.2] years [range, 18-26 years]; race and ethnicity: 10 White [41.7%], 8 Black [33.3%], 2 Asian [8.3%], 2 Indigenous [8.3%], and 2 different choice [8.3%]) transitioned out of homelessness and into market-rent housing (Table 2).^32^ A total of 13 participants (54.2%) had experiences with child welfare, and just under half (11 [45.8%]) had attempted to exit homelessness 3 or more times. Most participants (19 [79.2%]) had regular contact with an adult relative, and more than one-third (9 [37.5%]) had informal adult mentors. A total of 15 participants (62.5%) were employed, and most (18 [75.0%]) were receiving some form of welfare financial assistance. All participants reported at least 1 ACE; 17 of 23 participants (73.9%) reported 4 to 9 ACEs.

**Table 2.** Baseline Characteristics

Characteristic	Participants, No. (%)		
	Total (N = 24)	Rent subsidies only (n = 11)	Rent subsidies and mentorship (n = 13)
Site			
Toronto	12 (50.0)	5 (45.5)	7 (53.8)
Hamilton	6 (25.0)	3 (27.3)	3 (23.1)
St Catharines	6 (25.0)	3 (27.3)	3 (23.1)
Age, mean (SD), y	21.8 (2.2)	22.2 (2.1)	21.5 (2.3)
First time became homeless			
Age, mean (SD), y	17.8 (3.6)	17.5 (4.3)	18 (3.1)
Gender^a^			
Woman	12 (50.0)	4 (36.4)	8 (61.5)
Man	12 (50.0)	7 (63.6)	5 (38.5)
Race and ethnicity^b^			
Asian	2 (8.3)	1 (9.1)	1 (7.7)
Black	8 (33.3)	3 (27.3)	5 (38.5)
Indigenous	2 (8.3)	2 (18.2)	0
White	10 (41.7)	4 (36.4)	6 (46.2)
Different choice	2 (8.3)	1 (9.1)	1 (7.7)
Born in Canada	20 (83.3)	9 (81.8)	11 (84.6)
Immigration status			
Canadian citizen	21 (87.5)	10 (90.9)	11 (84.6)
Permanent resident	2 (8.3)	1 (9.1)	1 (7.7)
Refugee	1 (4.2)	0	1 (7.7)
Child welfare involvement	13 (54.2)	8 (72.7)	5 (38.5)
No. of attempts to live on own after being homeless			
1-2	13 (54.2)	6 (54.5)	7 (53.8)
≥3	11 (45.8)	5 (45.5)	6 (46.2)
No. of attempts to exit homelessness			
1-2	13 (54.2)	6 (54.5)	7 (53.8)
3-4	4 (16.7)	1 (9.1)	3 (23.1)
≥5	7 (29.2)	4 (36.4)	3 (23.1)
Highest educational level			
Less than high school	8 (33.3)	4 (36.4)	4 (30.8)
Completed high school	8 (33.3)	3 (27.3)	5 (38.5)
Some or completed postsecondary education	8 (33.3)	4 (36.4)	4 (30.8)
Regular contact with adult relative	19 (79.2)	7 (63.6)	12 (92.3)
Informal adult mentor^c^	9 (37.5)	4 (36.4)	5 (38.5)
Employed	15 (62.5)	8 (72.7)	7 (53.8)
Social assistance^d^			
Ontario Works	12 (50.0)	4 (36.4)	8 (61.5)
Ontario Disability Support Program	6 (25.0)	4 (36.4)	2 (15.4)
ACEs, No.^e^			
1-3	6 (26.1)	2 (18.2)	4 (33.3)
4-9	17 (73.9)	9 (81.8)	8 (66.7)

Abbreviation: ACEs, adverse childhood experiences.

^a^ All participants were given the option of choosing “different gender identity”; no one chose this option. We were aware of 1 transgender woman and 1 transgender man in the study.

^b^ Self-identified based on options listed in baseline questionnaire (see trial protocol in Supplement 1). “Different choice” was selected by 2 participants who indicated they were mixed race. We collected this information to assess whether the race and ethnicity of study participants was similar to the population of young people typically served by our community partners and to assess whether there were notable differences between the control and intervention groups. Race and ethnicity were not used to assess study outcomes.

^c^ Not a relative and someone outside the social service sector (ie, not a case worker).

^d^ Ontario Works: approximately CAD$730.00 (US$543.58) per month for basic needs and housing costs. Ontario Disability Support Program: approximately CAD$1700.00 (US$1265.86) per month for basic needs and housing costs for those with a diagnosed disability (eg, physical and/or mental health challenges).^32^

^e^ Adverse childhood experiences questionnaire completed at 24 months; n = 23 (1 participant lost to follow-up at 24 months).

Between March 1 and September 30, 2019, 43 youths identified as potentially eligible were assessed for eligibility, and 24 youths were randomly assigned to the intervention or control group (Figure). Of the 13 youths randomly assigned to receive the intervention, 1 participant chose to no longer receive mentorship prior to their 6-month data collection time point (first time point after baseline), even when a different mentor was offered. Following the intention-to-treat principle, this participant was kept in the intervention group for data analysis. At the 18-month primary end point, there were no participants lost to follow-up, and no harms or unexpected consequences from participating in the study were identified.

**Figure.**
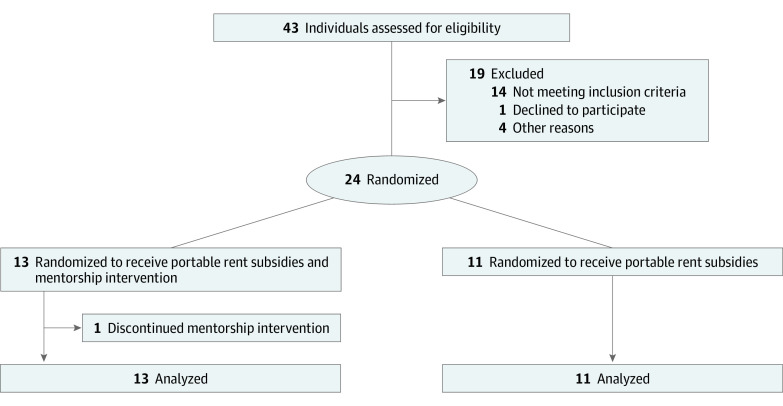
Participant Flow Diagram

### Primary Outcomes

All youths in both groups had stable or nonsignificant improvements at the primary end point of 18 months compared with baseline in the Community Integration Scale psychological score (mean [SD] Community Integration Scale psychological score: mentorship group, 11.3 [2.6] at baseline and 11.2 [3.9] at 18 months; no-mentorship group, 10.8 [4.1] at baseline and 13.2 [2.9] at 18 months; mean [SD] Rosenberg Self-Esteem Scale score: mentorship group, 16.0 [4.6] at baseline and 18.1 [5.2] at 18 months; no-mentorship group, 16.3 [6.1] at baseline and 19.6 [5.7] at 18 months) (Table 3). There were no differences between the groups in the Community Integration Scale psychological score (adjusted mean difference, −2.0; 95% CI, −5.0 to 1.0; *P* = .18) and the Rosenberg Self-Esteem Scale score (adjusted mean difference, −1.4; 95% CI, −5.0 to 2.3; *P* = .44) at the 18-month follow-up. The effect of the COVID-19 pandemic on physical community integration was marked; half of the sample reported no activities at all, and the median number of activities was 1 (IQR, 0-2) in the control group and 0 (IQR, 0-1) in the intervention group. For this reason, a group comparison for this outcome was deemed not applicable at 18 months.

**Table 3.** Primary, Secondary, and Exploratory Outcomes by Group^a^

Outcome	Mean (SD) value				Adjusted mean difference at 18 mo (95% CI)	*P* value
	Rent subsidies and mentorship (n = 13)		Rent subsidies only (n = 11)			
	Baseline	18 mo	Baseline	18 mo		
Community Integration Scale psychological score	11.3 (2.6)	11.2 (3.9)	10.8 (4.1)	13.2 (2.9)	−2.0 (−5.0 to 1.0)	.18
Rosenberg Self-Esteem Scale score	16.0 (4.6)	18.1 (5.2)	16.3 (6.1)	19.6 (5.7)	−1.4 (−5.0 to 2.3)	.44
Social Connectedness Scale score	67.0 (12.9)	74.2 (21.3)	77.0 (15.9)	77.0 (25.8)	7.3 (−9.7 to 24.4)	.38
Beck Hopelessness Scale score	4.5 (4.2)	4.6 (4.8)	7.3 (4.8)	5.8 (5.5)	0.6 (−3.3 to 4.4)	.76
Modified Engulfment Scale score	81.6 (13.6)	70.4 (15.3)	83.8 (28.1)	79.5 (27.0)	−7.2 (−16.4 to 2.0)	.12
Colorado Symptom Index score	40.9 (10.6)	32.9 (10.0)	36.8 (15.4)	34.9 (16.0)	−4.4 (−13.7 to 5.0)	.34
Perceived Housing Quality Scale score	24.6 (4.1)	23.8 (6.1)	23.5 (5.2)	24.6 (6.6)	−1.3 (−6.6 to 4.0)	.62

^a^ Adjusted mean differences at 18 months estimated from analysis of covariance including intervention indicator and baseline outcome value.

### Secondary Outcomes

There were no significant differences between the intervention and control groups in the Social Connectedness Scale score (adjusted mean difference, 7.3; 95% CI, −9.7 to 24.4; *P* = .38) and in the Beck Hopelessness Scale score (adjusted mean difference, 0.6; 95% CI, −3.3 to 4.4; *P* = .76) at the 18-month follow-up (Table 3). With respect to academic and vocational participation, intervention and control groups reported similar changes from baseline to 18 months of follow-up on the Education, Employment, and Income Questionnaire (8 of 13 [61.5%] and 8 of 11 [72.7%], respectively; *P* = .68).

### Exploratory Outcomes

Adjusted mean differences were similar for the intervention and control groups at 18 months for the Modified Engulfment Scale score (−7.2; 95% CI, −16.4 to 2.0; *P* = .12), the Colorado Symptom Index score (−4.4; 95% CI, −13.7 to 5.0; *P* = .34), and the Perceived Housing Quality Scale score (−1.3; 95% CI, −6.6 to 4.0; *P* = .62) (Table 3). With respect to employment income, there was a decrease in both groups reporting any employment income from baseline (intervention group, 7 of 13 [53.8%]; control group, 8 of 11 [72.7%]) to 18 months (intervention group, 5 of 13 [38.5%]; *P* = .32 control group, 5 of 11 [45.5%]; *P* = .26).

### Ancillary Analyses

Informal mentorship at baseline was associated with higher Community Integration Scale psychological scores at 18 months (adjusted mean difference, 3.6; 95% CI, 0.4-6.8; *P* = .03). There were no differences at 18 months between those reporting and those not reporting informal mentorship at baseline for our other outcomes, including our second primary outcome of self-esteem (Rosenberg Self-Esteem Scale score: adjusted mean difference, 1.4; 95% CI, −2.3 to 5.2; *P* = .43). The frequency of mentor-mentee connections decreased after pandemic-related restrictions were implemented (from a mean of 9 connections per month to a mean of 5 connections per month) and by 18 months only 8 of 12 youths (66.7%) were consistently connecting with their mentors. There were no differences at 18 months between those reporting 1 to 3 ACEs and those reporting 4 to 9 ACEs for any study outcomes.

We did not formally measure housing stability; however, we learned through informal conversations at each of the data collection sessions that no one had returned to emergency shelters or lived on the streets since they began receiving rent subsidies. By 18 months, 6 participants (25.0%) reported they had moved back home and were giving the rent subsidies to their family. The annual cost of rent subsidies was CAD$6000 (US$4506) per person for the Toronto participants and CAD$4800 (US$3605) per person for the Hamilton and St Catharines participants.

## Discussion

In this randomized clinical trial of a mentorship intervention for young people exiting homelessness, all of whom received a portable rent supplement, we observed stability or nonsignificant improvements in all study outcomes at the primary end point of 18 months compared with baseline. This finding of stable socioeconomic outcomes over time is noteworthy given the challenges this population faces in general, along with the additional inequities they have faced during the COVID-19 pandemic.^33,34,35,36,37,38,39^ However, there were no significant improvements in proxy indicators of socioeconomic inclusion in the intervention group relative to the control group 18 months after randomization. Thus, we were unable to confirm our study hypothesis.

There are several plausible explanations for our findings. First, none of the participants received the intensity of mentorship that was envisioned in the study design; there were no in-person meetings for more than half of the intervention, and many participants decreased their connections over time. It is difficult to know how much pandemic-related restrictions influenced the quality of these relationships and the desire to maintain connection, but they may have had a substantial effect. Second, our community partners were able to recruit only a small pool of mentors willing to make a 2-year commitment, so young people in the intervention group had limited mentorship choices in terms of factors such as race and ethnicity and gender. For example, most mentors identified as White, while most mentees identified with a different race and ethnicity. Also, most mentors had no formal mentorship experience prior to participating in this study. Although none of the mentees brought up these factors during quantitative or qualitative data sessions, they are important variables to consider and may have affected study outcomes.

Third, during quantitative data collection, many young people informally acknowledged that they enrolled in the study to receive rent subsidies; mentorship was regarded as a potential added bonus. In other words, the question remains as to whether mentorship is effective if young people are not actively seeking this form of support. Fourth, the presence of informal mentors in both the control and the intervention group may have confounded differences between the groups. Finally, it is plausible that the outcome measures we used from baseline to 18 months did not adequately capture the effect of mentorship.

Ancillary analysis suggested that those with informal mentors at baseline experienced more psychological integration at 18 months relative to those with no informal mentors. The possible benefit of informal mentorship—a naturally occurring coachlike relationship—aligns with emerging evidence on the potential of these relationships to improve socioeconomic inclusion outcomes (eg, academic and vocational participation) among youths who are experiencing or at risk of experiencing homelessness.^40,41,42^ A missing ingredient in this study was that the formal mentorships were not naturally occurring relationships; being randomly assigned to mentorship in a study is likely experienced differently than mentorship that emerges organically. The role and characteristics of informal mentorship as an avenue to socioeconomic inclusion align with conceptual insights from our unpublished qualitative data^43^ and warrant further investigation. It is possible that informal mentors are an important mediating factor in socioeconomic inclusion for young people exiting homelessness.

The number of participants with 4 or more ACEs highlights the importance of incorporating a trauma-informed, recovery-oriented approach (eg, fostering a sense of choice and control or mastery) in inclusion-focused interventions with this population.^44^ There is evidence that stable housing may help mitigate poor health outcomes for adolescents with high ACEs.^45^ Portable rent subsidies promote a sense of choice and could play an important role in recovering from the compounding effects of ACEs and homelessness. The upstream drivers of ACEs, such as intergenerational trauma and poverty, must also be addressed, given that ACEs are common risk factors for experiencing homelessness.^46^

Although our intention was to look beyond housing stability, all study participants remained housed at 18 months, despite being in the middle of a global pandemic and associated socioeconomic inequities. The provision of portable rent subsidies also makes sense from a cost-benefit perspective. For example, the annual cost of a shelter stay in Toronto is reported to be CAD$40 000 (US$30 057) per person, and the annual cost of supportive housing (subsidized housing with access to case management) is reported to be CAD$24 000 (US$18 035) per person.^47^ The annual cost of housing Toronto participants was roughly 6.5 times less expensive than the annual cost of staying in a shelter. Moreover, the sense of personal agency and control that comes from having choice is fundamental to recovery-oriented care,^48^ and emerging evidence signals that agency and control could be important mediating factors in outcomes related to mental well-being and housing stability for youths who have experienced homelessness.^27,29,49^ An unexpected finding was the substantial number of young people who chose to move back home and provide the rent subsidies to their parent(s). Given that most participants in this study indicated at baseline that they maintained contact with family, and the fact that many youths experiencing homelessness come from families with limited finances,^3,5,6^ the notion of providing rent subsidies to families warrants further consideration as a strategy to prevent homelessness from (re)occurring.

### Limitations

This study has some limitations. Major changes related to the COVID-19 pandemic began approximately halfway through the intervention for most participants. A similar study conducted under nonpandemic conditions might have obtained different results. As noted previously, none of the participants received the number of mentorship visits envisioned at the outset of this study. The study was conducted with 3 community partners located in 3 urban centers in the province of Ontario, Canada; thus, findings may be different in other contexts. Quantitative data were based on self-reports and may have been affected by social desirability bias. Finally, this pilot study was not adequately powered to detect a significant difference in quantitative outcome measures; findings must be interpreted with caution and are not meant to be generalizable.

## Conclusions

In this randomized clinical trial, COVID-19 pandemic–related restrictions made it challenging for mentors and mentees to connect, which may have affected the findings; however, steady socioeconomic outcomes—potentially attributable to portable rent subsidies—are noteworthy, especially given the socioeconomic inequities this population has faced during the COVID-19 pandemic. The possible benefit of informal mentorship warrants further investigation. Portable rent subsidies and informal mentorship, along with fostering personal agency and control, could be important mediating factors in socioeconomic inclusion and homelessness prevention. Our team aims to test this hypothesis in future work focused on coaching (vs mentoring) and a codesigned (with youths from this study) leadership program for youths exiting homelessness.

## Notes

Supplement 1.
Trial Protocol

Supplement 2.
Data Sharing Statement
