# Error in Table Note

**DOI:** 10.1001/jamanetworkopen.2022.54228

**Published:** 2023-01-13

In the Original Investigation titled “Effect of Portable Rent Subsidies and Mentorship on Socioeconomic Inclusion for Young People Exiting Homelessness: A Community-Based Pilot Randomized Clinical Trial,”^1^ published October 27, 2022, there was an error in footnote *d* of Table 2. CAD$1700.00 (US$1265.86) should be replaced with CAD$1170.00 (US$856.50). This article has been corrected.^1^
