# Supplementary material for: Effect of Portable Rent Subsidies and Mentorship on Socioeconomic Inclusion for Young People Exiting Homelessness: A Community-Based Pilot Randomized Clinical Trial
Source: JAMA Netw Open. 2022 Oct 27;5(10):e2238670. doi: 10.1001/jamanetworkopen.2022.38670 (PMC9614573; doi:10.1001/jamanetworkopen.2022.38670)
Supplement: Supplement 1. — Trial Protocol [file jamanetwopen-e2238670-s001.pdf]

Transitioning Youth Out of Homelessness: A Mixed Methods Community-Based Pilot Randomized Controlled Trial of a Rent Subsidy and Mentoring Intervention in Three Canadian Cities

1. Background and Rationale

Young people comprise almost 20% of the homeless population in Canada (Gaetz, Dej, Richter, & Redman, 2016). It is estimated between 35,000 and 40,000 Canadian youth (ages 13 – 25) are homeless at some point during the year and at least 6,000 on any given night (Gaetz, O'Grady, Kidd, & Schwan, 2016; Gaetz & Redman, 2016).

We know a great deal about the risk factors associated with young people entering and becoming entrenched in street life (e.g., intergenerational poverty, childhood abuse, inadequate education, and limited employment opportunities), but we know much less about how to facilitate and sustain transitions off the streets (Karabanow, 2008; Kidd et al., 2016; Kulik, Gaetz, Crowe, & Ford-Jones, 2011; Mayock, O'Sullivan, & Corr, 2011). In fact, in the peer-reviewed literature, the evidence is scarce to non-existent for rigorous interventions targeting housing outcomes, life trajectories, quality of life, and social integration[1] for young people experiencing homelessness (Altena, Brilleslijiper-Kater, & Wolf, 2010; Coren, Hossain, Pardo, & Bakker, 2016; Hwang & Burns, 2014; Luchenski et al., 2017). Understanding how to create and support successful pathways out of homelessness is crucial, because once youth become entrenched in street life, it becomes much harder for them to exit homelessness and escape a life of poverty (Gaetz, 2014; Karabanow, Carson, & Clement, 2010; Milburn et al., 2009; Public Interest, 2009).

[1] The concept of social integration is complex and often inconsistently defined and poorly measured (Quilgars & Pleace, 2016). For the purpose of this study, we drew from the literature on the social determinants of health and social exclusion, and adopted a holistic definition of social integration, incorporating both the tangible (e.g., access to education and a living wage) and intangible (e.g., sense of connection and belonging) aspects of meaningful and equitable societal participation (Luchenski et al., 2017; Popay et al., 2008; Solar & Irwin, 2010).

Intuitively, it may seem that one important way to improve the life trajectories of young people experiencing homelessness is to provide them with a home. However, from the limited research that has been done in this area, we know that formerly homeless young people continue to experience significant challenges – particularly when it comes to mainstream social integration – even after they are 'successfully' housed (Thulien, Gastaldo, Hwang, & McCay, 2018). Moreover, these challenges seem to persist regardless of the type of housing (e.g., subsidized vs. market rent) provided (Brueckner, Green, & Saggers, 2011; Kidd et al., 2016; Kozloff et al., 2016).

Evidence from one of the most rigorous longitudinal studies with formerly homeless youth (ages 16 – 25) to date highlights that the procurement of a home does not necessarily translate into a sense of belonging or connection to mainstream society (Kidd et al., 2016). This mixed methods study of 51 formerly homeless young people living in two major urban centers in Canada showed that, despite living in stable or semi-stable accommodations (53% lived in subsidized housing), participants continued to face substantial challenges such as poverty-level incomes and limited mainstream social networks which, over the course of one year, contributed to a significant decline in hope, no gains in community integration, and a sense of being 'stuck' (Frederick, Chwalek, Hughes, Karabanow, & Kidd, 2014; Karabanow, Kidd, Frederick, & Hughes, 2016; Kidd et al.). Moreover, community integration challenges were significantly worse for participants living in independent (market rent) housing.

A sub-group analysis of 156 young people (ages 18 – 24) with mental health challenges who participated in a 24-month randomized controlled trial (RCT) of 'Housing First' (access to subsidized housing and comprehensive social service supports [e.g., treatment for mental health challenges] at home or in the community) in five Canadian cities – the largest RCT of Housing First to date – indicate similar findings of ongoing hardship despite achieving housing stability (Kozloff et al., 2016). While the young people who received the Housing First intervention achieved significantly better housing stability compared to the treatment as usual group, they did not experience any additional improvements to other outcomes such as employment, generic quality of life, and community integration relative to treatment as usual (Kozloff et al.). Notably, the same community integration scale (Stergiopoulos et al., 2014)

was used in this RCT and the aforementioned Kidd et al. study, and measures both physical integration (e.g., attending a movie or community event) and psychological integration (e.g., interactions with others and feeling like one belongs).

Findings from a ten-month ethnographic study with nine formerly homeless young people (ages 18 – 24) living in Canada's largest city also support the idea that transitioning young people out of homelessness and helping them integrate into the mainstream likely requires much more than simply providing them with a home (Thulien et al., 2018). This study is believed to be the first ethnographic study to exclusively focus on the integration experiences of formerly homeless young people living in market rent housing and showed that, despite the appearance of housing stability, the participants were living a precarious existence, attributed in part to the chronic stress and exhaustion of living in poverty and to their limited knowledge about how to move forward in life (Thulien et al.). In addition, the authors note that participants underutilized transition-related social supports (e.g., food banks and employment counseling) because these supports tended to be deficit-focused (e.g., focused on what youth did not have, not on what they had achieved) and located in areas (e.g., homeless shelters) that reminded them of their old identities as homeless youth.

As previously mentioned, little evidence exists for effective interventions that target social integration for young people who have experienced homelessness. This includes evidence on the impact of mentorship. In fact, for formal mentorship programs in general, meta-analyses have only found small overall positive effect sizes (i.e., the impact of the average mentoring program in improving youth outcomes) on the psychological, emotional, behavioral, and educational functioning of participating young people (Thompson, Greeson, & Brunsink, 2016; Van Dam et al., 2018). However, there is some emerging evidence on the benefits of 'natural mentors' – generally defined as an important, encouraging, non-parental adult that exists in a youth's social network – that may be transferrable to youth who have experienced homelessness.

A systematic review of natural mentoring for youth (ages 13 – 25) transitioning out of foster care showed that the young people benefited from a supportive adult not "tasked with

enforcing daily rules and addressing misbehavior" (p. 48) and that this intervention resulted in improved behavioral, psychosocial, and academic outcomes (Thompson et al.). The authors stress the importance of cultivating *inter*dependence (as opposed to independence) for young people leaving foster care and suggest that, while traditional natural mentoring relationships tend to emerge organically, they can be facilitated and supported programmatically as well (see https://vimeo.com/115837436).

A more recent meta-analysis of natural mentoring in youth (ages 13 – 24) also supports the notion that the presence of a natural mentor can positively impact young people (Van Dam et al., 2018). This meta-analysis included all young people (not just 'at-risk' youth) and found that, similar to the aforementioned systematic review, positive youth outcomes were particularly significant in the domains of social and emotional development, and academic and vocational functioning (Van Dam et al.). Moreover, the authors found that risk status (e.g., young people who were homeless or living in foster care) did *not* moderate these positive outcomes.

While almost all of the reviewed studies of at-risk youth in the meta-analysis and the systematic review were limited by their cross-sectional design, the results do hold promise for mentoring interventions that incorporate the positive characteristics of natural mentors (i.e., more of a friendship-like, 'coach', or 'cheerleader' role) for young people who have experienced homelessness. These findings are supported by a small (n = 23) qualitative study of natural mentoring relationships among homeless youth (ages 14 – 21) that suggests "natural mentors could feasibly serve as a bridge in a coordinated effort to assist youth out of homelessness" (Dang & Miller, 2013, p. 7).

From the limited research that has been done with young people transitioning away from homelessness, the emerging evidence seems to indicate that, while structural supports such as subsidized housing and social service providers are important, these things alone are insufficient to help young people integrate into mainstream society. As it currently stands, it appears as if the burden for achieving meaningful social integration is on the formerly homeless young people, who continue to be marginalized despite achieving stable or semi-

stable housing (Quilgars & Pleace, 2016; Thulien et al., 2018). Connecting these young people with an adult who exhibits the relationship-based components of natural mentoring that young people value most (e.g., genuine interest in their well-being and belief in their ability to succeed, a non-judgmental attitude and a willingness to listen, the provision of advice, guidance, affirmation and encouragement) (Dang & Miller, 2013; Thompson et al., 2016; Van Dam et al., 2018) may be key to helping them move forward and integrate into the mainstream.

This intervention will provide 24 young people (ages 18 – 26) who have transitioned out of homelessness and into market rent housing within the past year with rent subsidies for 24 months. Half of the young people will also receive mentorship. We chose to focus on young people living in market rent housing because, due to limited subsidized housing options, this is the reality for most young people exiting homelessness.

Initially, we proposed to our community partners a study design where only half the young people would receive rent subsidies, with the other half receiving 'treatment as usual'; however, we abandoned this idea after our community partners challenged the ethics of not providing or delaying rent subsidies for young people living a precarious existence and desperate for immediate, tangible support to help them remain in market rent housing. Consequently, we adopted the stance that, given housing is a basic human right[2], we would offer rent subsidies to *all* of the study participants. While this does move us away from the 'gold standard' in terms of measuring the impact of rent subsidies on social integration, we believe the mixed methods longitudinal design will still yield important insights in this regard. Moreover, this design adaptation reflects our deep commitment to engage in community-based participatory action research (CBPAR) – a methodology that challenges traditional epistemological assumptions of what constitutes 'good' evidence, demands researcher humility, stresses genuine and equitable academic-community partnerships, and

[2] "Adequate housing is essential to one's sense of dignity, safety, inclusion and ability to contribute to the fabric of our neighbourhoods and societies…without appropriate housing it is often not possible to get and keep employment, to recover from mental illness or other disabilities, to integrate into the community, to escape physical or emotional violence or to keep custody of children" (Ontario Human Rights Commission, n.d.).

facilitates the undertaking of research in a way that the *community* feels is most beneficial to their members (Goodkind et al., 2017; Wallerstein & Duran, 2006; Wallerstein, Duran, Oetzel, & Minkler, 2018).

Half of the young people will be randomized to receive regular mentorship from an adult mentor, tasked with helping their mentee bridge the gap between homelessness and mainstream living. While some of these mentors will not be 'natural' in the sense that these may not be pre-existing, organically-formed relationships (see 3a. Mentorship), the mentors will incorporate the key relationship-based components of natural mentoring mentioned previously, with a strong emphasis on a strengths-based approach (i.e., focus on the young person's strengths as opposed to their limitations) and the connection of participants to larger social networks (including education and employment).

Findings from this longitudinal pilot randomized controlled trial will help address the gap in our knowledge about the impact of financial support and mentorship on meaningful social integration for young people who have experienced homelessness and are living in market rent housing.

2. Study Aim and Objectives

The overarching aim of this mixed methods study is to assess whether and how rent subsidies and mentorship influence social integration outcomes for formerly homeless young people living in market rent housing in three urban settings.

Specifically, the objectives of this study are to:

1. Determine whether rent subsidies plus mentorship results in better social integration outcomes than only receiving rent subsidies with respect to: a) community integration (psychological and physical); and b) self-esteem at our primary endpoint of 18 months.
2. Determine whether rent subsidies plus mentorship results in better social integration outcomes than only receiving rent subsidies with respect to: a) social connectedness; b) hope; and c) sustained academic and vocational participation at our secondary endpoint of 18 months.

3. Explore whether rent subsidies plus mentorship results in better social integration outcomes than only receiving rent subsidies with respect to: a) income; b) perceived housing quality; c) psychiatric symptoms; and d) sense of engulfment at our exploratory endpoint of 18 months.
4. Integrate qualitative data to facilitate a fuller understanding of the quantitative data and deepen our understanding of what the study participants (young people and mentors) found most beneficial about the intervention and how it could be improved.

3. Study Design

This study will employ a convergent mixed methods design (i.e., quantitative and qualitative data are collected concurrently, and the findings combined) embedded within a RCT and a CBPAR framework (Creswell, 2014; Creswell & Plano Clark, 2018). We believe a mixed methods RCT is appropriate given the complex explanatory pathways (i.e., social and behavioral processes that may act independently and interdependently) of this intervention (Lewin, Glenton, & Oxman, 2009). In addition, the qualitative data will provide insights on contextual factors that may impact the external validity of our findings (Goodkind et al., 2017). Most importantly, this design provides a crucial (and underutilized) youth-informed perspective on social integration.

The study will be conducted in three Canadian cities: Toronto, Ontario (pop. 2.8 million); Hamilton, Ontario (pop. 552,000); and St. Catharines, Ontario (pop. 133,000). The design and implementation of this study is very much collaborative effort between our research team and the following community partners: a) Covenant House Toronto; b) Social Planning and Research Counsel of Hamilton; and c) The RAFT (St. Catharines).

All of the study participants (n = 24) will receive rent subsidies (ranging from \$400 – \$500/month)[3] for 24 months. This study includes funding for the rent subsidies and will be paid directly to the landlords by our community partners. St. Michael's Hospital will establish a service provider agreement with each of our community partners for this purpose.

[3] Given the higher cost of rent in Toronto, youth living in Toronto will receive \$500/month, while youth living in Hamilton and St. Catharines will receive \$400/month.

3a. Mentorship

Participants in the intervention group (*n* = 12) will be matched with an adult mentor recruited by one of our community partners. Each of our community partners expressed a strong desire to take the lead in the screening and recruitment of mentors as they feel they are in the best position to work with the study participants to ensure the best mentor 'fit'. Drawing on the expertise of our community partners and sharing decision-making power is aligned with CBPAR principles and highlights our commitment to collaborative, equitable partnerships in all phases of the research process (Israel et al., 2018). Moreover, working with established community resources makes practical sense; not only will this facilitate co-learning and capacity building between the research team and our community partners (Israel et al.), but delivering the mentorship intervention under 'real world' conditions will provide important insights into scalability and sustainability (Wallerstein & Duran, 2010).

Given our study aim of understanding the impact of 'natural' mentorship on the outcome variables, each youth in the intervention group will be asked whether they have a natural mentor in their life. If so, this person will be approached by our community partners about being a mentor in the study. If not, our community partners will approach appropriate volunteers within each organization (Covenant House Toronto has a formal mentorship program, while the other two sites have a core group of committed volunteers) and asses their interest in becoming a mentor. Given the small number of mentors needed for this pilot study (six in Toronto; three in Hamilton; three in St. Catharines), our community partners are confident that they will be able to recruit suitable mentors.

To build capacity between community partners, Covenant House Toronto will share their comprehensive Mentor Program Guidelines and Mentor Orientation Handbook (attached to this study protocol), which will act as a guide for all sites. These booklets cover information ranging from ideal mentor characteristics to mentor code of conduct. Each of our partners will designate one person currently serving in a leadership role within the organization to conduct one-on-one interviews with potential mentors and make the final decision (in conjunction with study participants) about mentor-mentee matches. In Hamilton and St.

Catharines, this process will be undertaken by the executive director of each organization and, in Toronto, it will be done by the co-ordinator of their mentorship program.

As mentioned previously, our community partners are firm in their desire to control the mentorship screening and recruitment process, and will do so in a way that works best for each organization. That being said, all three organizations have agreed to the following preliminary screening process prior to meeting potential mentors:

- The mentor must show original documentation of passing a Vulnerable Sector police check within the past three months
- The mentor should ideally be at least five years older than the mentee
- The mentor must provide three references; one must be from a current employer

The mentors will be encouraged to incorporate the key relationship-based components of natural mentors previously described (e.g., a ‘coach’ or ‘cheerleader’ role) to assist with mainstream integration. To facilitate more of an organic, natural mentor-mentee relationship, the mentors will have more flexibility than a typical formal mentorship program in the types of activities they pursue with their mentees. For example, they will not be mandated to attend shelter-based social events. Instead, mentors will be encouraged to initiate activities that direct their mentees *away* from the shelter system (and their old identities as homeless youth) and toward the mainstream (e.g., meeting for coffee at a local university campus, touring a local library, or visiting the mentor’s place of employment during business hours). All of the mentors will meet monthly with their mentees for two years. In addition, the mentor will be encouraged to touch base with their mentee via phone or text message every week. If a mentor is unable to continue their role and there are at least six months left in the study, the study participant will be matched with a new mentor.

3b. Outreach Worker

Our community partners will match all participants with an outreach worker (already employed by each agency) who will communicate regularly with the research team, help ensure the rent subsidies are being distributed appropriately, maintain an ongoing relationship with the study participants, and monitor for ‘red flags’ in participants matched in

mentor-mentee relationships (e.g., mentee reluctant to meet with their mentor). Matching all of the study participants with a worker will also help ensure that everyone is receiving a fairly equal level of social support from our community partners, making it easier for the research team to discern whether the outcomes of interest are more likely attributable to mentorship rather than to varied levels of agency-based support. Moreover, a review of services and interventions designed to reduce "problem behaviors" (p. 733) (e.g., substance use and risky sexual practices) among street-involved and homeless young people (ages 12 – 24) found that researchers who had strong relationships with outreach workers and the community had more effective interventions and lower attrition rates than those who did not (Slesnick, Dashora, Letcher, Erdem, & Serovich, 2009).

3c. Methodology

As previously noted, this mixed methods RCT is embedded within a CBPAR framework. With the goal of reducing health inequities through knowledge and action, CBPAR can be a powerful tool for those working with marginalized populations (Chenail, St. George, Wulff, & Cooper, 2012; Rutman, Hubberstey, Barlow, & Brown, 2005; Wallerstein & Duran, 2006; Wallerstein et al., 2018). The ontological and epistemological assumptions underpinning CBPAR methodology are closely aligned with Critical Social Theory – that is, the belief that social conditions (e.g., socioeconomic contexts) perpetuate societal power imbalances and shape our version of 'truth' (Denzin & Lincoln, 2011; Prasad, 2005). For example, some may underestimate the social integration challenges faced by formerly homeless young people because they believe that everyone is afforded the same life chances. Thus, researchers operating within this paradigm have a goal of exposing and critiquing the inequitable (and often invisible) conditions that make it challenging for the marginalized to move forward (Strega, 2005).

We will draw on the following key principles of CBPAR as we generate and analyse data (Chenail et al., 2012; Rutman et al., 2005; Wallerstein & Duran, 2006; Wallerstein et al., 2018):

- Research participants are viewed as experts in their own lives

- Concerted effort to reduce/eliminate power imbalances between the researchers and the community
- Equal value placed on academic (researcher) knowledge and experiential (community agency/youth) knowledge
- Commitment to producing practical, ‘actionable’ data to build community capacity and improve/transform the lives of the research participants
- Duty to remain invested with the community beyond the life of the research project

3d. Participant Eligibility and Recruitment

Twenty-four young people ages 18 – 26 who have left homelessness within the past year and are living in market rent housing will be collaboratively recruited by our research team and our community partners Covenant House Toronto, Social Planning and Research Counsel of Hamilton, and The RAFT (St. Catharines).

In addition to the above age and housing **inclusion** criteria, study participants must:

- Be able to provide free and informed consent
- Be fluent in English
- Plan on staying in or nearby the community in which they were recruited (Toronto, Hamilton, or St. Catharines) for the duration of the 24-month study
- Be willing to be matched with an adult mentor who has been screened and chosen by one of our three community partners (Covenant House Toronto, Social Planning and Research Counsel of Hamilton, or The RAFT)

Young people will be **excluded** from the study if they are:

- In imminent danger of losing their housing (e.g., facing jail time or impending eviction)
- Enrolled in another study with enhanced financial and social supports
- Receiving welfare subsidies[4]

[4] As it currently stands, youth on Ontario Works or the Ontario Disability Support Program would be penalized for receiving rent subsidies as this money would count as income. Participants would only be allowed to keep the first $200 each month, with the rest being deducted at a rate of 50 cents for every dollar provided. This would also put them at risk of losing their welfare subsidies (because they

3e. Consent Process

Free and informed consent will be obtained verbally and in writing from all study participants. A concerted effort has been made to ensure the consent form is in plain language. Highlighted throughout the document is the fact that informed consent is an ongoing process and can be negotiated at any time.

All of the study participants will be screened for eligibility (see criteria above) and recommended for the study by one of our community partners (Covenant House Toronto, Social Planning and Research Counsel of Hamilton, and The RAFT). A member of the research team will call each recruited youth on the telephone and arrange to meet them at a location most convenient for the youth. Potential participants will be given a copy of the participant information and consent form to read. This document will also be reviewed verbally to ensure that those who have low literacy levels have been given the information required to give informed consent. During this process, the research team member will assess the capacity of the potential participant to provide free and informed consent. If it is unclear whether a youth is able to provide consent, the study co-investigator (Dr. Naomi Thulien) will be contacted immediately. Dr. Thulien will use her knowledge, skill, and judgement as a nurse practitioner who works exclusively with street-involved and homeless young people to make a judgment regarding consent. If it is determined that a youth is not able to consent, they will be informed of this during the initial meeting (or at a later date if Dr. Thulien is not immediately accessible) and they will be excluded from the study. The appropriate community partner will be notified as well.

3f. Allocation Procedure

If the participant meets the eligibility criteria, informed consent will be obtained, and the participant will be enrolled in the study. During this initial meeting, enrolled participants will participate in a baseline interview. There will be no unmasking of assignment prior to randomization. Following the baseline interview, participants at each of the three study sites (Toronto, Hamilton, and St. Catharines) will be randomized using covariate adaptive

earn 'too much') and the enhanced supports (e.g., special diet allowance) that go along with these subsidies.

randomization to either the intervention (rent subsidies plus mentoring) or control (rent subsidies only) group via computer using the software program Research Randomizer (Urbaniak & Plous, 2013). Covariate adaptive randomization is ideal for this small trial because it will help minimize an imbalance of our pre-identified covariates (gender and race/ethnicity) during rolling enrollment (Suresh, 2011). We will aim to have a roughly even balance of gender[5] and ethno-racial representation at each site. Participants will be informed immediately if they have been allocated to the intervention or control group (Figure 1). In keeping with typical community-based RCTs with psychosocial interventions, 'blinding' in this study would not be pragmatic (e.g., social service providers and mentors will know if participants are in the 'treatment' group) after the baseline interviews and random assignments have been conducted (Solomon, Cavanaugh, & Draine, 2009).

[5] Participants who do not identity as male or female will be randomized according to sex.

Figure 1.

CONSORT (**CON**solidation of the **S**tandards **O**f **R**eporting **T**rials) Diagram of Ideal Flow of Participants Through the Study*

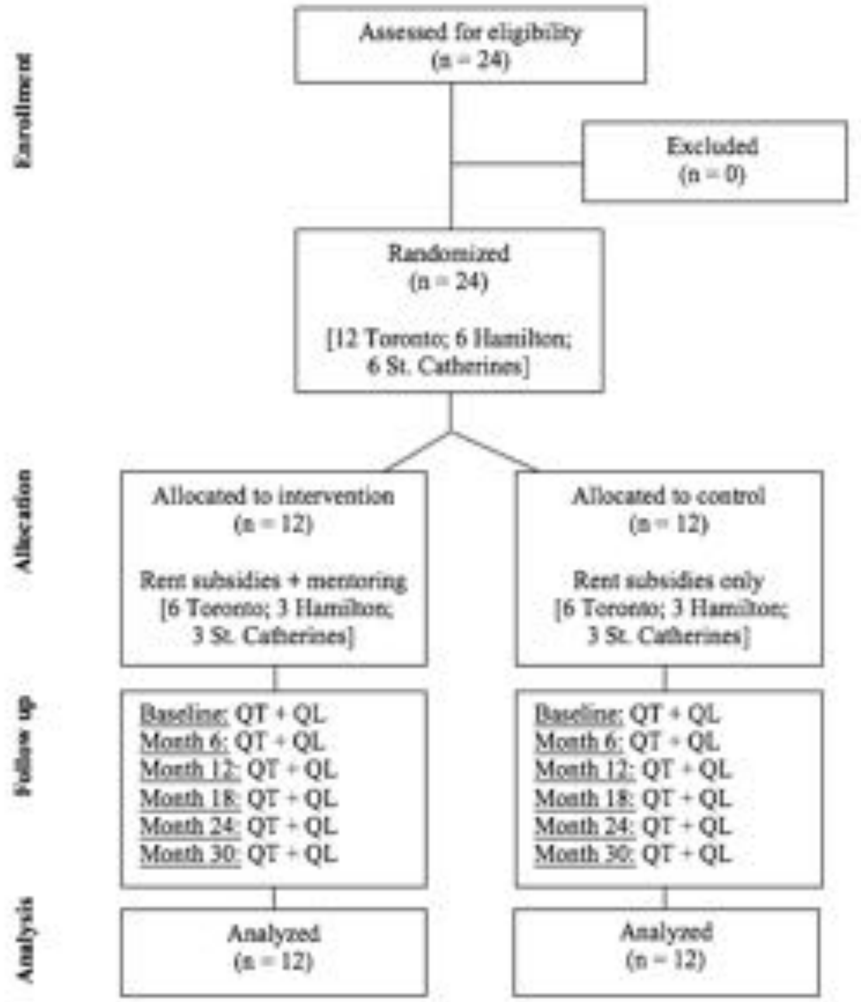

***QT** = quantitative measures (all participants). These will consist of six standardized measures to assess: community integration, social connectedness, engulfment, hope, self-esteem, and psychiatric symptoms. As well, participants will complete two brief questionnaires pertaining to: 1) education (includes skills training), employment, and income; and 2) perceived housing quality. **QL** = qualitative measures (12 participants). These will consist of one-on-one semi-structured interviews with the same 12 participants (six from each arm). The interview questions will explore issues related to feasibility and acceptability, and provide context to the quantitative responses.

4. Data Generation

Undertaking a mixed methods study where data is truly 'mixed' at the level of collection (not just at analysis) is challenging because it requires a solid understanding of the data generation requirements of each research paradigm, and the interviews can take longer to conduct than those focusing on qualitative or quantitative methods alone (Farquhar, Ewing, & Booth, 2011). To minimize respondent burden, we have given careful consideration to the type of quantitative instruments chosen (e.g., number and length of time to complete) and will ensure that appropriate components of the qualitative interviews are prioritized (e.g., follow-up on changes in instrument scores) at each data generation session (Farquhar et al.). Additionally, all of the mixed method interviews will be conducted by the co-investigator, Dr. Naomi Thulien, who has expertise in conducting mixed method interviews with young people who have experienced homelessness.

4a. Study Outcomes

As mentioned previously, the mixed methods design of this pilot RCT reflects our desire to capture the complex independent and interdependent explanatory pathways of the intervention. This is especially crucial during this pilot stage, where we will be paying particular attention to feasibility, context, and unexpected mechanisms that produce change – factors that will influence study outcomes and provide important information regarding scalability and sustainability (Craig et al., 2008; Moore et al., 2015). To fully apprehend these complex explanatory pathways, "represent the best use of the data," and "provide an adequate assessment of the success or otherwise of an intervention that has effects across a range of domains" (Craig et al., p.3), we have aligned our key outcome variables (Table 1) with the Medical Research Council guidance on evaluating complex interventions and identified more than one primary outcome measure.

Table 1. Key Outcome Variables

**Variables**	**Instruments***
Community integration (psychological and physical)	Community Integration Scale

Social connectedness<br>Engulfment	Social Connectedness Scale – Revised<br>Modified Engulfment Scale
Self-esteem<br><br>Hope<br>Psychiatric symptoms	Rosenberg Self-Esteem Scale<br><br>Beck Hopelessness Scale<br>Modified Colorado Symptom Index
Enrollment in education (includes skills training)<br>Employment	Composite checklist
Income	Composite checklist
Perceived housing quality	Perceived Housing Quality Scale
Participant perspectives of barriers and facilitators	Individual semi-structured interviews (youth) and focus groups (mentors)

*See Table 2 for references and psychometric properties. All will be administered every six months for 30 months.

The **primary outcome measures** for this study are: community integration (psychological and physical) and self-esteem. **Secondary outcomes** include: social connectedness, hope, and academic and vocational participation. **Exploratory outcomes** include: engulfment, psychiatric symptoms, income, perceived housing quality, and participant perspectives of intervention barriers and facilitators.

4b. Study Hypothesis

We hypothesize that, for the **primary outcome** measures of community integration and self-esteem:

1. We will observe better mean scores (community integration and self-esteem) in the participants who receive rent subsidies plus mentorship (intervention group) compared to the participants who receive rent subsidies only (control group) by our **primary endpoint of 18 months** of study participation.

We hypothesise that, for the **secondary outcome** measures of social connectedness, hope, and academic and vocational participation:

1. We will observe better mean scores (social connectedness and hope) in the intervention group relative to participants in the control group by our **secondary endpoint of 18 months** of study participation.
2. Participants in the intervention group will be more likely than the control group to demonstrate sustained engagement in academic and vocational activities (education, employment, and/or skills training) by our **secondary endpoint of 18 months** of study participation.

4c. Quantitative Measures

Quantitative data (Appendix A – Quantitative Data Collection) will be collected at **six points** in time over the course of 30 months: baseline, month six, month 12, month 18, month 24, and month 30. **Eight instruments** (Table 2) will be employed to assess the outcome variables. We have purposely chosen instruments utilized in previous research with young people who have experienced homelessness (e.g., Kidd et al., 2016; Kozloff et al., 2016; McCay et al., 2015) so that meaningful comparisons can be made across studies (Moore et al., 2015) in this nascent area of research. One instrument we do not believe has been used with this population is the Modified Engulfment Scale (McCay & Seeman, 1998). We have included engulfment as an exploratory outcome given the emerging qualitative evidence on the crucial role of identity (self-concept) in a young person's transition away from homelessness (Brueckner et al., 2011; Karabanow et al., 2016; Thulien et al., 2018).

Table 2. Quantitative Instruments

**Instrument**	**Psychometric Information**
Beck Hopelessness Scale (Beck, Weissman, Lester, & Trexler, 1974).	This 20-item scale measures motivation, expectations, and feelings about the future (internal consistency $\alpha = .93$).
Community Integration Scale (Stergiopoulos et al., 2014).	This 11-item scale measures behavioral (e.g., participation in activities) and psychological (e.g., sense of belonging) aspects of community integration. This scale was used extensively in the Chez Soi/At Home study, but psychometric properties have yet to be reported.

Education, Employment, and Income Questionnaire	This 13-item questionnaire assesses education, employment, and income. We developed this questionnaire for the study.
Modified Colorado Symptom Index (Ciarolo et al., 1981).	This 14-item scale measures the presence and frequency of psychiatric symptoms experienced in the past month. (internal consistency $\alpha = .90 – .92$).
Modified Engulfment Scale (McCay & Seeman, 1998).	This 30-item scale measures the degree to which an individual's self-concept is defined by their experience of homelessness (internal consistency $\alpha = .91$). We have adapted the scale for this study, substituting "experience of homelessness" for "illness".
Perceived Housing Quality (Toro et al., 1997).	This seven-item scale measures participant perception of housing choice and quality. This scale was used extensively in the Chez Soi/At Home study, but psychometric properties have yet to be reported. We have shortened it from 10 items (Chez Soi/At Home) to seven relevant items.
Rosenberg Self-Esteem Scale (Rosenberg, 1965).	This 10-item scale measures global self-worth (internal consistency $\alpha = .77 – .88$).
Social Connectedness Scale – Revised (Lee & Robbins, 1995).	This 20-item scale measures belongingness – the degree to which an individual feels connected to others (internal consistency $\alpha = .92$).

4d. Qualitative Measures

Qualitative measures (see Appendix B – Qualitative Data Generation) are an important feature of this study and will consist of: **1) semi-structured individual interviews** (study participants) and **2) focus groups** (mentors).

At baseline, twelve participants (six from each arm of the study) will be invited to participate in **six semi-structured individual interviews**, which will take place at the same time as the quantitative data collection: **baseline, month six, month 12, month 18, month 24, and**

month 30. Participants will be purposively selected with a goal of having input from each of the three communities and a fairly equal gender and ethno-racial representation.

All of the mentors (*n* = 12) will be invited to participate in **two focus groups**, which will take place at **month 12 and month 24**.

The questions posed during the semi-structured interviews and focus groups will be guided by the study objectives, but will be conversational and exploratory in nature with particular attention to understanding *how* mentoring and/or rent subsidies influence social integration outcomes for formerly homeless young people living in market rent housing. Given the emergent nature of qualitative inquiry (Denzin & Lincoln, 2011; Eakin & Mykhalovskiy, 2003), we expect the interview and focus group questions to evolve over time as key preliminary themes begin to surface. It is anticipated that the individual interviews (including quantitative data collection, which will consistently take place first) will last approximately 60 – 75 minutes, and the focus groups approximately 60 – 90 minutes. The individual interviews and the focus groups will be conducted by Dr. Naomi Thulien at locations most convenient for those participating. To get a better sense of each young person's living situation and to minimize researcher – participant power imbalance (Israel et al., 2018), Dr. Thulien will suggest that the individual interviews take place in or nearby the young people's homes. The individual interviews and focus groups will be audio recorded and transcribed verbatim.

4e. Honoraria

All of the study participants will be paid an honorarium of $20 at each of the six quantitative data collection points. Those participating in semi-structured interviews will be paid an additional $30 at each interview. This amount was based on the co-investigator's previous experience with this population and after consulting with our community partners.

5. Data Analysis

One major critique of mixed methods RCTs is that, typically, there is no true integration (i.e., 'mixing') of quantitative and qualitative findings at the level of analysis or interpretation

(Lewin, Glenton, & Oxman, 2009). Moreover, it is often unclear whether or how the quantitative and qualitative researchers have worked together to maximize the potential synergies between these different approaches (Lewin et al.). With this in mind, our study team, consisting of researchers with quantitative and qualitative expertise, worked together to develop this study protocol and anticipate meeting quarterly to discuss the emerging analysis and to explore (and follow up on) similarities or discrepancies between the quantitative and qualitative data.

5a. Quantitative Data

All analyses will be performed using the intention-to-treat principle; that is, all participants will be included and analyzed in the groups they were originally randomized. Baseline characteristics of the intervention and control groups will be summarized using descriptive statistics (i.e., mean, standard deviation, median and interquartile range for continuous variables, and frequencies and proportions for categorical variables). We will also calculate descriptive statistics for outcomes at each study time point, and will explore differences in trajectories from baseline to 30 months follow-up between intervention and control groups using scatterplots and box-plots. Differences with 95% confidence intervals in continuous outcomes at 18 months (psychological community integration, self-esteem, social connectedness, hope, perceived housing quality, psychiatric symptoms, and sense of engulfment) between participants who received rent subsidies plus mentorship and participants who only received rent subsidies will be estimated using Analysis of Covariance (i.e., linear regression models), including an indicator of intervention group and the baseline value of the outcome. We will perform regression diagnostics and will repeat analyses using the non-parametric Wilcoxon rank-sum test if there are extreme outliers or influential observations. Groups will be compared with respect to count outcomes at 18 months (physical community integration) using graphical tools and the non-parametric Wilcoxon rank-sum test. For binary outcomes at 18 months (sustained academic and vocational participation, and income above low income cut-off[6]), differences in proportions with 95% confidence intervals will be estimated and tested using the chi-square or Fisher's exact test.

[6] Based on family and community size.

Given the small sample size of this pilot randomized trial, all results will be interpreted with caution and with the intention of generating data and hypotheses for conducting a larger trial.

All efforts will be made to reduce participants' attrition and drop-out. As mentioned previously, we believe our strong relationship with the outreach workers and community agencies will help minimize loss to follow-up (Slesnick et al., 2009). In addition, we have made it very clear in the participant information and consent form that participants in the rent subsidies plus mentorship arm may continue in the study (receiving only rent subsidies) if they are unable to continue in a mentor-mentee relationship.

5b. Qualitative Data

In keeping with the emergent, iterative nature of research using a qualitative design (Denzin & Lincoln, 2011; Eakin & Mykhalovskiy, 2003), data analysis and interpretation will begin immediately after the first qualitative data generation session (at baseline). The semi-structured individual interviews and focus groups will be audio recorded and transcribed verbatim. In order to conduct a more nuanced analysis of the data, the transcriptionist will be instructed to note short responses, uncooperative tones, and literal silence (Eakin & Mykhalovskiy; Kawabata & Gastaldo, 2015). Prior to each subsequent qualitative data generation session, members of the research team will conduct a preliminary data analysis, reading the interview transcripts multiple times, separating the data into coded segments, making analytic memos beside sections of the transcripts, identifying emerging themes (and comparing/contrasting these between respondents), and compiling new questions (Creswell, 2014; Denzin & Lincoln). Those participating in the individual interviews and the focus groups will be asked for their perspectives on the emerging interpretations at each visit and these perspectives will play a key role in helping shape the data analysis and help ensure the trustworthiness of the data (Creswell; Loiselle, Profetto-McGrath, Polit, & Tatano Beck, 2004). The web-based application Dedoose (SocioCultural Research Consultants, LLC, 2018) will be utilized to assist with sorting and coding the qualitative data.

6. Ethical Considerations

There are important ethical considerations that must be considered with any type of research. This is especially true of RCTs conducted with marginalized populations (Solomon et al., 2009). Accordingly, we have endeavored to weave ethical considerations into all aspects of the study design (Solomon et al.), including our decision to utilize a CBPAR methodology and to modify the study design so that all of the participants will receive rent subsidies. Ethical approval for this study will be obtained from the St. Michael's Hospital Research and Ethics Board (REB).

6a. Benefits and Risks to Participants

All of the study participants will likely find it beneficial to receive rent subsidies. Those randomized to the intervention group may also benefit from receiving regular interactions with a mentor. Participants selected for qualitative interviews might benefit from the opportunity to share their integration-related experiences with Dr. Thulien on a regular basis. Additionally, participants may derive satisfaction from knowing that their contributions will help advance our understanding about how best to design interventions that assist formerly homeless young people to achieve meaningful social integration.

We believe theoretical justification exists for expecting that the proposed mentoring intervention is likely to produce effective outcomes; however, research ethics demands that we mitigate any potential risk to the research participants (Solomon et al., 2009). As previously mentioned, we will rely on the expertise of our community partners to screen and train the mentors. In addition, as highlighted previously, the outreach workers will work closely with the study participants and with our research team, and will alert our team if there are any concerns about a mentor-mentee relationship. These concerns will be relayed to our community partners so they can take appropriate action. The St. Michael's Hospital REB will be notified if a mentor-mentee relationship is terminated due to actions that violate the mentor-mentee code of conduct.

Participants will be assured that their participation or lack of participation in the study will not affect their relationship with our community partners.

6b. Privacy and Confidentiality

This mixed methods RCT will use multiple and varied data sources. This comprehensiveness is critical to the objectives of the study, yet may increase invasion of participant privacy. This privacy concern will be clearly communicated to potential participants, as will the measures for protecting security and confidentiality, prior to consent.

All of the data collected will be kept in strict confidence. While participants' names will appear on the consent forms, pseudonyms (created by the participants) will be used in place of their real names on all documents related to data generation, including the audio recordings and interview transcripts. A key that links each participant name with a pseudonym will be created and stored as a separate electronic file. All electronic data will be stored on a secure server at the Centre for Urban Health Solutions and only accessible by select members of the research team. The individual interviews and focus groups will be audio recorded using a password protected application on a password protected electronic device. The audio recordings from the individual interviews and focus groups will be deleted once the transcripts have been stored on the secure server and entered into Dedoose (encrypted and password-protected) (SocioCultural Research Consultants, LLC, 2018). Paper copies of the data (e.g., consent forms and standardized quantitative measures) will be stored in a locked filing cabinet at the Centre for Urban Health Solutions – an area only accessible to those with electronic key access. All paper and electronic files will be retained for a period of up to five years from study closure.

The consent form indicates that limits to confidentiality apply if a participant discloses that they intended to hurt themselves or others, or if they inform a member of the research team that someone under the age of 16 years is suffering abuse and/or neglect.

7. Dissemination

In keeping with our CBPAR methodology, we are committed to disseminating evidence *with* our community partners to build community capacity and improve the lives of the young people participating in this study (Chenail et al., 2012; Wallerstein et al., 2018). Moreover, given our use of Critical Social Theory, we are obliged to not only present our findings, but

to expose and explicate the relational processes (e.g., subjective experience of low socioeconomic position and low social class) that may be preventing formerly homeless young people from achieving meaningful social integration (Madison, 2012; Strega, 2005). With an emphasis on 'actionable' data (Chenail et al.), we anticipate disseminating our findings broadly to both academic and community-based audiences in a variety of formats ranging from scientific journal papers to oral presentations.

8. Limitations

This study has a number of limitations. First, the young people recruited for this pilot study will be a small sample of youth connected to urban-based social service providers in the province of Ontario. Thus, the findings may not be generalizable to formerly homeless young people living in other contexts and/or not connected to social service agencies. Second, the quantitative instruments are based on self-report and may involve a degree of response bias. Finally, the quantitative measures we have chosen are what we believe to be surrogate markers of meaningful social integration. Future work will likely be needed to more accurately capture this complex concept.

9. Significance

This pilot RCT study will be the first to test the impact of economic and social supports on meaningful social integration for formerly homeless young people living in market rent housing. We believe the mixed methods design will illuminate important contextual factors that must be considered if the intervention is to be scaled up and replicated elsewhere. Importantly, the CBPAR framework will incorporate the perspectives of the community, including formerly homeless young people, who are in the best position to determine what might work best in the context of their lives.

References

Altena, A. M., Brilleslijper-Kater, S. N., & Wolf, J. R. (2010). Effective interventions for homeless youth: A systematic review. *American Journal of Preventive Medicine*, *38*(6), 637-645. doi: 10.1016/j.amepre.2010.02.017.

Beck, A. T., Weissman, A., Lester, D., & Trexler, L. (1974). The measurement of pessimism: The hopelessness scale. *Journal of Consulting and Clinical Psychology, 41*(6), 639–660. doi: 10.1037/h0037562.

Brueckner, M., Green, M., & Saggers, S. (2011). The trappings of home: Young homeless people's transitions towards independent living. *Housing Studies*, *26*(1), 1-16. doi:10.1080/02673037.2010.512751.

Chenail. R. J., St. Goerge, S., Wulff, D., & Cooper, R. (2012). Action research: The methodologies. In P. L. Munhall (Ed.), *Nursing research: A qualitative perspective* (5th ed.) (pp. 455-470). Sudbury, MS: Jones & Bartlett.

Ciarolo, J. A., Edwards, D. W., Kiresuk, T. J., Newman, F. L., & Brown, T. R. (1981). *Colorado symptom index.* Washington, DC: National Institute of Mental Health.

Coren, E., Hossain, R., Pardo Pardo, J., & Bakker, B. (2016). Interventions for promoting reintegration and reducing harmful behaviour and lifestyles in street-connected children and young people. *Cochrane database of systematic reviews*, *2016*(1), 1-152. doi: 10.1002/14651858.CD009823.pub3.

Craig, P., Dieppe, P., Macintyre, S., Michie, S., Nazareth, I., & Petticrew, M. (2008). Developing and evaluating complex interventions: The new Medical Research Council guidance. *BMJ*, *337*(a1655), 1-6. doi: 10.1136/bmj.a1655.

Creswell, J.W. (2014). *Research design: Qualitative, quantitative, and mixed method approaches* (4th ed.). Thousand Oaks, CA: Sage.

Creswell, J.W., & Plano Clark, V.L. (2018). Designing and conducting mixed methods research (3rd ed.). Thousand Oaks, CA: Sage.

Dang, M. T., & Miller, E. (2013). Characteristics of natural mentoring relationships from the perspectives of homeless youth. *Journal of Child and Adolescent Psychiatric Nursing*, *26*(4), 246-253. doi: 10.1111/jcap.12038.

Denzin, N.K., & Lincoln, Y.S. (2011). *The SAGE handbook of qualitative research*. Thousand Oaks, CA: SAGE Publications Inc.

Eakin, J.M., & Mykhalovskiy, E. (2003). Reframing the evaluation of qualitative health research: Reflections on a review of appraisal guidelines in the health sciences. *Journal of Evaluation in Clinical Practice*, *9*(2), 187-194. doi: 10.1046/j.1365-2753.2003.00392.x.

Farquhar, M. C., Ewing, G., & Booth, S. (2011). Using mixed methods to develop and evaluate complex interventions in palliative care research. *Palliative Medicine*, *25*(8), 748-757. doi: 10.1177/0269216311417919.

Fletcher, R.H., Fletcher, S.W., & Fletcher, G.S. (2014). *Clinical epidemiology: The essentials* (5th ed.). Philadelphia, PA: Lippincott Williams & Wilkins.

Frederick, T., Chwalek, M., Hughes, J., Karabanow, J., & Kidd, S. (2014). How stable is stable? Defining and measuring housing stability. *Journal of Community Psychology, 42*(8), 964- 979. doi:10.1002/jcop.21665.

Gaetz, S. (2014). *Coming of age: Reimagining the response to youth homelessness in Canada.* Toronto, ON: The Canadian Homelessness Research Network Press. Retrieved from http://www.homelesshub.ca/comingofage

Gaetz, S., Dej, E., Richter, T., & Redman, M. (2016) *The state of homelessness in Canada 2016.* Toronto, ON: Canadian Observatory on Homelessness Press. Retrieved from http://www.homelesshub.ca/SOHC2016

Gaetz, S., O'Grady, B., Kidd, S., & Schwan, K. (2016). *Without a home: The national youth homelessness survey.* Toronto: Canadian Observatory on Homelessness Press. Retrieved from http://homelesshub.ca/sites/default/files/WithoutAHome-final.pdf

Gaetz, S., & Redman, M. (2016). *Federal investment in youth homelessness: Comparing Canada and the United States and a proposal for reinvestment. Canadian observatory on homelessness policy brief.* Toronto, ON: The Homeless Hub Press. Retrieved from http://homelesshub.ca/sites/default/files/Policy_Brief.pdf

Goodkind, J. R., Amer, S., Christian, C., Hess, J. M., Bybee, D., Isakson, B. L., ... Shantzek, C. (2017). Challenges and innovations in a community-based participatory randomized controlled trial. *Health Education & Behavior*, *44*(1), 123-130. doi: 10.1177/1090198116639243

Hammersley, M., & Atkinson, P. (2007). *Ethnography: Principles in Practice* (3rd ed.). London, UK: Routledge.

Hwang, S.W., & Burns, T. (2014). Health interventions for people who are homeless. *The Lancet, 384*(9953), 1541-1547. doi: 10.1016/S0140-6736(14)61133-8.

Israel, B.A., Schulz, A.J., Parker, E.A., Becker, A.B., Allen, III, Guzman, R.J., & Lichtenstein, R. (2018). Critical issues in developing and following CBPR principles. In N. Wallerstein, B. Duran, J. Oetzel, & M. Minkler (Eds.), *Community-based participatory research for health: Advancing social and health equity* (3rd ed.) (pp. 31-44). San Francisco, CA: Jossey-Bass.

Karabanow, J. (2008). Getting off the street: Exploring the process of young people's street exits. *American Behavioral Scientist, 51*(6)*,* 772-788. doi:10.1177/0002764207311987.

Karabanow, J., Carson, A., & Clement, P. (2010). *Leaving the streets: Stories of Canadian youth*. Halifax, NS: Fernwood Publishing.

Karabanow, J., Kidd, S., Frederick, T., & Hughes, J. (2016). Toward housing stability: Exiting homelessness as an emerging adult. *Journal of Sociology & Social Welfare, 43*(1), 121- 148. Retrieved from https://wmich.edu/socialworkjournal

Kawabata, M., & Gastaldo, D. (2015). The less said, the better: Interpreting silence in qualitative research. *International Journal of Qualitative Research Methods, 14*(4), 1-9. doi:10.1177/1609406915618123.

Kidd, S.A., Frederick, T., Karabanow, J., Hughes, J., Naylor, T., & Barbic, S. (2016). A mixed methods study of recently homeless youth efforts to sustain housing and stability. *Child and Adolescent Social Work Journal*, *33*(3), 207-218. doi:10.1007/s10560-015-0424.

Kozloff, N., Adair, C. E., Lazgare, L. I. P., Poremski, D., Cheung, A. H., Sandu, R., & Stergiopoulos, V. (2016). "Housing first" for homeless youth with mental illness. *Pediatrics*, *138*(4), e20161514. doi: 10.1542/peds.2016-1514

Kulik, D.M., Gaetz, S., Crowe, C., & Ford-Jones, E.L. (2011). Homeless youth's overwhelming health burden: A review of the literature. *Paediatric Child Health*, *16*(6), e43-e47. doi: 10.1093/pch/16.6e43.

Kusenbach, M. (2003). Street phenomenology: The go-along as ethnographic research tool. *Ethnography, 4*(3), 455-485. doi: 10.1177/146613810343007.

Lee, R. M., & Robbins S. B. (1995). Measuring belongingness: The social connectedness and the social assurance scales. *Journal of Counseling Psychology*, *42*(2), 232-241. doi:

10.1037/0022-0167.42.2.232.

Lewin, S., Glenton, C., & Oxman, A. D. (2009). Use of qualitative methods alongside randomised controlled trials of complex healthcare interventions: methodological study. *BMJ*, *339*(b3496), 1-7. doi: 10.1136/bmj.b3496.

Loiselle, C.G., Profetto-McGrath, J., Polit, D.F., & Tatano Beck, C.T. (2004). *Canadian essentials of nursing research*. Philadelphia, PA: Lippincott Williams & Wilkins.

Luchenski, S., Maguire, N., Aldridge, R. W., Hayward, A., Story, A., Perri, P., ... Hewett, N. (2017). What works in inclusion health: overview of effective interventions for marginalised and excluded populations. *The Lancet, 391*(10117), 266-280. doi:10.1016/S0140-6736(17)31959-1.

Madison, D.S. (2012). *Critical ethnography: Method, ethics, and performance* (2nd ed.). Thousand Oaks, CA: Sage Publication, Inc.

Mayock, P., O'Sullivan, E., & Corr, M.L. (2011). Young people exiting homelessness: An exploration of process, meaning and definition. *Housing Studies, 26*(6), 803-826. doi:10.1080/02673037.2011.593131.

McCay, E., Carter, C., Aiello, A., Quesnel, S., Langley, J., Hwang, S., ... Karabanow, J. (2015). Dialectical Behavior Therapy as a catalyst for change in street-involved youth: A mixed methods study. *Children and Youth Services Review*, *58*, 187-199. doi: 10.1016/j.childyouth.2015.09.021.

McCay, E. & Seeman, M. (1998). A scale to measure the impact of a schizophrenic illness on an individual's self-concept. *Archives of Psychiatric Nursing, 12(1),* 41-49. doi: 10.1016/S0883-9417(98)80007-1.

Milburn, N.G., Rice, E., Rotheram-Borus, M.J., Mallett, S., Rosenthal, D., Batterham, P., ... Duan, N. (2009). Adolescents exiting homelessness over two years: The risk amplification and abatement model. *Journal of Research on Adolescence, 19*(4), 762-785. doi:10.1111/j.1532-7795.2009.00610.x.

Moore, G. F., Audrey, S., Barker, M., Bond, L., Bonell, C., Hardeman, W., ... Baird, J. (2015). Process evaluation of complex interventions: Medical Research Council guidance. *BMJ, 350*(h1258), 1-6. doi: 10.1136/bmj.h1258.

Ontario Human Rights Commission. (n.d.). *Housing as a human right.* Retrieved from

http://www.ohrc.on.ca/en/right-home-report-consultation-human-rights-and-rental-housing-ontario/housing-human-right

Popay, J., Escorel, S., Hernandez, M., Johnston, H., Mathieson, J., & Rispel, L. (2008). *Understanding and tackling social exclusion: Final report to the WHO commission on social determinants of health from the social exclusion knowledge network*. Retrieved from http://www.who.int/social_determinants/themes/socialexclusion/en/

Prasad, P. (2005). *Crafting qualitative research: Working in the postpositivist traditions.* New York, NY: M.E. Sharpe.

Public Interest. (2009). *Changing patterns for street involved youth.* Toronto, ON: Author. Retrieved from http://www.worldvision.ca/Programs-and- Projects/Canadian Programs/Documents/ChangingPatternsForStreetInvolvedYouth.pdf

Quilgars, D., & Pleace, N. (2016). Housing First and Social Integration: A Realistic Aim? *Social Inclusion*, *4*(4), 5-15. doi:10.17645/si.v4i4.672.

Rosenberg, M. (1965). Society and the adolescent self-image. Princeton, NJ: Princeton University Press.

Rutman, A., Hubberstey, A., Barlow, A., & Brown, E. (2005). Supporting young people's transitions from care: Reflections on doing participatory action research with youth from care. In L. Brown & S. Strega (Eds.), *Research as resistance: Critical, Indigenous, & anti-oppressive approaches* (pp. 153-179). Toronto, ON: Canadian Scholars' Press/Women's Press.

Slesnick, N., Dashora, P., Letcher, A., Erdem, G., & Serovich, J. (2009). A review of services and interventions for runaway and homeless youth: Moving forward. *Children and Youth Services Review*, *31*(7), 732-742. doi: 10.1016/j.childyouth.2009.01.006.

SocioCultural Research Consultants, LLC. (2018). Dedoose (Version 8.0.35) [web application]. Retrieved from http://www.dedoose.com

Solar, O., & Irwin, A. (2010). *A conceptual framework for action on the social determinants of health: Social determinants of health discussion paper 2.* Geneva, Switzerland: World Health Organization Press. Retrieved from http://www.who.int/sdhconference/resources/ConceptualframeworkforactiononSDH_eng.pdf

Solomon, P., Cavanaugh, M.M., & Draine, J. (2009). *Randomized controlled trials: Design*

and implementation for community-based psychosocial interventions. New York, NY: Oxford University Press.

Stergiopoulos, V., Gozdzik, A., O'Campo, P., Holtby, A., Jeyaratnam, J., & Tsemberis, S. (2014). Housing first: Exploring participants' early support needs. *BMC Health Services Research, 14*(167), 1-15. doi:10.1186/1472-6963-14-16.

Strega, B. (2005). The view from the poststructural margins: Epistemology and methodology reconsidered. In L. Brown & S. Strega (Eds.), *Research as resistance: Critical, Indigenous, & anti-oppressive approaches* (pp. 199-235). Toronto, ON: Canadian Scholars' Press/Women's Press.

Suresh, K. P. (2011). An overview of randomization techniques: an unbiased assessment of outcome in clinical research. *Journal of human reproductive sciences*, *4*(1), 8-11. doi: 10.4103/0974-1208.82352.

Thompson, A. E., Greeson, J. K., & Brunsink, A. M. (2016). Natural mentoring among older youth in and aging out of foster care: A systematic review. *Children and Youth Services Review*, *61*, 40-50. doi: 10.1016/j.childyouth.2015.12.006.

Thulien, N. S., Gastaldo, D., Hwang, S. W., & McCay, E. (2018). The elusive goal of social integration: A critical examination of the socio-economic and psychosocial consequences experienced by homeless young people who obtain housing. *Canadian Journal of Public Health*, *109*(1), 89-98. doi: 10.17269/s41997-018-0029-6.

Toro, P. A., Rabideau, J. M. P., Bellavia, C. W., Daeschler, C. V., Wall, D. D., Thomas, D. M., & Smith, S. J. (1997). Evaluating an intervention for homeless persons: results of a field experiment. *Journal of consulting and clinical psychology*, *65*(3), 476-484. doi: 10.1037//0022-006X.65.3.476.

Urbaniak, G.C., & Plous,S. (2013). Research Randomizer (Version 4.0) [Computer software]. Retrieved from https://www.randomizer.org

Van Dam, L., Smit, D., Wildschut, B., Branje, S. J. T., Rhodes, J. E., Assink, M., & Stams, G. J. J. M. (2018). Does natural mentoring matter? A multilevel meta-analysis on the association between natural mentoring and youth outcomes. *American Journal of Community Psychology, 0,* 1-18. doi: 10.1002/ajcp.12248.

Wallerstein, N. & Duran, B. (2006). Using community-based participatory research to

address health disparities. *Health Promotion Practice, 7*(3), 312-323. doi: 10.1177/1524839906289376.

Wallerstein, N. & Duran, B. (2010). Community-based participatory research contributions to intervention research: The intersection of science and practice to improve health equity. *American Journal of Public Health, 100*(S1), S40-S46. doi: 10.2105/AJPH.2009.

Wallerstein, N., Duran, B., Oetzel, J.G., & Minkler, M. (2018). *Community-based participatory research for health: Advancing social and health equity* (3rd ed.). San Francisco, CA: Jossey-Bass.

Appendix A

Quantitative Data Collection

St. Michael's
Inspired Care. Inspiring Science. | Centre for Urban Health Solutions

Baseline Demographic Questionnaire

Pseudonym: ________________________ **Date: ______________________**

This questionnaire will help us learn a bit more about you.

1. **What is your age?** ____________

2. **Do you identify as:**
   - ☐ Male
   - ☐ Female
   - ☐ Different identity – Please specify: _________________________

3. **How do you identify your race/ethnic background?** (you can pick more than one)
   - ☐ Arab
   - ☐ Black – African
   - ☐ Black – Canadian
   - ☐ Black – Caribbean
   - ☐ Chinese
   - ☐ Filipino
   - ☐ Japanese
   - ☐ Korean
   - ☐ Latin American
   - ☐ South Asian (e.g., East Indian, Pakistani, Sri Lankan, etc.)
   - ☐ Southeast Asian (e.g., Vietnamese, Cambodian, Laotian, Thai, etc.)
   - ☐ West Asian (e.g., Iranian, Afghan, etc.)
   - ☐ White
   - ☐ Different identity – Please specify: ______________________

4. **Were you born in Canada?**
   - ☐ Yes
   - ☐ No

5. **What is your immigration status?**
   - ☐ Canadian citizen
   - ☐ Permanent resident
   - ☐ Refugee
   - ☐ Other – Please specify: ______________________

6. **Has the Children's Aid Society ever been involved with you or your family?**
   - ☐ Yes
   - ☐ No

7. **How old were you the first time you became homeless?** ____________________

8. **How many times have you tried to live on your own or with others after being homeless?**
   ____

9. **What is the highest level of education you have completed?**
   - ☐ Less than grade 9
   - ☐ Some high school
   - ☐ Completed high school
   - ☐ Some post-secondary (e.g., college, university, or vocational training <u>after high school</u>)
   - ☐ Completed post-secondary ___________________

10. **Are you in regular contact with an adult relative?** (e.g., parent, grandparent, or aunt)
    - ☐ Yes
    - ☐ No

11. **Do you have an adult in your life who is not a relative and outside the social service sector that you consider a mentor?** (e.g., someone who provides you guidance and encouragement)
    - ☐ Yes
    - ☐ No

St. Michael's
Inspired Care. Inspiring Science. | Centre for Urban Health Solutions

Education, Employment, and Income Questionnaire

Pseudonym: ________________________

Date: ______________________ **Time (circle): 0, 6, 12, 18, 24, 30**

This questionnaire will help us learn about your education, employment, and income.

Your answers will not impact your participation in this study.

1. **Are you regularly attending a school, college or university?**
   Please select "Yes – Temporary break" if you are currently on a break from this program (such as summer break), but you are scheduled to start again in the next 5 months.
   ☐ Yes
   ☐ Yes - Temporary break
   ☐ No → **Please skip to question #4**

2. **What kind of school is this?**
   ☐ High school (includes high school equivalency like getting a GED)
   ☐ College
   ☐ University
   ☐ Other – Please specify: _____________________________________

3. **How are you paying for school?**
   ☐ OSAP (Ontario Student Assistance Program)
   ☐ Other government assistance program
   ☐ Own income
   ☐ Other loan (e.g., borrowed money from family or bank loan)

4. **Are you enrolled in an on-the-job training program?**
   ☐ Yes
   ☐ No → **Please skip to question #6**

5. **If yes, how long have you been enrolled in this training program?**
   ____

6. **Are you currently employed? (includes paid on-the-job training and cash jobs)**
   ☐ Yes
   ☐ No → **Please skip to question #11**

7. **Is this a different job since your last interview six months ago?**
   ☐ Yes
   ☐ No

8. **How long have you been employed at this job?**
   ____

9. **How many hours a week do you typically work?**
   ☐ 0 – 20
   ☐ 20 – 40
   ☐ 40+

10. **How much employment income do you earn on an average month after deductions? (i.e., How much is your paycheque?)**
    ☐ Less than $1,000 per month
    ☐ $1,000 – $2,000 per month
    ☐ $2,000 – $3,000 per month
    ☐ More than $3,000 per month

11. **Are you on OW?**
    ☐ Yes
    ☐ No

12. **Are you on ODSP?**
    ☐ Yes
    ☐ No

13. **What percentage of your income (including OW/ODSP income) do you spend on rent each month?**
    ☐ Less than 25%
    ☐ 25% - 50%
    ☐ 50% - 75%
    ☐ More than 75%

St. Michael's
Inspired Care. Inspiring Science. | Centre for Urban Health Solutions

Beck's Hopelessness Scale

Pseudonym: ______________________

Date: ______________________ **Time (circle): 0, 6, 12, 18, 24, 30**

This questionnaire helps us learn how hopeful you feel. Please circle the response that describes your attitude ***for the past week.***

1. **I look forward to the future with hope and enthusiasm.**
   True/False
2. **I might as well give up because there is nothing I can do to make things better for myself.**
   True/False
3. **When things are going badly, I am helped by knowing that they can't stay that way forever.**
   True/False
4. **I can't imagine what my life would be like in ten years.**
   True/False
5. **I have enough time to accomplish the things I most want to do.**
   True/False
6. **In the future, I expect to succeed in what concerns me most.**
   True/False
7. **My future seems dark to me.**
   True/False
8. **I happen to be particularly lucky and I expect to get more of the good things in life than the average person.**
   True/False
9. **I just don't get any breaks, and there's no reason to believe that I will in the future.**
   True/False

10. My past experiences have prepared me well for my future.

True/False

11. All I can see ahead of me is unpleasantness rather than pleasantness.

True/False

12. I don’t expect to get what I really want.

True/False

13. When I look ahead to the future I expect I will be happier than I am now.

True/False

14. Things just won’t work out the way I want them to.

True/False

15. I have great faith in the future.

True/False

16. I never get what I want, so it’s foolish to want anything.

True/False

17. It is very unlikely that I will get any real satisfaction in the future.

True/False

18. The future seems vague and uncertain to me.

True/False

19. I can look forward to more good times than bad times.

True/False

20. There is no use in really trying to get something I want because I probably won’t get it.

True/False

St. Michael's
Inspired Care. Inspiring Science. | Centre for Urban Health Solutions

Community Integration Scale

Pseudonym: ______________________________

Date: ______________________

Time (circle): 0, 6, 12, 18, 24, 30

This questionnaire helps us learn how you are interacting with your community. ***Please circle your response to each question or statement****.*

Physical Integration

In the <u>past month</u> have you:

1. **Attended a movie or concert?**

   Yes No Don't remember

2. **Participated in outside sports or recreation?**

   Yes No Don't remember

3. **Gone to meet people at a restaurant or coffee shop?**

   Yes No Don't remember

4. **Participated in a community event?**

   Yes No Don't remember

5. **Gone to a place of worship or participated in a spiritual ceremony?**

   Yes No Don't remember

6. **Participated in a volunteer activity?**

   Yes No Don't remember

7. **Gone to a library?**

   Yes No Don't remember

Psychological Integration

8. **I know most of the people who live near me.**

   1 - Strongly disagree 2 - Disagree 3 - Neither 4 - Agree 5 - Strongly agree

9. **I interact with people who live near me.**

   1 - Strongly disagree 2 - Disagree 3 - Neither 4 - Agree 5 - Strongly agree

10. **I feel at home where I live.**

    1 - Strongly disagree 2 - Disagree 3 - Neither 4 - Agree 5 - Strongly agree

11. **I feel like I belong where I live.**

    1 - Strongly disagree 2 - Disagree 3 - Neither 4 - Agree 5 - Strongly agree

St. Michael's
Inspired Care. Inspiring Science. | Centre for Urban Health Solutions

Modified Colorado Symptom Index Pseudonym: ____________________

Date: ____________________ **Time (circle): 0, 6, 12, 18, 24, 30**

*This questionnaire helps us learn about your mental well-being. Please circle the response that best describes your mental well-being **for the past month**.*

1. **In the past month, how often have you felt nervous, worried, or frustrated?**
   0-Not at all 1-Once during the month 2-Several times during the month
   3-Several times a week 4-At least every day
2. **In the past month, how often have you felt depressed?**
   0-Not at all 1-Once during the month 2-Several times during the month
   3-Several times a week 4-At least every day
3. **In the past month, how often have you felt lonely?**
   0-Not at all 1-Once during the month 2-Several times during the month
   3-Several times a week 4-At least every day
4. **In the past month, how often have others told you that you acted "paranoid" or "suspicious"?**
   0-Not at all 1-Once during the month 2-Several times during the month
   3-Several times a week 4-At least every day
5. **In the past month, how often did you hear voices, or hear or see things that other people didn't think were there?**
   0-Not at all 1-Once during the month 2-Several times during the month
   3-Several times a week 4-At least every day
6. **In the past month, how often did you have trouble making up your mind, or deciding about something?**
   0-Not at all 1-Once during the month 2-Several times during the month
   3-Several times a week 4-At least every day

7. **In the past month, how often did you have trouble thinking straight, concentrating, or remembering?**

   0-Not at all   1-Once during the month   2-Several times during the month

   3-Several times a week   4-At least every day

8. **In the past month, how often did you feel that your behaviour or actions were strange or different from that of other people?**

   0-Not at all   1-Once during the month   2-Several times during the month

   3-Several times a week   4-At least every day

9. **In the past month, how often did you feel out of place or like you did not fit in?**

   0-Not at all   1-Once during the month   2-Several times during the month

   3-Several times a week   4-At least every day

10. **In the past month, how often did you forget important things?**

    0-Not at all   1-Once during the month   2-Several times during the month

    3-Several times a week   4-At least every day

11. **In the past month, how often did you have problems with thinking too fast (thoughts racing)?**

    0-Not at all   1-Once during the month   2-Several times during the month

    3-Several times a week   4-At least every day

12. **In the past month, how often did you feel suspicious or paranoid?**

    0-Not at all   1-Once during the month   2-Several times during the month

    3-Several times a week   4-At least every day

13. **In the past month, how often did you feel like hurting or killing yourself?**

    0-Not at all   1-Once during the month   2-Several times during the month

    3-Several times a week   4-At least every day

14. **In the past month, how often have you felt like seriously hurting someone else?**

    0-Not at all   1-Once during the month   2-Several times during the month

    3-Several times a week   4-At least every day

St. Michael's
Inspired Care. Inspiring Science. | Centre for Urban Health Solutions

Modified Engulfment Scale

Pseudonym: ______________________________

Date: ____________________

Time (circle): 0, 6, 12, 18, 24, 30

This questionnaire helps us learn how you see yourself in relation to your experience of being homeless. ***Please circle your response to each statement.***

1. **I get along as well as most people do.**

   1-Completely true 2-Sometimes true 3-Sometimes true and sometimes false

   4-Sometimes false 5-Completely false

2. **Because I have been homeless, I can't do things for myself the way other people can.**

   1-Completely false 2-Sometimes false 3-Sometimes true and sometimes false

   4-Sometimes true 5-Completely true

3. **In my opinion, I will always think of myself as homeless.**

   1-Completely false 2-Sometimes false 3-Sometimes true and sometimes false

   4-Sometimes true 5-Completely true

4. **I expect to have a good quality of life in the future.**

   1-Completely true 2-Sometimes true 3-Sometimes true and sometimes false

   4-Sometimes false 5-Completely false

5. **I will never be the person I was before I became homeless.**

   1-Completely false 2-Sometimes false 3-Sometimes true and sometimes false

   4-Sometimes true 5-Completely true

6. **At some point in time I will not need social services.**

   1-Completely true 2-Sometimes true 3-Sometimes true and sometimes false

   4-Sometimes false 5-Completely false

7. **I am doing well enough today to be on my own.**

   1-Completely true   2-Sometimes true   3-Sometimes true and sometimes false

   4-Sometimes false   5-Completely false

8. **To achieve the things I want, I will have to go through a change in myself.**

   1-Completely false   2-Sometimes false   3-Sometimes true and sometimes false

   4-Sometimes true   5-Completely true

9. **Right now, I am no longer the person I was before I became homeless.**

   1-Completely false   2-Sometimes false   3-Sometimes true and sometimes false

   4-Sometimes true   5-Completely true

10. **"Once a homeless person, always a homeless person."**

    1-Completely false   2-Sometimes false   3-Sometimes true and sometimes false

    4-Sometimes true   5-Completely true

11. **I will probably experience homelessness again.**

    1-Completely false   2-Sometimes false   3-Sometimes true and sometimes false

    4-Sometimes true   5-Completely true

12. **Overall, my life is normal.**

    1-Completely true   2-Sometimes true   3-Sometimes true and sometimes false

    4-Sometimes false   5-Completely false

13. **Friends and family see me as just a “homeless person".**

    1-Completely false   2-Sometimes false   3-Sometimes true and sometimes false

    4-Sometimes true   5-Completely true

14. **I am often depressed because I have been homeless.**

    1-Completely false   2-Sometimes false   3-Sometimes true and sometimes false

    4-Sometimes true   5-Completely true

15. I am basically the same person I was before I became homeless.

1-Completely true 2-Sometimes true 3-Sometimes true and sometimes false

4-Sometimes false 5-Completely false

16. I can only be friends with other homeless individuals.

1-Completely false 2-Sometimes false 3-Sometimes true and sometimes false

4-Sometimes true 5-Completely true

17. Once having been homeless, there is a good chance that it will happen again.

1-Completely false 2-Sometimes false 3-Sometimes true and sometimes false

4-Sometimes true 5-Completely true

18. I believe I am more anxious and nervous than most other people.

1-Completely false 2-Sometimes false 3-Sometimes true and sometimes false

4-Sometimes true 5-Completely true

19. Having been homeless keeps me from having close friends.

1-Completely false 2-Sometimes false 3-Sometimes true and sometimes false

4-Sometimes true 5-Completely true

20. I am afraid of losing who I am.

1-Completely false 2-Sometimes false 3-Sometimes true and sometimes false

4-Sometimes true 5-Completely true

21. I will always be different from others because I have been homeless.

1-Completely false 2-Sometimes false 3-Sometimes true and sometimes false

4-Sometimes true 5-Completely true

22. I will always have to take care not to become homeless again.

1-Completely false 2-Sometimes false 3-Sometimes true and sometimes false

4-Sometimes true 5-Completely true

23. I will be able to find work in the future.

1-Completely true 2-Sometimes true 3-Sometimes true and sometimes false

4-Sometimes false 5-Completely false

24. There is something wrong with me.

1-Completely false 2-Sometimes false 3-Sometimes true and sometimes false

4-Sometimes true 5-Completely true

25. Sometime in the future, I will earn enough money to buy more of the things I want.

1-Completely true 2-Sometimes true 3-Sometimes true and sometimes false

4-Sometimes false 5-Completely false

26. I am healthy in my body and mind.

1-Completely true 2-Sometimes true 3-Sometimes true and sometimes false

4-Sometimes false 5-Completely false

27. It is good for me to seek help if I need it.

1-Completely false 2-Sometimes false 3-Sometimes true and sometimes false

4-Sometimes true 5-Completely true

28. There are many things that I used to be able to do that I can't do now.

1-Completely false 2-Sometimes false 3-Sometimes true and sometimes false

4-Sometimes true 5-Completely true

29. I am damaged as a person by my homelessness.

1-Completely false 2-Sometimes false 3-Sometimes true and sometimes false

4-Sometimes true 5-Completely true

30. I can look forward to being married or having a steady partner if I want that for myself.

1-Completely true 2-Sometimes true 3-Sometimes true and sometimes false

4-Sometimes false 5-Completely false

St. Michael's
Inspired Care. Inspiring Science. | Centre for Urban Health Solutions

Perceived Housing Quality

Pseudonym: ______________________________

Date: ____________________

Time (circle): 0, 6, 12, 18, 24, 30

This questionnaire helps us learn what you think about your housing situation. ***Please circle your response to each question.***

1. **How do you feel about how long you will be able to live in your place?**

   1-Very dissatisfied 2-Dissatisfied 3-Neither satisfied or dissatisfied

   4-Satisfied 5-Very satisfied

2. **How do you feel about how affordable your place is?**

   1-Very dissatisfied 2-Dissatisfied 3-Neither satisfied or dissatisfied

   4-Satisfied 5-Very satisfied

3. **How would you rate your current home for safety?**

   1-Very dissatisfied 2-Dissatisfied 3-Neither satisfied or dissatisfied

   4-Satisfied 5-Very satisfied

4. **How about spaciousness?** (feeling like you have enough space to live comfortably)

   1-Very dissatisfied 2-Dissatisfied 3-Neither satisfied or dissatisfied

   4-Satisfied 5-Very satisfied

5. **How about privacy?** (feeling like you will not be disturbed by other people)

   1-Very dissatisfied 2-Dissatisfied 3-Neither satisfied or dissatisfied

   4-Satisfied 5-Very satisfied

6. **How about friendliness?** (feeling like you are in a pleasant and welcoming place)

   1-Very dissatisfied 2-Dissatisfied 3-Neither satisfied or dissatisfied

   4-Satisfied 5-Very satisfied

7. **How would you rate your current home for overall quality?**

   1-Very dissatisfied 2-Dissatisfied 3-Neither satisfied or dissatisfied

   4-Satisfied 5-Very satisfied

St. Michael's
Inspired Care. Inspiring Science. | Centre for Urban Health Solutions

Rosenberg Self-Esteem Scale **Pseudonym: ___________________________**

Date: ____________________ **Time (circle): 0, 6, 12, 18, 24, 30**

This questionnaire helps us learn how you feel about yourself. ***Please circle your response to each statement.***

1. **On the whole, I am satisfied with myself.**
   0 - Strongly disagree   1 - Disagree   2 - Agree   3 - Strongly agree
2. **At times, I think I am no good at all.**
   3 - Strongly disagree   2 - Disagree   1 - Agree   0 - Strongly agree
3. **I feel that I have a number of good qualities.**
   0 - Strongly disagree   1 - Disagree   2 - Agree   3 - Strongly agree
4. **I am able to do things as well as most other people.**
   0 - Strongly disagree   1 - Disagree   2 - Agree   3 - Strongly agree
5. **I feel I do not have much to be proud of.**
   3 - Strongly disagree   2 - Disagree   1 - Agree   0 - Strongly agree
6. **I certainly feel useless at times.**
   3 - Strongly disagree   2 - Disagree   1 - Agree   0 - Strongly agree
7. **I fell that I'm a person of worth, at least on an equal plane with others.**
   0 - Strongly disagree   1 - Disagree   2 - Agree   3 - Strongly agree
8. **I wish I could have more respect for myself.**
   3 - Strongly disagree   2 - Disagree   1 - Agree   0 - Strongly agree
9. **All in all, I am inclined to feel that I am a failure.**
   3 - Strongly disagree   2 - Disagree   1 - Agree   0 - Strongly agree
10. **I take a positive attitude toward myself.**
    0 - Strongly disagree   1 - Disagree   2 - Agree   3 - Strongly agree

St. Michael's
Inspired Care. Inspiring Science. | Centre for Urban Health Solutions

Social Connectedness Scale – Revised

Pseudonym: ____________________

Date: ____________________ **Time (circle): 0, 6, 12, 18, 24, 30**

This questionnaire helps us learn how connected you feel to the world around you.

Please circle your response to each statement.

1. **I feel comfortable in the presence of strangers.**

1-Strongly disagree 2-Disagree 3-Mildly disagree 4-Mildly agree 5-Agree 6-Strongly agree

2. **I am in tune with the world.**

1-Strongly disagree 2-Disagree 3-Mildly disagree 4-Mildly agree 5-Agree 6-Strongly agree

3. **Even among my friends, there is no sense of brother/sisterhood.**

6-Strongly disagree 5-Disagree 4-Mildly disagree 3-Mildly agree 2-Agree 1-Strongly agree

4. **I fit in well in new situations.**

1-Strongly disagree 2-Disagree 3-Mildly disagree 4-Mildly agree 5-Agree 6 -Strongly agree

5. **I feel close to people.**

1-Strongly disagree 2-Disagree 3-Mildly disagree 4-Mildly agree 5-Agree 6-Strongly agree

6. **I feel disconnected from the world around me.**

6-Strongly disagree 5-Disagree 4-Mildly disagree 3-Mildly agree 2-Agree 1-Strongly agree

7. **Even around people I know, I don't feel that I really belong.**

6-Strongly disagree 5-Disagree 4-Mildly disagree 3-Mildly agree 2-Agree 1-Strongly agree

8. **I see people as friendly and approachable.**

1-Strongly disagree 2-Disagree 3-Mildly disagree 4-Mildly agree 5-Agree 6-Strongly agree

9. **I feel like an outsider.**

6-Strongly disagree 5-Disagree 4-Mildly disagree 3-Mildly agree 2-Agree 1-Strongly agree

10. I feel understood by the people I know.

1-Strongly disagree 2-Disagree 3-Mildly disagree 4-Mildly agree 5-Agree 6-Strongly agree

11. I feel distant from people.

6-Strongly disagree 5-Disagree 4-Mildly disagree 3-Mildly agree 2-Agree 1-Strongly agree

12. I am able to relate to my peers.

1-Strongly disagree 2-Disagree 3-Mildly disagree 4-Mildly agree 5-Agree 6-Strongly agree

13. I have little sense of togetherness with my peers.

6-Strongly disagree 5-Disagree 4-Mildly disagree 3-Mildly agree 2-Agree 1-Strongly agree

14. I find myself actively involved in people's lives.

1-Strongly disagree 2-Disagree 3-Mildly disagree 4-Mildly agree 5-Agree 6-Strongly agree

15. I catch myself losing a sense of connectedness to society.

6-Strongly disagree 5-Disagree 4-Mildly disagree 3-Mildly agree 2-Agree 1-Strongly agree

16. I am able to connect with other people.

1-Strongly disagree 2-Disagree 3-Mildly disagree 4-Mildly agree 5-Agree 6-Strongly agree

17. I see myself as a loner.

6-Strongly disagree 5-Disagree 4-Mildly disagree 3-Mildly agree 2-Agree 1-Strongly agree

18. I don't feel related to most people.

6-Strongly disagree 5-Disagree 4-Mildly disagree 3-Mildly agree 2-Agree 1-Strongly agree

19. My friends feel like family.

1-Strongly disagree 2-Disagree 3-Mildly disagree 4-Mildly agree 5-Agree 6-Strongly agree

20. I don't feel I participate with anyone or any group.

6-Strongly disagree 5-Disagree 4-Mildly disagree 3-Mildly agree 2-Agree 1-Strongly agree

Appendix B

Qualitative Data Generation

St. Michael's
Inspired Care. Inspiring Science. | Centre for Urban Health Solutions

Initial Interview Guide: Individual Interviews[7]

1. ***Impact of rent subsidies***
    a) Can you describe, in general, how receiving rent subsidies has impacted your life?
    b) Can you give me an example of something you are able to do or afford now that was not possible before?
    c) Do you see yourself and/or the future differently now that you are receiving rent subsidies? If so, can you give me an example?
2. ***Impact of being randomized to intervention (mentorship) or control group***
    a) Can you describe, in general, what you think about being matched (or not) with a mentor?
    b) What sorts of things do you do (or would you do) with your mentor?
    c) Are there certain things that you wish your mentor (or a mentor) could help you with? If so, can you tell me more about that?
    d) Do you have anyone else (or just anyone) in your life that plays a similar mentor-like role?
3. ***Social integration (tangible and intangible aspects)***
    a) When you spend time around people who have not experienced homelessness, how do you feel? How about being with people who are still homeless?
    b) What do you hope your life look like in five years? Ten years?
    c) How confident are you that you can achieve what you hope for your life?
    d) Can you help me understand some of the things you think might hold you back from achieving what you hope for in life?
    e) If you had a magic wand, how would you help someone who wants to move away from being homeless?

---

[7] These questions will be used to guide the initial, semi-structured conversations with study participants. They are based on our research objectives and themes found in the literature review. Given the iterative nature of qualitative inquiry, it is expected that these questions will evolve over time.

Initial Interview Guide: Focus Groups[8]

1. ***Decision to become a mentor***
   a) Can you please share why you decided to become a mentor?
   b) Did you encounter any barriers that could have prevented you from being a mentor in this study?
   c) Did you have any reservations about becoming a mentor? If so, what helped make up your mind?

2. ***Mentorship role***
   a) Can you help me understand your overall experience so far of being a mentor?
   b) What sorts of things do you do with your mentee?
   c) Are there things you might like to do with your mentee but are not able to because of: a) Their apparent lack of interest? (e.g., visiting a local library) or b) Restrictions placed on the mentor-mentee relationship? (e.g., inviting your mentee to a family dinner)
   d) How is your mentorship role similar and/or different than what you were expecting?
   e) Can you share what you believe to be the top three most valuable traits in a mentor?
   f) What are you learning from your mentee?

3. ***Mentorship and Social integration***
   a) What sorts of things do you think could help your mentee feel more included in society?
   b) What do you think your mentee is gaining from the mentor-mentee relationship?
   c) What do you think their life will look like in five years? Ten years?
   d) If you had a magic wand, how would you help someone who wants to move away from being homeless?

[8] These questions will be used to guide the initial focus group with the mentors. They are based on our research objectives and themes found in the literature review. Given the iterative nature of qualitative inquiry, it is expected that these questions will evolve over time.

Transitioning Youth Out of Homelessness: A Mixed Methods Community-Based Pilot Randomized Controlled Trial of a Rent Subsidy and Mentoring Intervention in Three Canadian Cities

1. Background and Rationale

Young people comprise almost 20% of the homeless population in Canada (Gaetz, Dej, Richter, & Redman, 2016). It is estimated between 35,000 and 40,000 Canadian youth (ages 13 – 25) are homeless at some point during the year and at least 6,000 on any given night (Gaetz, O'Grady, Kidd, & Schwan, 2016; Gaetz & Redman, 2016).

We know a great deal about the risk factors associated with young people entering and becoming entrenched in street life (e.g., intergenerational poverty, childhood abuse, inadequate education, and limited employment opportunities), but we know much less about how to facilitate and sustain transitions off the streets (Karabanow, 2008; Kidd et al., 2016; Kulik, Gaetz, Crowe, & Ford-Jones, 2011; Mayock, O'Sullivan, & Corr, 2011). In fact, in the peer-reviewed literature, the evidence is scarce to non-existent for rigorous interventions targeting housing outcomes, life trajectories, quality of life, and social integration[1] for young people experiencing homelessness (Altena, Brilleslijiper-Kater, & Wolf, 2010; Coren, Hossain, Pardo, & Bakker, 2016; Hwang & Burns, 2014; Luchenski et al., 2017). Understanding how to create and support successful pathways out of homelessness is crucial, because once youth become entrenched in street life, it becomes much harder for them to exit homelessness and escape a life of poverty (Gaetz, 2014; Karabanow, Carson, & Clement, 2010; Milburn et al., 2009; Public Interest, 2009).

---

[1] The concept of social integration is complex and often inconsistently defined and poorly measured (Quilgars & Pleace, 2016). For the purpose of this study, we drew from the literature on the social determinants of health and social exclusion, and adopted a holistic definition of social integration, incorporating both the tangible (e.g., access to education and a living wage) and intangible (e.g., sense of connection and belonging) aspects of meaningful and equitable societal participation (Luchenski et al., 2017; Popay et al., 2008; Solar & Irwin, 2010).

Intuitively, it may seem that one important way to improve the life trajectories of young people experiencing homelessness is to provide them with a home. However, from the limited research that has been done in this area, we know that formerly homeless young people continue to experience significant challenges – particularly when it comes to mainstream social integration – even after they are 'successfully' housed (Thulien, Gastaldo, Hwang, & McCay, 2018). Moreover, these challenges seem to persist regardless of the type of housing (e.g., subsidized vs. market rent) provided (Brueckner, Green, & Saggers, 2011; Kidd et al., 2016; Kozloff et al., 2016).

Evidence from one of the most rigorous longitudinal studies with formerly homeless youth (ages 16 – 25) to date highlights that the procurement of a home does not necessarily translate into a sense of belonging or connection to mainstream society (Kidd et al., 2016). This mixed methods study of 51 formerly homeless young people living in two major urban centers in Canada showed that, despite living in stable or semi-stable accommodations (53% lived in subsidized housing), participants continued to face substantial challenges such as poverty-level incomes and limited mainstream social networks which, over the course of one year, contributed to a significant decline in hope, no gains in community integration, and a sense of being 'stuck' (Frederick, Chwalek, Hughes, Karabanow, & Kidd, 2014; Karabanow, Kidd, Frederick, & Hughes, 2016; Kidd et al.). Moreover, community integration challenges were significantly worse for participants living in independent (market rent) housing.

A sub-group analysis of 156 young people (ages 18 – 24) with mental health challenges who participated in a 24-month randomized controlled trial (RCT) of 'Housing First' (access to subsidized housing and comprehensive social service supports [e.g., treatment for mental health challenges] at home or in the community) in five Canadian cities – the largest RCT of Housing First to date – indicate similar findings of ongoing hardship despite achieving housing stability (Kozloff et al., 2016). While the young people who received the Housing First intervention achieved significantly better housing stability compared to the treatment as usual group, they did not experience any additional improvements to other outcomes such as employment, generic quality of life, and community integration relative to treatment as usual (Kozloff et al.). Notably, the same community integration scale (Stergiopoulos et al., 2014)

was used in this RCT and the aforementioned Kidd et al. study, and measures both physical integration (e.g., attending a movie or community event) and psychological integration (e.g., interactions with others and feeling like one belongs).

Findings from a ten-month ethnographic study with nine formerly homeless young people (ages 18 – 24) living in Canada's largest city also support the idea that transitioning young people out of homelessness and helping them integrate into the mainstream likely requires much more than simply providing them with a home (Thulien et al., 2018). This study is believed to be the first ethnographic study to exclusively focus on the integration experiences of formerly homeless young people living in market rent housing and showed that, despite the appearance of housing stability, the participants were living a precarious existence, attributed in part to the chronic stress and exhaustion of living in poverty and to their limited knowledge about how to move forward in life (Thulien et al.). In addition, the authors note that participants underutilized transition-related social supports (e.g., food banks and employment counseling) because these supports tended to be deficit-focused (e.g., focused on what youth did not have, not on what they had achieved) and located in areas (e.g., homeless shelters) that reminded them of their old identities as homeless youth.

As previously mentioned, little evidence exists for effective interventions that target social integration for young people who have experienced homelessness. This includes evidence on the impact of mentorship. In fact, for formal mentorship programs in general, meta-analyses have only found small overall positive effect sizes (i.e., the impact of the average mentoring program in improving youth outcomes) on the psychological, emotional, behavioral, and educational functioning of participating young people (Thompson, Greeson, & Brunsink, 2016; Van Dam et al., 2018). However, there is some emerging evidence on the benefits of 'natural mentors' – generally defined as an important, encouraging, non-parental adult that exists in a youth's social network – that may be transferrable to youth who have experienced homelessness.

A systematic review of natural mentoring for youth (ages 13 – 25) transitioning out of foster care showed that the young people benefited from a supportive adult not "tasked with

enforcing daily rules and addressing misbehavior" (p. 48) and that this intervention resulted in improved behavioral, psychosocial, and academic outcomes (Thompson et al.). The authors stress the importance of cultivating *inter*dependence (as opposed to independence) for young people leaving foster care and suggest that, while traditional natural mentoring relationships tend to emerge organically, they can be facilitated and supported programmatically as well (see https://vimeo.com/115837436).

A more recent meta-analysis of natural mentoring in youth (ages 13 – 24) also supports the notion that the presence of a natural mentor can positively impact young people (Van Dam et al., 2018). This meta-analysis included all young people (not just 'at-risk' youth) and found that, similar to the aforementioned systematic review, positive youth outcomes were particularly significant in the domains of social and emotional development, and academic and vocational functioning (Van Dam et al.). Moreover, the authors found that risk status (e.g., young people who were homeless or living in foster care) did *not* moderate these positive outcomes.

While almost all of the reviewed studies of at-risk youth in the meta-analysis and the systematic review were limited by their cross-sectional design, the results do hold promise for mentoring interventions that incorporate the positive characteristics of natural mentors (i.e., more of a friendship-like, 'coach', or 'cheerleader' role) for young people who have experienced homelessness. These findings are supported by a small (n = 23) qualitative study of natural mentoring relationships among homeless youth (ages 14 – 21) that suggests "natural mentors could feasibly serve as a bridge in a coordinated effort to assist youth out of homelessness" (Dang & Miller, 2013, p. 7).

From the limited research that has been done with young people transitioning away from homelessness, the emerging evidence seems to indicate that, while structural supports such as subsidized housing and social service providers are important, these things alone are insufficient to help young people integrate into mainstream society. As it currently stands, it appears as if the burden for achieving meaningful social integration is on the formerly homeless young people, who continue to be marginalized despite achieving stable or semi-

stable housing (Quilgars & Pleace, 2016; Thulien et al., 2018). Connecting these young people with an adult who exhibits the relationship-based components of natural mentoring that young people value most (e.g., genuine interest in their well-being and belief in their ability to succeed, a non-judgmental attitude and a willingness to listen, the provision of advice, guidance, affirmation and encouragement) (Dang & Miller, 2013; Thompson et al., 2016; Van Dam et al., 2018) may be key to helping them move forward and integrate into the mainstream.

This intervention will provide 24 young people (ages 16 – 26) who have transitioned out of homelessness and into market rent housing within the past year with rent subsidies for 24 months. Half of the young people will also receive mentorship. We chose to focus on young people living in market rent housing because, due to limited subsidized housing options, this is the reality for most young people exiting homelessness.

Initially, we proposed to our community partners a study design where only half the young people would receive rent subsidies, with the other half receiving 'treatment as usual'; however, we abandoned this idea after our community partners challenged the ethics of not providing or delaying rent subsidies for young people living a precarious existence and desperate for immediate, tangible support to help them remain in market rent housing. Consequently, we adopted the stance that, given housing is a basic human right[2], we would offer rent subsidies to *all* of the study participants. While this does move us away from the 'gold standard' in terms of measuring the impact of rent subsidies on social integration, we believe the mixed methods longitudinal design will still yield important insights in this regard. Moreover, this design adaptation reflects our deep commitment to engage in community-based participatory action research (CBPAR) – a methodology that challenges traditional epistemological assumptions of what constitutes 'good' evidence, demands researcher humility, stresses genuine and equitable academic-community partnerships, and

[2] "Adequate housing is essential to one's sense of dignity, safety, inclusion and ability to contribute to the fabric of our neighbourhoods and societies…without appropriate housing it is often not possible to get and keep employment, to recover from mental illness or other disabilities, to integrate into the community, to escape physical or emotional violence or to keep custody of children" (Ontario Human Rights Commission, n.d.).

facilitates the undertaking of research in a way that the *community* feels is most beneficial to their members (Goodkind et al., 2017; Wallerstein & Duran, 2006; Wallerstein, Duran, Oetzel, & Minkler, 2018).

Half of the young people will be randomized to receive regular mentorship from an adult mentor, tasked with helping their mentee bridge the gap between homelessness and mainstream living. While some of these mentors will not be 'natural' in the sense that these may not be pre-existing, organically-formed relationships (see 3a. Mentorship), the mentors will incorporate the key relationship-based components of natural mentoring mentioned previously, with a strong emphasis on a strengths-based approach (i.e., focus on the young person's strengths as opposed to their limitations) and the connection of participants to larger social networks (including education and employment).

Findings from this longitudinal pilot randomized controlled trial will help address the gap in our knowledge about the impact of financial support and mentorship on meaningful social integration for young people who have experienced homelessness and are living in market rent housing.

2. Study Aim and Objectives

The overarching aim of this mixed methods study is to assess whether and how rent subsidies and mentorship influence social integration outcomes for formerly homeless young people living in market rent housing in three urban settings.

Specifically, the objectives of this study are to:

1. Determine whether rent subsidies plus mentorship results in better social integration outcomes than only receiving rent subsidies with respect to: a) community integration (psychological and physical); and b) self-esteem at our primary endpoint of 18 months.
2. Determine whether rent subsidies plus mentorship results in better social integration outcomes than only receiving rent subsidies with respect to: a) social connectedness; b) hope; and c) sustained academic and vocational participation at our secondary endpoint of 18 months.

3. Explore whether rent subsidies plus mentorship results in better social integration outcomes than only receiving rent subsidies with respect to: a) income; b) perceived housing quality; c) psychiatric symptoms; and d) sense of engulfment at our exploratory endpoint of 18 months.
4. Integrate qualitative data to facilitate a fuller understanding of the quantitative data and deepen our understanding of what the study participants (young people and mentors) found most beneficial about the intervention and how it could be improved.

3. Study Design

This study will employ a convergent mixed methods design (i.e., quantitative and qualitative data are collected concurrently, and the findings combined) embedded within a RCT and a CBPAR framework (Creswell, 2014; Creswell & Plano Clark, 2018). We believe a mixed methods RCT is appropriate given the complex explanatory pathways (i.e., social and behavioral processes that may act independently and interdependently) of this intervention (Lewin, Glenton, & Oxman, 2009). In addition, the qualitative data will provide insights on contextual factors that may impact the external validity of our findings (Goodkind et al., 2017). Most importantly, this design provides a crucial (and underutilized) youth-informed perspective on social integration.

The study will be conducted in three Canadian cities: Toronto, Ontario (pop. 2.8 million); Hamilton, Ontario (pop. 552,000); and St. Catharines, Ontario (pop. 133,000). The design and implementation of this study is very much collaborative effort between our research team and the following community partners: a) Covenant House Toronto; b) Social Planning and Research Counsel of Hamilton; and c) The RAFT (St. Catharines).

All of the study participants (n = 24) will receive rent subsidies (ranging from \$400 – \$500/month)[3] for 24 months. This study includes funding for the rent subsidies and will be paid directly to the landlords by our community partners. St. Michael's Hospital will establish a service provider agreement with each of our community partners for this purpose.

[3] Given the higher cost of rent in Toronto, youth living in Toronto will receive \$500/month, while youth living in Hamilton and St. Cathatrines will receive \$400/month.

3a. Mentorship

Participants in the intervention group (n = 12) will be matched with an adult mentor recruited by one of our community partners. Each of our community partners expressed a strong desire to take the lead in the screening and recruitment of mentors as they feel they are in the best position to work with the study participants to ensure the best mentor ‘fit’. Drawing on the expertise of our community partners and sharing decision-making power is aligned with CBPAR principles and highlights our commitment to collaborative, equitable partnerships in all phases of the research process (Israel et al., 2018). Moreover, working with established community resources makes practical sense; not only will this facilitate co-learning and capacity building between the research team and our community partners (Israel et al.), but delivering the mentorship intervention under ‘real world’ conditions will provide important insights into scalability and sustainability (Wallerstein & Duran, 2010).

To build capacity between community partners, Covenant House Toronto will share their comprehensive *Mentor Program Guidelines* and *Mentor Orientation Handbook* (attached to this study protocol), which will act as a guide for all sites. These booklets cover information ranging from ideal mentor characteristics to mentor code of conduct. Each of our partners will designate one person currently serving in a leadership role within the organization to conduct one-on-one interviews with potential mentors and make the final decision (in conjunction with study participants) about mentor-mentee matches. In Hamilton and St. Catharines, this process will be undertaken by the executive director of each organization and, in Toronto, it will be done by the co-ordinator of their mentorship program.

As mentioned previously, our community partners are firm in their desire to control the mentorship screening and recruitment process, and will do so in a way that works best for each organization. That being said, all three organizations have agreed to the following preliminary screening process prior to meeting potential mentors:

- The mentor must show original documentation of passing a Vulnerable Sector police check within the past three months
- The mentor should ideally be at least five years older than the mentee
- The mentor must provide three references; one must be from a current employer

The mentors will be encouraged to incorporate the key relationship-based components of natural mentors previously described (e.g., a ‘coach’ or ‘cheerleader’ role) to assist with mainstream integration. To facilitate more of an organic, natural mentor-mentee relationship, the mentors will have more flexibility than a typical formal mentorship program in the types of activities they pursue with their mentees. For example, they will not be mandated to attend shelter-based social events. Instead, mentors will be encouraged to initiate activities that direct their mentees *away* from the shelter system (and their old identities as homeless youth) and toward the mainstream (e.g., meeting for coffee at a local university campus, touring a local library, or visiting the mentor’s place of employment during business hours). All of the mentors will meet monthly with their mentees for two years. In addition, the mentor will be encouraged to touch base with their mentee via phone or text message every week. If a mentor is unable to continue their role and there are at least six months left in the study, the study participant will be matched with a new mentor.

3b. Outreach Worker

Our community partners will match all participants with an outreach worker (already employed by each agency and considered ‘standard of care’) who will communicate regularly with the research team, help ensure the rent subsidies are being distributed appropriately, maintain an ongoing relationship with the study participants, and monitor for ‘red flags’ in participants matched in mentor-mentee relationships (e.g., mentee reluctant to meet with their mentor). Matching all of the study participants with a worker will also help ensure that everyone is receiving a fairly equal level of social support from our community partners, making it easier for the research team to discern whether the outcomes of interest are more likely attributable to mentorship rather than to varied levels of agency-based support. Moreover, a review of services and interventions designed to reduce “problem behaviors” (p. 733) (e.g., substance use and risky sexual practices) among street-involved and homeless young people (ages 12 – 24) found that researchers who had strong relationships with outreach workers and the community had more effective interventions and lower attrition rates than those who did not (Slesnick, Dashora, Letcher, Erdem, & Serovich, 2009).

3c. Methodology

As previously noted, this mixed methods RCT is embedded within a CBPAR framework. With the goal of reducing health inequities through knowledge and action, CBPAR can be a powerful tool for those working with marginalized populations (Chenail, St. George, Wulff, & Cooper, 2012; Rutman, Hubberstey, Barlow, & Brown, 2005; Wallerstein & Duran, 2006; Wallerstein et al., 2018). The ontological and epistemological assumptions underpinning CBPAR methodology are closely aligned with Critical Social Theory – that is, the belief that social conditions (e.g., socioeconomic contexts) perpetuate societal power imbalances and shape our version of 'truth' (Denzin & Lincoln, 2011; Prasad, 2005). For example, some may underestimate the social integration challenges faced by formerly homeless young people because they believe that everyone is afforded the same life chances. Thus, researchers operating within this paradigm have a goal of exposing and critiquing the inequitable (and often invisible) conditions that make it challenging for the marginalized to move forward (Strega, 2005).

We will draw on the following key principles of CBPAR as we generate and analyse data (Chenail et al., 2012; Rutman et al., 2005; Wallerstein & Duran, 2006; Wallerstein et al., 2018):

- Research participants are viewed as experts in their own lives
- Concerted effort to reduce/eliminate power imbalances between the researchers and the community
- Equal value placed on academic (researcher) knowledge and experiential (community agency/youth) knowledge
- Commitment to producing practical, 'actionable' data to build community capacity and improve/transform the lives of the research participants
- Duty to remain invested with the community beyond the life of the research project

3d. Participant Eligibility and Recruitment

Twenty-four young people ages 16 – 26 who have left homelessness within the past year and are living in market rent housing will be collaboratively recruited by our research team and

our community partners Covenant House Toronto, Social Planning and Research Counsel of Hamilton, and The RAFT (St. Catharines).

In addition to the above age and housing **inclusion** criteria, study participants must:

- Be able to provide free and informed consent
- Be fluent in English
- Plan on staying in or nearby the community in which they were recruited (Toronto, Hamilton, or St. Catharines) for the duration of the 24-month study
- Be willing to be matched with an adult mentor who has been screened and recommended by one of our three community partners (Covenant House Toronto, Social Planning and Research Counsel of Hamilton, or The RAFT) **Note:** Each study participate will be able to select their own mentor once the potential mentors have been carefully screened by our community partners (see attached *Covenant House Toronto Mentor Program: Initial Screening Application; Covenant House Toronto Mentor Program: Screening Interview;* and *Covenant House Toronto Mentor Program: Confidential Volunteer Reference Form,* which our partners in Hamilton and St. Catharines will adopt as well*).*

Young people will be **excluded** from the study if they are:

- In imminent danger of losing their housing (e.g., facing jail time or impending eviction)
- Enrolled in another study with enhanced financial and social supports
- Receiving welfare subsidies[4]

Initial introduction to the study will be done by our community partners by someone within the youths' circle of care (e.g., an outreach worker or mental health counselor). This initial introduction will be done in-person (e.g., if the young person is visiting the agency) or over-

---

[4] As it currently stands, youth on Ontario Works (OW) or the Ontario Disability Support Program (ODSP) would be penalized for receiving rent subsidies as this money would count as income. Participants would only be allowed to keep the first $200 each month, with the rest being deducted at a rate of 50 cents for every dollar provided. This would also put them at risk of losing their welfare subsidies (because they earn 'too much') and the enhanced supports (e.g., special diet allowance) that go along with these subsidies.

the-phone. Agency staff will be instructed to utilize the *Transitioning Youth Out of Homelessness Information and Recruitment Poster* (see attached) and *Telephone Script for Contacting Potential Participants: Community Partners* (see attached) to guide their conversation. If a young person expresses interest in participating in the study, agency staff will obtain verbal consent to provide the young person's name, e-mail address and/or cell phone number to Dr. Naomi Thulien. Dr. Thulien will then forward this information on to the appropriate research team member (e.g., research assistant or research coordinator), who will connect with the youth over the phone (see attached *Telephone Script for Contacting Potential Participants: Research Team Member*).

3e. Consent Process

Free and informed consent will be obtained verbally and in writing from all study participants. A concerted effort has been made to ensure the consent form is in plain language. Highlighted throughout the document is the fact that informed consent is an ongoing process and can be negotiated at any time.

All of the study participants will be screened for eligibility (see criteria above) and recommended for the study by one of our community partners (Covenant House Toronto, Social Planning and Research Counsel of Hamilton, and The RAFT). A member of the research team will call each recruited youth on the telephone and arrange to meet them at a location most convenient for the youth. Potential participants will be given a copy of the participant information and consent form to read. This document will also be reviewed verbally to ensure that those who have low literacy levels have been given the information required to give informed consent. During this process, the research team member will assess the capacity of the potential participant to provide free and informed consent. If it is unclear whether a youth is able to provide consent, the study co-investigator (Dr. Naomi Thulien) will be contacted immediately. Dr. Thulien will arrange for a qualified member of the research team to conduct a capacity assessment. If it is determined that a youth is not able to consent, they will be informed of this and they will be excluded from the study. The appropriate community partner will be notified as well.

3f. Allocation Procedure

If the participant meets the eligibility criteria, informed consent will be obtained, and the participant will be enrolled in the study. During this initial meeting, enrolled participants will participate in a baseline interview. There will be no unmasking of assignment prior to randomization. Following the baseline interview, participants at each of the three study sites (Toronto, Hamilton, and St. Catharines) will be randomized using block randomization to either the intervention (rent subsidies plus mentoring) or control (rent subsidies only) group. Randomization will be balanced by site based on random block sizes of two, four, and six. The advantage of using block randomization is to uniformly distribute participants into treatment groups within each site. Because small block sizes may increase the risk of guessing the allocation procedure and subsequently introducing bias into the enrolment procedure, random block sizes will be used to avoid selection bias (Suresh, 2011). A unique randomization schedule will be produced for each site using SAS, algorithm described in Efird (2010). We will aim to have a roughly even balance of gender[5] and ethno-racial representation at each site. Participants will be informed immediately if they have been allocated to the intervention or control group (Figure 1). In keeping with typical community-based RCTs with psychosocial interventions, 'blinding' in this study would not be pragmatic (e.g., social service providers and mentors will know if participants are in the 'treatment' group) after the baseline interviews and random assignments have been conducted (Solomon, Cavanaugh, & Draine, 2009).

Figure 1.

CONSORT (**CON**solidation of the **S**tandards **O**f **R**eporting **T**rials) Diagram of Ideal Flow of Participants Through the Study*

[5] Participants who do not identity as male or female will be randomized according to sex.

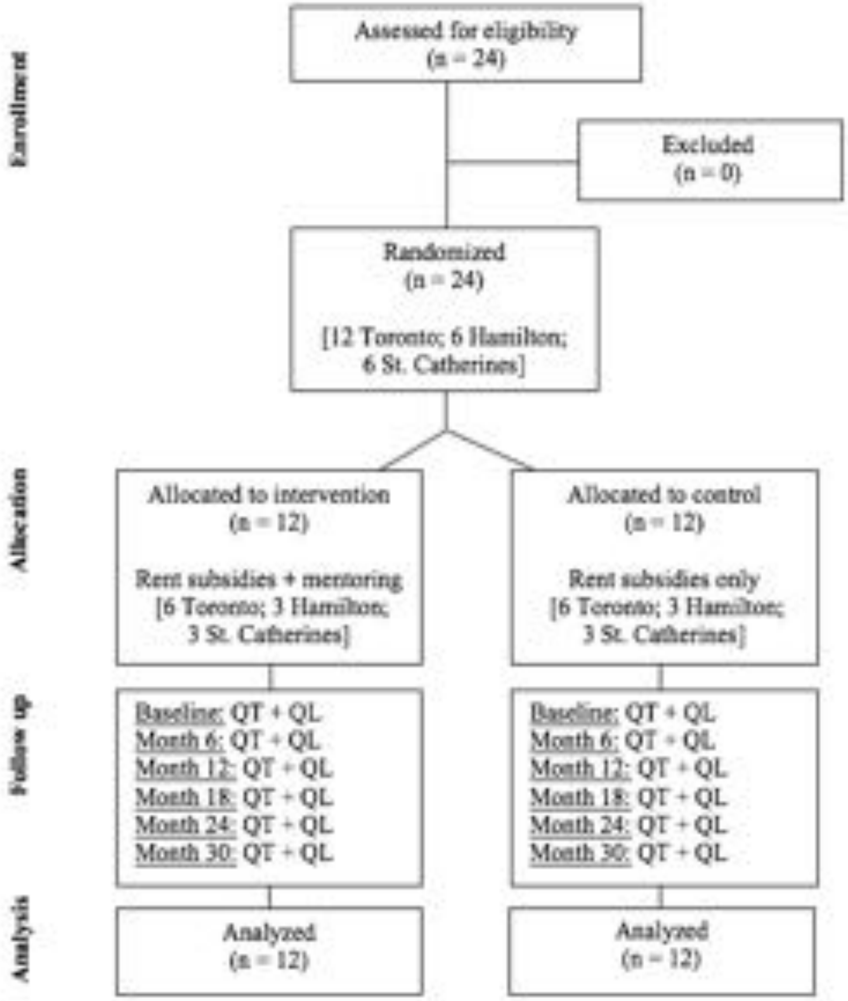

*__QT__ = quantitative measures (all participants). These will consist of six standardized measures to assess: community integration, social connectedness, engulfment, hope, self-esteem, and psychiatric symptoms. As well, participants will complete two brief questionnaires pertaining to: 1) education (includes skills training), employment, and income; and 2) perceived housing quality. **QL** = qualitative measures (12 participants). These will consist of one-on-one semi-structured interviews with the same 12 participants (six from each arm). The interview questions will explore issues related to feasibility and acceptability, and provide context to the quantitative responses.

4. Data Generation

Undertaking a mixed methods study where data is truly 'mixed' at the level of collection (not just at analysis) is challenging because it requires a solid understanding of the data generation

requirements of each research paradigm, and the interviews can take longer to conduct than those focusing on qualitative or quantitative methods alone (Farquhar, Ewing, & Booth, 2011). To minimize respondent burden, we have given careful consideration to the type of quantitative instruments chosen (e.g., number and length of time to complete) and will ensure that appropriate components of the qualitative interviews are prioritized (e.g., follow-up on changes in instrument scores) at each data generation session (Farquhar et al.). Additionally, all of the mixed method interviews will be conducted by the co-investigator, Dr. Naomi Thulien, who has expertise in conducting mixed method interviews with young people who have experienced homelessness.

4a. Study Outcomes

As mentioned previously, the mixed methods design of this pilot RCT reflects our desire to capture the complex independent and interdependent explanatory pathways of the intervention. This is especially crucial during this pilot stage, where we will be paying particular attention to feasibility, context, and unexpected mechanisms that produce change – factors that will influence study outcomes and provide important information regarding scalability and sustainability (Craig et al., 2008; Moore et al., 2015). To fully apprehend these complex explanatory pathways, "represent the best use of the data," and "provide an adequate assessment of the success or otherwise of an intervention that has effects across a range of domains" (Craig et al., p.3), we have aligned our key outcome variables (Table 1) with the Medical Research Council guidance on evaluating complex interventions and identified more than one primary outcome measure.

Table 1. Key Outcome Variables

**Variables**	**Instruments***
Community integration (psychological and physical)<br><br>Social connectedness<br>Engulfment	Community Integration Scale<br><br><br>Social Connectedness Scale – Revised<br>Modified Engulfment Scale
Self-esteem	Rosenberg Self-Esteem Scale

Hope<br>Psychiatric symptoms	Beck Hopelessness Scale<br>Modified Colorado Symptom Index
Enrollment in education (includes skills training)<br>Employment	Composite checklist
Income	Composite checklist
Perceived housing quality	Perceived Housing Quality Scale
Participant perspectives of barriers and facilitators	Individual semi-structured interviews (youth) and focus groups (mentors)<br>Composite checklist (Mentor Evaluation)

*See Table 2 for references and psychometric properties. All will be administered every six months for 30 months (except for the mentor evaluation – see Table 2).

The **primary outcome measures** for this study are: community integration (psychological and physical) and self-esteem. **Secondary outcomes** include: social connectedness, hope, and academic and vocational participation. **Exploratory outcomes** include: engulfment, psychiatric symptoms, income, perceived housing quality, and participant perspectives of intervention barriers and facilitators.

4b. Study Hypothesis

We hypothesize that, for the **primary outcome** measures of community integration and self-esteem:

1. We will observe better mean scores (community integration and self-esteem) in the participants who receive rent subsidies plus mentorship (intervention group) compared to the participants who receive rent subsidies only (control group) by our **primary endpoint of 18 months** of study participation.

We hypothesise that, for the **secondary outcome** measures of social connectedness, hope, and academic and vocational participation:

1. We will observe better mean scores (social connectedness and hope) in the intervention group relative to participants in the control group by our **secondary endpoint of 18 months** of study participation.
2. Participants in the intervention group will be more likely than the control group to demonstrate sustained engagement in academic and vocational activities (education, employment, and/or skills training) by our **secondary endpoint of 18 months** of study participation.

4c. Quantitative Measures

Quantitative data (Appendix A – Quantitative Data Collection) will be collected at **six points** in time over the course of 30 months: baseline, month six, month 12, month 18, month 24, and month 30. **Nine instruments** (Table 2) will be employed to assess the outcome variables. We have purposely chosen instruments utilized in previous research with young people who have experienced homelessness (e.g., Kidd et al., 2016; Kozloff et al., 2016; McCay et al., 2015) so that meaningful comparisons can be made across studies (Moore et al., 2015) in this nascent area of research. One instrument we do not believe has been used with this population is the Modified Engulfment Scale (McCay & Seeman, 1998). We have included engulfment as an exploratory outcome given the emerging qualitative evidence on the crucial role of identity (self-concept) in a young person's transition away from homelessness (Brueckner et al., 2011; Karabanow et al., 2016; Thulien et al., 2018).

Table 2. Quantitative Instruments

Instrument	Psychometric Information
Beck Hopelessness Scale (Beck, Weissman, Lester, & Trexler, 1974).	This 20-item scale measures motivation, expectations, and feelings about the future (internal consistency $\alpha = .93$).
Community Integration Scale (Stergiopoulos et al., 2014).	This 11-item scale measures behavioral (e.g., participation in activities) and psychological (e.g., sense of belonging) aspects of community integration. This scale was used extensively in the Chez Soi/At Home study, but psychometric properties have yet to be reported.

Education, Employment, and Income Questionnaire	This 13-item questionnaire assesses education, employment, and income. We developed this questionnaire for the study.
Mentor Evaluation Questionnaire	This 10-item questionnaire assesses mentor effectiveness. It will be completed at month 24 by those in the intervention group. We developed this questionnaire for the study in collaboration with our community partners.
Modified Colorado Symptom Index (Ciarolo et al., 1981).	This 14-item scale measures the presence and frequency of psychiatric symptoms experienced in the past month. (internal consistency α = .90 – .92).
Modified Engulfment Scale (McCay & Seeman, 1998).	This 30-item scale measures the degree to which an individual's self-concept is defined by their experience of homelessness (internal consistency α = .91). We have adapted the scale for this study, substituting "experience of homelessness" for "illness".
Perceived Housing Quality (Toro et al., 1997).	This seven-item scale measures participant perception of housing choice and quality. This scale was used extensively in the Chez Soi/At Home study, but psychometric properties have yet to be reported. We have shortened it from 10 items (Chez Soi/At Home) to seven relevant items.
Rosenberg Self-Esteem Scale (Rosenberg, 1965).	This 10-item scale measures global self-worth (internal consistency α = .77 – .88).
Social Connectedness Scale – Revised (Lee & Robbins, 1995).	This 20-item scale measures belongingness – the degree to which an individual feels connected to others (internal consistency α = .92).

4d. Qualitative Measures

Qualitative measures (see Appendix B – Qualitative Data Generation) are an important feature of this study and will consist of: **1) semi-structured individual interviews** (study participants) and **2) focus groups** (mentors).

At baseline, twelve participants (six from each arm of the study) will be invited to participate in **six semi-structured individual interviews**, which will take place at the same time as the quantitative data collection: **baseline, month six, month 12, month 18, month 24, and month 30**. Participants will be purposively selected with a goal of having input from each of the three communities and a fairly equal gender and ethno-racial representation.

All of the mentors (n = 12) will be invited to participate in **two focus groups**, which will take place at **month 12 and month 24**.

The questions posed during the semi-structured interviews and focus groups will be guided by the study objectives, but will be conversational and exploratory in nature with particular attention to understanding *how* mentoring and/or rent subsidies influence social integration outcomes for formerly homeless young people living in market rent housing. Given the emergent nature of qualitative inquiry (Denzin & Lincoln, 2011; Eakin & Mykhalovskiy, 2003), we expect the interview and focus group questions to evolve over time as key preliminary themes begin to surface. It is anticipated that the individual interviews (including quantitative data collection, which will consistently take place first) will last approximately 60 – 75 minutes, and the focus groups approximately 60 – 90 minutes. The individual interviews and the focus groups will be conducted by Dr. Naomi Thulien at locations most convenient for those participating. To get a better sense of each young person's living situation and to minimize researcher – participant power imbalance (Israel et al., 2018), Dr. Thulien will suggest that the individual interviews take place in or nearby the young people's homes. The individual interviews and focus groups will be audio recorded and transcribed verbatim.

4e. Honoraria

All of the study participants will be paid an honorarium of $20 at each of the six quantitative data collection points. Those participating in semi-structured interviews will be paid an additional $30 at each interview. This amount was based on the co-investigator's previous experience with this population and after consulting with our community partners.

5. Data Analysis

One major critique of mixed methods RCTs is that, typically, there is no true integration (i.e., 'mixing') of quantitative and qualitative findings at the level of analysis or interpretation (Lewin, Glenton, & Oxman, 2009). Moreover, it is often unclear whether or how the quantitative and qualitative researchers have worked together to maximize the potential synergies between these different approaches (Lewin et al.). With this in mind, our study team, consisting of researchers with quantitative and qualitative expertise, worked together to develop this study protocol and anticipate meeting quarterly to discuss the emerging analysis and to explore (and follow up on) similarities or discrepancies between the quantitative and qualitative data.

5a. Quantitative Data

All analyses will be performed using the intention-to-treat principle; that is, all participants will be included and analyzed in the groups they were originally randomized. Baseline characteristics of the intervention and control groups will be summarized using descriptive statistics (i.e., mean, standard deviation, median and interquartile range for continuous variables, and frequencies and proportions for categorical variables). We will also calculate descriptive statistics for outcomes at each study time point, and will explore differences in trajectories from baseline to 30 months follow-up between intervention and control groups using scatterplots and box-plots. Differences with 95% confidence intervals in continuous outcomes at 18 months (psychological community integration, self-esteem, social connectedness, hope, perceived housing quality, psychiatric symptoms, and sense of engulfment) between participants who received rent subsidies plus mentorship and participants who only received rent subsidies will be estimated using Analysis of Covariance (i.e., linear regression models), including an indicator of intervention group and the baseline value of the outcome. We will perform regression diagnostics and will repeat analyses using

the non-parametric Wilcoxon rank-sum test if there are extreme outliers or influential observations. Groups will be compared with respect to count outcomes at 18 months (physical community integration) using graphical tools and the non-parametric Wilcoxon rank-sum test. For binary outcomes at 18 months (sustained academic and vocational participation, and income above low income cut-off[6]), differences in proportions with 95% confidence intervals will be estimated and tested using the chi-square or Fisher's exact test. Given the small sample size of this pilot randomized trial, all results will be interpreted with caution and with the intention of generating data and hypotheses for conducting a larger trial.

All efforts will be made to reduce participants' attrition and drop-out. As mentioned previously, we believe our strong relationship with the outreach workers and community agencies will help minimize loss to follow-up (Slesnick et al., 2009). In addition, we have made it very clear in the participant information and consent form that participants in the rent subsidies plus mentorship arm may continue in the study (receiving only rent subsidies) if they are unable to continue in a mentor-mentee relationship.

5b. Qualitative Data

In keeping with the emergent, iterative nature of research using a qualitative design (Denzin & Lincoln, 2011; Eakin & Mykhalovskiy, 2003), data analysis and interpretation will begin immediately after the first qualitative data generation session (at baseline). The semi-structured individual interviews and focus groups will be audio recorded and transcribed verbatim. In order to conduct a more nuanced analysis of the data, the transcriptionist will be instructed to note short responses, uncooperative tones, and literal silence (Eakin & Mykhalovskiy; Kawabata & Gastaldo, 2015). Prior to each subsequent qualitative data generation session, members of the research team will conduct a preliminary data analysis, reading the interview transcripts multiple times, separating the data into coded segments, making analytic memos beside sections of the transcripts, identifying emerging themes (and comparing/contrasting these between respondents), and compiling new questions (Creswell, 2014; Denzin & Lincoln). Those participating in the individual interviews and the focus

[6] Based on family and community size.

groups will be asked for their perspectives on the emerging interpretations at each visit and these perspectives will play a key role in helping shape the data analysis and help ensure the trustworthiness of the data (Creswell; Loiselle, Profetto-McGrath, Polit, & Tatano Beck, 2004). The web-based application Dedoose (SocioCultural Research Consultants, LLC, 2018) will be utilized to assist with sorting and coding the qualitative data.

6. Ethical Considerations

There are important ethical considerations that must be considered with any type of research. This is especially true of RCTs conducted with marginalized populations (Solomon et al., 2009). Accordingly, we have endeavored to weave ethical considerations into all aspects of the study design (Solomon et al.), including our decision to utilize a CBPAR methodology and to modify the study design so that all of the participants will receive rent subsidies. Ethical approval for this study will be obtained from the Providence St. Joseph's and St. Michael's Healthcare Research Ethics Board (REB).

6a. Benefits and Risks to Participants

All of the study participants will likely find it beneficial to receive rent subsidies. Those randomized to the intervention group may also benefit from receiving regular interactions with a mentor. Participants selected for qualitative interviews might benefit from the opportunity to share their integration-related experiences with Dr. Thulien on a regular basis. Additionally, participants may derive satisfaction from knowing that their contributions will help advance our understanding about how best to design interventions that assist formerly homeless young people to achieve meaningful social integration.

We believe theoretical justification exists for expecting that the proposed mentoring intervention is likely to produce effective outcomes; however, research ethics demands that we mitigate any potential risk to the research participants (Solomon et al., 2009). As previously mentioned, we will rely on the expertise of our community partners to screen and train the mentors. In addition, as highlighted previously, the outreach workers will work closely with the study participants and with our research team, and will alert our team if there are any concerns about a mentor-mentee relationship. These concerns will be relayed to our

community partners so they can take appropriate action. The Providence St. Joseph's and St. Michael's Healthcare REB will be notified if a mentor-mentee relationship is terminated due to actions that violate the mentor-mentee code of conduct.

Participants will be assured that their participation or lack of participation in the study will not negatively impact their relationship with our community partners or their ability to access services at St. Michael's Hospital or support from other social services agencies (e.g., OW/ODSP).

6b. Privacy and Confidentiality

This mixed methods RCT will use multiple and varied data sources. This comprehensiveness is critical to the objectives of the study, yet may increase invasion of participant privacy. This privacy concern will be clearly communicated to potential participants, as will the measures for protecting security and confidentiality, prior to consent.

All of the data collected will be kept in strict confidence. While participants' names will appear on the consent forms, pseudonyms (created by the participants) will be used in place of their real names on all documents related to data generation, including the audio recordings and interview transcripts. A key that links each participant name with a pseudonym will be created and stored as a separate electronic file. All electronic data will be stored on a secure server at the Centre for Urban Health Solutions and only accessible by select members of the research team. The individual interviews and focus groups will be audio recorded using a password protected application on a password protected electronic device. The audio recordings from the individual interviews and focus groups will be deleted once the transcripts have been stored on the secure server and entered into Dedoose (encrypted and password-protected) (SocioCultural Research Consultants, LLC, 2018). Paper copies of the data (e.g., consent forms and standardized quantitative measures) will be stored in a locked filing cabinet at the Centre for Urban Health Solutions – an area only accessible to those with electronic key access. All paper and electronic files will be retained for a period of up to five years from study closure.

The consent form indicates that limits to confidentiality apply if a participant discloses that they intended to hurt themselves or others, or if they inform a member of the research team that someone under the age of 16 years is suffering abuse and/or neglect.

7. Dissemination

In keeping with our CBPAR methodology, we are committed to disseminating evidence *with* our community partners to build community capacity and improve the lives of the young people participating in this study (Chenail et al., 2012; Wallerstein et al., 2018). Moreover, given our use of Critical Social Theory, we are obliged to not only present our findings, but to expose and explicate the relational processes (e.g., subjective experience of low socioeconomic position and low social class) that may be preventing formerly homeless young people from achieving meaningful social integration (Madison, 2012; Strega, 2005). With an emphasis on 'actionable' data (Chenail et al.), we anticipate disseminating our findings broadly to both academic and community-based audiences in a variety of formats ranging from scientific journal papers to oral presentations.

8. Limitations

This study has a number of limitations. First, the young people recruited for this pilot study will be a small sample of youth connected to urban-based social service providers in the province of Ontario. Thus, the findings may not be generalizable to formerly homeless young people living in other contexts and/or not connected to social service agencies. Second, the quantitative instruments are based on self-report and may involve a degree of response bias. Finally, the quantitative measures we have chosen are what we believe to be surrogate markers of meaningful social integration. Future work will likely be needed to more accurately capture this complex concept.

9. Significance

This pilot RCT study will be the first to test the impact of economic and social supports on meaningful social integration for formerly homeless young people living in market rent housing. We believe the mixed methods design will illuminate important contextual factors that must be considered if the intervention is to be scaled up and replicated elsewhere.

Importantly, the CBPAR framework will incorporate the perspectives of the community, including formerly homeless young people, who are in the best position to determine what might work best in the context of their lives.

References

Altena, A. M., Brilleslijper-Kater, S. N., & Wolf, J. R. (2010). Effective interventions for homeless youth: A systematic review. *American Journal of Preventive Medicine*, *38*(6), 637-645. doi: 10.1016/j.amepre.2010.02.017.

Beck, A. T., Weissman, A., Lester, D., & Trexler, L. (1974). The measurement of pessimism: The hopelessness scale. *Journal of Consulting and Clinical Psychology, 41*(6), 639–660. doi: 10.1037/h0037562.

Brueckner, M., Green, M., & Saggers, S. (2011). The trappings of home: Young homeless people's transitions towards independent living. *Housing Studies*, *26*(1), 1-16. doi:10.1080/02673037.2010.512751.

Chenail. R. J., St. Goerge, S., Wulff, D., & Cooper, R. (2012). Action research: The methodologies. In P. L. Munhall (Ed.), *Nursing research: A qualitative perspective* (5th ed.) (pp. 455-470). Sudbury, MS: Jones & Bartlett.

Ciarolo, J. A., Edwards, D. W., Kiresuk, T. J., Newman, F. L., & Brown, T. R. (1981). *Colorado symptom index.* Washington, DC: National Institute of Mental Health.

Coren, E., Hossain, R., Pardo Pardo, J., & Bakker, B. (2016). Interventions for promoting reintegration and reducing harmful behaviour and lifestyles in street-connected children and young people. *Cochrane database of systematic reviews*, *2016*(1), 1-152. doi: 10.1002/14651858.CD009823.pub3.

Craig, P., Dieppe, P., Macintyre, S., Michie, S., Nazareth, I., & Petticrew, M. (2008). Developing and evaluating complex interventions: The new Medical Research Council guidance. *BMJ*, *337*(a1655), 1-6. doi: 10.1136/bmj.a1655.

Creswell, J.W. (2014). *Research design: Qualitative, quantitative, and mixed method approaches* (4th ed.). Thousand Oaks, CA: Sage.

Creswell, J.W., & Plano Clark, V.L. (2018). Designing and conducting mixed methods research (3rd ed.). Thousand Oaks, CA: Sage.

Dang, M. T., & Miller, E. (2013). Characteristics of natural mentoring relationships from the perspectives of homeless youth. *Journal of Child and Adolescent Psychiatric Nursing*, *26*(4), 246-253. doi: 10.1111/jcap.12038.

Denzin, N.K., & Lincoln, Y.S. (2011). *The SAGE handbook of qualitative research*. Thousand Oaks, CA: SAGE Publications Inc.

Eakin, J.M., & Mykhalovskiy, E. (2003). Reframing the evaluation of qualitative health research: Reflections on a review of appraisal guidelines in the health sciences. *Journal of Evaluation in Clinical Practice*, *9*(2), 187-194. doi: 10.1046/j.1365-2753.2003.00392.x.

Efird, J. (2011). Block randomization with randomly selected block sizes. *International Journal of Environmental Research and Public Health, 8(1),* 15-20. doi: 10.3390/ijerph8010015.

Farquhar, M. C., Ewing, G., & Booth, S. (2011). Using mixed methods to develop and evaluate complex interventions in palliative care research. *Palliative Medicine*, *25*(8), 748-757. doi: 10.1177/0269216311417919.

Fletcher, R.H., Fletcher, S.W., & Fletcher, G.S. (2014). *Clinical epidemiology: The essentials* (5th ed.). Philadelphia, PA: Lippincott Williams & Wilkins.

Frederick, T., Chwalek, M., Hughes, J., Karabanow, J., & Kidd, S. (2014). How stable is stable? Defining and measuring housing stability. *Journal of Community Psychology, 42*(8), 964- 979. doi:10.1002/jcop.21665.

Gaetz, S. (2014). *Coming of age: Reimagining the response to youth homelessness in Canada.* Toronto, ON: The Canadian Homelessness Research Network Press. Retrieved from http://www.homelesshub.ca/comingofage

Gaetz, S., Dej, E., Richter, T., & Redman, M. (2016) *The state of homelessness in Canada 2016.* Toronto, ON: Canadian Observatory on Homelessness Press. Retrieved from http://www.homelesshub.ca/SOHC2016

Gaetz, S., O'Grady, B., Kidd, S., & Schwan, K. (2016). *Without a home: The national youth homelessness survey.* Toronto: Canadian Observatory on Homelessness Press. Retrieved from http://homelesshub.ca/sites/default/files/WithoutAHome-final.pdf

Gaetz, S., & Redman, M. (2016). *Federal investment in youth homelessness: Comparing Canada and the United States and a proposal for reinvestment. Canadian observatory on homelessness policy brief.* Toronto, ON: The Homeless Hub Press. Retrieved from http://homelesshub.ca/sites/default/files/Policy_Brief.pdf

Goodkind, J. R., Amer, S., Christian, C., Hess, J. M., Bybee, D., Isakson, B. L., ...

Shantzek, C. (2017). Challenges and innovations in a community-based participatory randomized controlled trial. *Health Education & Behavior*, *44*(1), 123-130. doi: 10.1177/1090198116639243

Hammersley, M., & Atkinson, P. (2007). *Ethnography: Principles in Practice* (3rd ed.). London, UK: Routledge.

Hwang, S.W., & Burns, T. (2014). Health interventions for people who are homeless. *The Lancet, 384*(9953), 1541-1547. doi: 10.1016/S0140-6736(14)61133-8.

Israel, B.A., Schulz, A.J., Parker, E.A., Becker, A.B., Allen, III, Guzman, R.J., & Lichtenstein, R. (2018). Critical issues in developing and following CBPR principles. In N. Wallerstein, B. Duran, J. Oetzel, & M. Minkler (Eds.), *Community-based participatory research for health: Advancing social and health equity* (3rd ed.) (pp. 31-44). San Francisco, CA: Jossey-Bass.

Karabanow, J. (2008). Getting off the street: Exploring the process of young people's street exits. *American Behavioral Scientist, 51*(6)*,* 772-788. doi:10.1177/0002764207311987.

Karabanow, J., Carson, A., & Clement, P. (2010). *Leaving the streets: Stories of Canadian youth*. Halifax, NS: Fernwood Publishing.

Karabanow, J., Kidd, S., Frederick, T., & Hughes, J. (2016). Toward housing stability: Exiting homelessness as an emerging adult. *Journal of Sociology & Social Welfare, 43*(1), 121- 148. Retrieved from https://wmich.edu/socialworkjournal

Kawabata, M., & Gastaldo, D. (2015). The less said, the better: Interpreting silence in qualitative research. *International Journal of Qualitative Research Methods, 14*(4), 1-9. doi:10.1177/1609406915618123.

Kidd, S.A., Frederick, T., Karabanow, J., Hughes, J., Naylor, T., & Barbic, S. (2016). A mixed methods study of recently homeless youth efforts to sustain housing and stability. *Child and Adolescent Social Work Journal*, *33*(3), 207-218. doi:10.1007/s10560-015-0424.

Kozloff, N., Adair, C. E., Lazgare, L. I. P., Poremski, D., Cheung, A. H., Sandu, R., & Stergiopoulos, V. (2016). "Housing first" for homeless youth with mental illness. *Pediatrics*, *138*(4), e20161514. doi: 10.1542/peds.2016-1514

Kulik, D.M., Gaetz, S., Crowe, C., & Ford-Jones, E.L. (2011). Homeless youth's overwhelming health burden: A review of the literature. *Paediatric Child Health*, *16*(6),

e43-e47. doi: 10.1093/pch/16.6e43.

Kusenbach, M. (2003). Street phenomenology: The go-along as ethnographic research tool. *Ethnography, 4*(3), 455-485. doi: 10.1177/146613810343007.

Lee, R. M., & Robbins S. B. (1995). Measuring belongingness: The social connectedness and the social assurance scales. *Journal of Counseling Psychology*, *42*(2), 232-241. doi: 10.1037/0022-0167.42.2.232.

Lewin, S., Glenton, C., & Oxman, A. D. (2009). Use of qualitative methods alongside randomised controlled trials of complex healthcare interventions: methodological study. *BMJ*, *339*(b3496), 1-7. doi: 10.1136/bmj.b3496.

Loiselle, C.G., Profetto-McGrath, J., Polit, D.F., & Tatano Beck, C.T. (2004). *Canadian essentials of nursing research*. Philadelphia, PA: Lippincott Williams & Wilkins.

Luchenski, S., Maguire, N., Aldridge, R. W., Hayward, A., Story, A., Perri, P., ... Hewett, N. (2017). What works in inclusion health: overview of effective interventions for marginalised and excluded populations. *The Lancet*, *391*(10117), 266-280. doi:10.1016/ S0140-6736(17)31959-1.

Madison, D.S. (2012). *Critical ethnography: Method, ethics, and performance* (2nd ed.). Thousand Oaks, CA: Sage Publication, Inc.

Mayock, P., O'Sullivan, E., & Corr, M.L. (2011). Young people exiting homelessness: An exploration of process, meaning and definition. *Housing Studies, 26*(6), 803-826. doi:10.1080/02673037.2011.593131.

McCay, E., Carter, C., Aiello, A., Quesnel, S., Langley, J., Hwang, S., ... Karabanow, J. (2015). Dialectical Behavior Therapy as a catalyst for change in street-involved youth: A mixed methods study. *Children and Youth Services Review*, *58*, 187-199. doi: 10.1016/j.childyouth.2015.09.021.

McCay, E. & Seeman, M. (1998). A scale to measure the impact of a schizophrenic illness on an individual's self-concept. *Archives of Psychiatric Nursing, 12(1),* 41-49. doi: 10.1016/S0883-9417(98)80007-1.

Milburn, N.G., Rice, E., Rotheram-Borus, M.J., Mallett, S., Rosenthal, D., Batterham, P., ... Duan, N. (2009). Adolescents exiting homelessness over two years: The risk amplification and abatement model. *Journal of Research on Adolescence, 19*(4), 762-785. doi:10.1111/j.1532-7795.2009.00610.x.

Moore, G. F., Audrey, S., Barker, M., Bond, L., Bonell, C., Hardeman, W., ... Baird, J. (2015). Process evaluation of complex interventions: Medical Research Council guidance. *BMJ, 350*(h1258), 1-6. doi: 10.1136/bmj.h1258.

Ontario Human Rights Commission. (n.d.). *Housing as a human right.* Retrieved from http://www.ohrc.on.ca/en/right-home-report-consultation-human-rights-and-rental-housing-ontario/housing-human-right

Popay, J., Escorel, S., Hernandez, M., Johnston, H., Mathieson, J., & Rispel, L. (2008). *Understanding and tackling social exclusion: Final report to the WHO commission on social determinants of health from the social exclusion knowledge network*. Retrieved from http://www.who.int/social_determinants/themes/socialexclusion/en/

Prasad, P. (2005). *Crafting qualitative research: Working in the postpositivist traditions.* New York, NY: M.E. Sharpe.

Public Interest. (2009). *Changing patterns for street involved youth.* Toronto, ON: Author. Retrieved from http://www.worldvision.ca/Programs-and- Projects/Canadian Programs/Documents/ChangingPatternsForStreetInvolvedYouth.pdf

Quilgars, D., & Pleace, N. (2016). Housing First and Social Integration: A Realistic Aim? *Social Inclusion*, *4*(4), 5-15. doi:10.17645/si.v4i4.672.

Rosenberg, M. (1965). Society and the adolescent self-image. Princeton, NJ: Princeton University Press.

Rutman, A., Hubberstey, A., Barlow, A., & Brown, E. (2005). Supporting young people's transitions from care: Reflections on doing participatory action research with youth from care. In L. Brown & S. Strega (Eds.), *Research as resistance: Critical, Indigenous, & anti-oppressive approaches* (pp. 153-179). Toronto, ON: Canadian Scholars' Press/Women's Press.

Slesnick, N., Dashora, P., Letcher, A., Erdem, G., & Serovich, J. (2009). A review of services and interventions for runaway and homeless youth: Moving forward. *Children and Youth Services Review*, *31*(7), 732-742. doi: 10.1016/j.childyouth.2009.01.006.

SocioCultural Research Consultants, LLC. (2018). Dedoose (Version 8.0.35) [web application]. Retrieved from http://www.dedoose.com

Solar, O., & Irwin, A. (2010). *A conceptual framework for action on the social determinants*

of health: Social determinants of health discussion paper 2. Geneva, Switzerland: World Health Organization Press. Retrieved from http://www.who.int/sdhconference/resources/ConceptualframeworkforactiononSDH_eng.pdf

Solomon, P., Cavanaugh, M.M., & Draine, J. (2009). *Randomized controlled trials: Design and implementation for community-based psychosocial interventions.* New York, NY: Oxford University Press.

Stergiopoulos, V., Gozdzik, A., O'Campo, P., Holtby, A., Jeyaratnam, J., & Tsemberis, S. (2014). Housing first: Exploring participants' early support needs. *BMC Health Services Research, 14*(167), 1-15. doi:10.1186/1472-6963-14-16.

Strega, B. (2005). The view from the poststructural margins: Epistemology and methodology reconsidered. In L. Brown & S. Strega (Eds.), *Research as resistance: Critical, Indigenous, & anti-oppressive approaches* (pp. 199-235). Toronto, ON: Canadian Scholars' Press/Women's Press.

Suresh, K. P. (2011). An overview of randomization techniques: an unbiased assessment of outcome in clinical research. *Journal of human reproductive sciences*, *4*(1), 8-11. doi: 10.4103/0974-1208.82352.

Thompson, A. E., Greeson, J. K., & Brunsink, A. M. (2016). Natural mentoring among older youth in and aging out of foster care: A systematic review. *Children and Youth Services Review*, *61*, 40-50. doi: 10.1016/j.childyouth.2015.12.006.

Thulien, N. S., Gastaldo, D., Hwang, S. W., & McCay, E. (2018). The elusive goal of social integration: A critical examination of the socio-economic and psychosocial consequences experienced by homeless young people who obtain housing. *Canadian Journal of Public Health*, *109*(1), 89-98. doi: 10.17269/s41997-018-0029-6.

Toro, P. A., Rabideau, J. M. P., Bellavia, C. W., Daeschler, C. V., Wall, D. D., Thomas, D. M., & Smith, S. J. (1997). Evaluating an intervention for homeless persons: results of a field experiment. *Journal of consulting and clinical psychology*, *65*(3), 476-484. doi: 10.1037//0022-006X.65.3.476.

Van Dam, L., Smit, D., Wildschut, B., Branje, S. J. T., Rhodes, J. E., Assink, M., & Stams, G. J. J. M. (2018). Does natural mentoring matter? A multilevel meta-analysis on the association between natural mentoring and youth outcomes. *American Journal of*

Community Psychology, 0, 1-18. doi: 10.1002/ajcp.12248.

Wallerstein, N. & Duran, B. (2006). Using community-based participatory research to address health disparities. *Health Promotion Practice, 7*(3), 312-323. doi: 10.1177/1524839906289376.

Wallerstein, N. & Duran, B. (2010). Community-based participatory research contributions to intervention research: The intersection of science and practice to improve health equity. *American Journal of Public Health, 100*(S1), S40-S46. doi: 10.2105/AJPH.2009.

Wallerstein, N., Duran, B., Oetzel, J.G., & Minkler, M. (2018). *Community-based participatory research for health: Advancing social and health equity* (3rd ed.). San Francisco, CA: Jossey-Bass.

Transitioning Youth Out of Homelessness: A Mixed Methods Community-Based Pilot Randomized Controlled Trial of a Rent Subsidy and Mentoring Intervention in Three Canadian Cities

1. Background and Rationale

Young people comprise almost 20% of the homeless population in Canada (Gaetz, Dej, Richter, & Redman, 2016). It is estimated between 35,000 and 40,000 Canadian youth (ages 13 – 25) are homeless at some point during the year and at least 6,000 on any given night (Gaetz, O'Grady, Kidd, & Schwan, 2016; Gaetz & Redman, 2016).

We know a great deal about the risk factors associated with young people entering and becoming entrenched in street life (e.g., intergenerational poverty, childhood abuse, inadequate education, and limited employment opportunities), but we know much less about how to facilitate and sustain transitions off the streets (Karabanow, 2008; Kidd et al., 2016; Kulik, Gaetz, Crowe, & Ford-Jones, 2011; Mayock, O'Sullivan, & Corr, 2011). In fact, in the peer-reviewed literature, the evidence is scarce to non-existent for rigorous interventions targeting housing outcomes, life trajectories, quality of life, and social integration[1] for young people experiencing homelessness (Altena, Brilleslijiper-Kater, & Wolf, 2010; Coren, Hossain, Pardo, & Bakker, 2016; Hwang & Burns, 2014; Luchenski et al., 2017). Understanding how to create and support successful pathways out of homelessness is crucial, because once youth become entrenched in street life, it becomes much harder for them to exit homelessness and escape a life of poverty (Gaetz, 2014; Karabanow, Carson, & Clement, 2010; Milburn et al., 2009; Public Interest, 2009).

[1] The concept of social integration is complex and often inconsistently defined and poorly measured (Quilgars & Pleace, 2016). For the purpose of this study, we drew from the literature on the social determinants of health and social exclusion, and adopted a holistic definition of social integration, incorporating both the tangible (e.g., access to education and a living wage) and intangible (e.g., sense of connection and belonging) aspects of meaningful and equitable societal participation (Luchenski et al., 2017; Popay et al., 2008; Solar & Irwin, 2010).

Intuitively, it may seem that one important way to improve the life trajectories of young people experiencing homelessness is to provide them with a home. However, from the limited research that has been done in this area, we know that formerly homeless young people continue to experience significant challenges – particularly when it comes to mainstream social integration – even after they are 'successfully' housed (Thulien, Gastaldo, Hwang, & McCay, 2018). Moreover, these challenges seem to persist regardless of the type of housing (e.g., subsidized vs. market rent) provided (Brueckner, Green, & Saggers, 2011; Kidd et al., 2016; Kozloff et al., 2016).

Evidence from one of the most rigorous longitudinal studies with formerly homeless youth (ages 16 – 25) to date highlights that the procurement of a home does not necessarily translate into a sense of belonging or connection to mainstream society (Kidd et al., 2016). This mixed methods study of 51 formerly homeless young people living in two major urban centers in Canada showed that, despite living in stable or semi-stable accommodations (53% lived in subsidized housing), participants continued to face substantial challenges such as poverty-level incomes and limited mainstream social networks which, over the course of one year, contributed to a significant decline in hope, no gains in community integration, and a sense of being 'stuck' (Frederick, Chwalek, Hughes, Karabanow, & Kidd, 2014; Karabanow, Kidd, Frederick, & Hughes, 2016; Kidd et al.). Moreover, community integration challenges were significantly worse for participants living in independent (market rent) housing.

A sub-group analysis of 156 young people (ages 18 – 24) with mental health challenges who participated in a 24-month randomized controlled trial (RCT) of 'Housing First' (access to subsidized housing and comprehensive social service supports [e.g., treatment for mental health challenges] at home or in the community) in five Canadian cities – the largest RCT of Housing First to date – indicate similar findings of ongoing hardship despite achieving housing stability (Kozloff et al., 2016). While the young people who received the Housing First intervention achieved significantly better housing stability compared to the treatment as usual group, they did not experience any additional improvements to other outcomes such as employment, generic quality of life, and community integration relative to treatment as usual (Kozloff et al.). Notably, the same community integration scale (Stergiopoulos et al., 2014)

was used in this RCT and the aforementioned Kidd et al. study, and measures both physical integration (e.g., attending a movie or community event) and psychological integration (e.g., interactions with others and feeling like one belongs).

Findings from a ten-month ethnographic study with nine formerly homeless young people (ages 18 – 24) living in Canada's largest city also support the idea that transitioning young people out of homelessness and helping them integrate into the mainstream likely requires much more than simply providing them with a home (Thulien et al., 2018). This study is believed to be the first ethnographic study to exclusively focus on the integration experiences of formerly homeless young people living in market rent housing and showed that, despite the appearance of housing stability, the participants were living a precarious existence, attributed in part to the chronic stress and exhaustion of living in poverty and to their limited knowledge about how to move forward in life (Thulien et al.). In addition, the authors note that participants underutilized transition-related social supports (e.g., food banks and employment counseling) because these supports tended to be deficit-focused (e.g., focused on what youth did not have, not on what they had achieved) and located in areas (e.g., homeless shelters) that reminded them of their old identities as homeless youth.

As previously mentioned, little evidence exists for effective interventions that target social integration for young people who have experienced homelessness. This includes evidence on the impact of mentorship. In fact, for formal mentorship programs in general, meta-analyses have only found small overall positive effect sizes (i.e., the impact of the average mentoring program in improving youth outcomes) on the psychological, emotional, behavioral, and educational functioning of participating young people (Thompson, Greeson, & Brunsink, 2016; Van Dam et al., 2018). However, there is some emerging evidence on the benefits of 'natural mentors' – generally defined as an important, encouraging, non-parental adult that exists in a youth's social network – that may be transferrable to youth who have experienced homelessness.

A systematic review of natural mentoring for youth (ages 13 – 25) transitioning out of foster care showed that the young people benefited from a supportive adult not "tasked with

enforcing daily rules and addressing misbehavior" (p. 48) and that this intervention resulted in improved behavioral, psychosocial, and academic outcomes (Thompson et al.). The authors stress the importance of cultivating *inter*dependence (as opposed to independence) for young people leaving foster care and suggest that, while traditional natural mentoring relationships tend to emerge organically, they can be facilitated and supported programmatically as well (see https://vimeo.com/115837436).

A more recent meta-analysis of natural mentoring in youth (ages 13 – 24) also supports the notion that the presence of a natural mentor can positively impact young people (Van Dam et al., 2018). This meta-analysis included all young people (not just 'at-risk' youth) and found that, similar to the aforementioned systematic review, positive youth outcomes were particularly significant in the domains of social and emotional development, and academic and vocational functioning (Van Dam et al.). Moreover, the authors found that risk status (e.g., young people who were homeless or living in foster care) did *not* moderate these positive outcomes.

While almost all of the reviewed studies of at-risk youth in the meta-analysis and the systematic review were limited by their cross-sectional design, the results do hold promise for mentoring interventions that incorporate the positive characteristics of natural mentors (i.e., more of a friendship-like, 'coach', or 'cheerleader' role) for young people who have experienced homelessness. These findings are supported by a small (n = 23) qualitative study of natural mentoring relationships among homeless youth (ages 14 – 21) that suggests "natural mentors could feasibly serve as a bridge in a coordinated effort to assist youth out of homelessness" (Dang & Miller, 2013, p. 7).

From the limited research that has been done with young people transitioning away from homelessness, the emerging evidence seems to indicate that, while structural supports such as subsidized housing and social service providers are important, these things alone are insufficient to help young people integrate into mainstream society. As it currently stands, it appears as if the burden for achieving meaningful social integration is on the formerly homeless young people, who continue to be marginalized despite achieving stable or semi-

stable housing (Quilgars & Pleace, 2016; Thulien et al., 2018). Connecting these young people with an adult who exhibits the relationship-based components of natural mentoring that young people value most (e.g., genuine interest in their well-being and belief in their ability to succeed, a non-judgmental attitude and a willingness to listen, the provision of advice, guidance, affirmation and encouragement) (Dang & Miller, 2013; Thompson et al., 2016; Van Dam et al., 2018) may be key to helping them move forward and integrate into the mainstream.

This intervention will provide 24 young people (ages 16 – 26) who have transitioned out of homelessness and into market rent housing within the past year with rent subsidies for 24 months. Half of the young people will also receive mentorship. We chose to focus on young people living in market rent housing because, due to limited subsidized housing options, this is the reality for most young people exiting homelessness.

Initially, we proposed to our community partners a study design where only half the young people would receive rent subsidies, with the other half receiving 'treatment as usual'; however, we abandoned this idea after our community partners challenged the ethics of not providing or delaying rent subsidies for young people living a precarious existence and desperate for immediate, tangible support to help them remain in market rent housing. Consequently, we adopted the stance that, given housing is a basic human right[2], we would offer rent subsidies to *all* of the study participants. While this does move us away from the 'gold standard' in terms of measuring the impact of rent subsidies on social integration, we believe the mixed methods longitudinal design will still yield important insights in this regard. Moreover, this design adaptation reflects our deep commitment to engage in community-based participatory action research (CBPAR) – a methodology that challenges traditional epistemological assumptions of what constitutes 'good' evidence, demands researcher humility, stresses genuine and equitable academic-community partnerships, and

---

[2] "Adequate housing is essential to one's sense of dignity, safety, inclusion and ability to contribute to the fabric of our neighbourhoods and societies…without appropriate housing it is often not possible to get and keep employment, to recover from mental illness or other disabilities, to integrate into the community, to escape physical or emotional violence or to keep custody of children" (Ontario Human Rights Commission, n.d.).

facilitates the undertaking of research in a way that the *community* feels is most beneficial to their members (Goodkind et al., 2017; Wallerstein & Duran, 2006; Wallerstein, Duran, Oetzel, & Minkler, 2018).

Half of the young people will be randomized to receive regular mentorship from an adult mentor, tasked with helping their mentee bridge the gap between homelessness and mainstream living. While some of these mentors will not be 'natural' in the sense that these may not be pre-existing, organically-formed relationships (see 3a. Mentorship), the mentors will incorporate the key relationship-based components of natural mentoring mentioned previously, with a strong emphasis on a strengths-based approach (i.e., focus on the young person's strengths as opposed to their limitations) and the connection of participants to larger social networks (including education and employment).

Findings from this longitudinal pilot randomized controlled trial will help address the gap in our knowledge about the impact of financial support and mentorship on meaningful social integration for young people who have experienced homelessness and are living in market rent housing.

2. Study Aim and Objectives

The overarching aim of this mixed methods study is to assess whether and how rent subsidies and mentorship influence social integration outcomes for formerly homeless young people living in market rent housing in three urban settings.

Specifically, the objectives of this study are to:

1. Determine whether rent subsidies plus mentorship results in better social integration outcomes than only receiving rent subsidies with respect to: a) community integration (psychological and physical); and b) self-esteem at our primary endpoint of 18 months.
2. Determine whether rent subsidies plus mentorship results in better social integration outcomes than only receiving rent subsidies with respect to: a) social connectedness; b) hope; and c) sustained academic and vocational participation at our secondary endpoint of 18 months.

3. Explore whether rent subsidies plus mentorship results in better social integration outcomes than only receiving rent subsidies with respect to: a) income; b) perceived housing quality; c) psychiatric symptoms; and d) sense of engulfment at our exploratory endpoint of 18 months.
4. Integrate qualitative data to facilitate a fuller understanding of the quantitative data and deepen our understanding of what the study participants (young people and mentors) found most beneficial about the intervention and how it could be improved.

3. Study Design

This study will employ a convergent mixed methods design (i.e., quantitative and qualitative data are collected concurrently, and the findings combined) embedded within a RCT and a CBPAR framework (Creswell, 2014; Creswell & Plano Clark, 2018). We believe a mixed methods RCT is appropriate given the complex explanatory pathways (i.e., social and behavioral processes that may act independently and interdependently) of this intervention (Lewin, Glenton, & Oxman, 2009). In addition, the qualitative data will provide insights on contextual factors that may impact the external validity of our findings (Goodkind et al., 2017). Most importantly, this design provides a crucial (and underutilized) youth-informed perspective on social integration.

The study will be conducted in three Canadian cities: Toronto, Ontario (pop. 2.8 million); Hamilton, Ontario (pop. 552,000); and St. Catharines, Ontario (pop. 133,000). The design and implementation of this study is very much collaborative effort between our research team and the following community partners: a) Covenant House Toronto; b) Living Rock Ministries; and c) The RAFT (St. Catharines).

All of the study participants (n = 24) will receive rent subsidies (ranging from \$400 – \$500/month)[3] for 24 months. This study includes funding for the rent subsidies and will be paid directly to the landlords by our community partners. St. Michael's Hospital will establish a service provider agreement with each of our community partners for this purpose.

[3] Given the higher cost of rent in Toronto, youth living in Toronto will receive \$500/month, while youth living in Hamilton and St. Cathatrines will receive \$400/month.

3a. Mentorship

Participants in the intervention group (n = 12) will be matched with an adult mentor recruited by one of our community partners. Each of our community partners expressed a strong desire to take the lead in the screening and recruitment of mentors as they feel they are in the best position to work with the study participants to ensure the best mentor 'fit'. Drawing on the expertise of our community partners and sharing decision-making power is aligned with CBPAR principles and highlights our commitment to collaborative, equitable partnerships in all phases of the research process (Israel et al., 2018). Moreover, working with established community resources makes practical sense; not only will this facilitate co-learning and capacity building between the research team and our community partners (Israel et al.), but delivering the mentorship intervention under 'real world' conditions will provide important insights into scalability and sustainability (Wallerstein & Duran, 2010).

To build capacity between community partners, Covenant House Toronto will share their comprehensive *Mentor Program Guidelines* and *Mentor Orientation Handbook* (attached to this study protocol), which will act as a guide for all sites. These booklets cover information ranging from ideal mentor characteristics to mentor code of conduct. Each of our partners will designate one person currently serving in a leadership role within the organization to conduct one-on-one interviews with potential mentors and make the final decision (in conjunction with study participants) about mentor-mentee matches. In Hamilton and St. Catharines, this process will be undertaken by the executive director of each organization and, in Toronto, it will be done by the co-ordinator of their mentorship program.

As mentioned previously, our community partners are firm in their desire to control the mentorship screening and recruitment process, and will do so in a way that works best for each organization. That being said, all three organizations have agreed to the following preliminary screening process prior to meeting potential mentors:

- The mentor must show original documentation of passing a Vulnerable Sector police check within the past three months
- The mentor should ideally be at least five years older than the mentee

- The mentor must provide three references; one must be from a current employer

The mentors will be encouraged to incorporate the key relationship-based components of natural mentors previously described (e.g., a 'coach' or 'cheerleader' role) to assist with mainstream integration. To facilitate more of an organic, natural mentor-mentee relationship, the mentors will have more flexibility than a typical formal mentorship program in the types of activities they pursue with their mentees. For example, they will not be mandated to attend shelter-based social events. Instead, mentors will be encouraged to initiate activities that direct their mentees *away* from the shelter system (and their old identities as homeless youth) and toward the mainstream (e.g., meeting for coffee at a local university campus, touring a local library, or visiting the mentor's place of employment during business hours). All of the mentors will meet monthly with their mentees for two years. In addition, the mentor will be encouraged to touch base with their mentee via phone or text message every week. If a mentor is unable to continue their role and there are at least six months left in the study, the study participant will be matched with a new mentor.

3b. Outreach Worker

Our community partners will match all participants with an outreach worker (already employed by each agency and considered 'standard of care') who will communicate regularly with the research team, help ensure the rent subsidies are being distributed appropriately, maintain an ongoing relationship with the study participants, and monitor for 'red flags' in participants matched in mentor-mentee relationships (e.g., mentee reluctant to meet with their mentor). Matching all of the study participants with a worker will also help ensure that everyone is receiving a fairly equal level of social support from our community partners, making it easier for the research team to discern whether the outcomes of interest are more likely attributable to mentorship rather than to varied levels of agency-based support. Moreover, a review of services and interventions designed to reduce "problem behaviors" (p. 733) (e.g., substance use and risky sexual practices) among street-involved and homeless young people (ages 12 – 24) found that researchers who had strong relationships with outreach workers and the community had more effective interventions and lower attrition rates than those who did not (Slesnick, Dashora, Letcher, Erdem, & Serovich, 2009).

3c. Methodology

As previously noted, this mixed methods RCT is embedded within a CBPAR framework. With the goal of reducing health inequities through knowledge and action, CBPAR can be a powerful tool for those working with marginalized populations (Chenail, St. George, Wulff, & Cooper, 2012; Rutman, Hubberstey, Barlow, & Brown, 2005; Wallerstein & Duran, 2006; Wallerstein et al., 2018). The ontological and epistemological assumptions underpinning CBPAR methodology are closely aligned with Critical Social Theory – that is, the belief that social conditions (e.g., socioeconomic contexts) perpetuate societal power imbalances and shape our version of 'truth' (Denzin & Lincoln, 2011; Prasad, 2005). For example, some may underestimate the social integration challenges faced by formerly homeless young people because they believe that everyone is afforded the same life chances. Thus, researchers operating within this paradigm have a goal of exposing and critiquing the inequitable (and often invisible) conditions that make it challenging for the marginalized to move forward (Strega, 2005).

We will draw on the following key principles of CBPAR as we generate and analyse data (Chenail et al., 2012; Rutman et al., 2005; Wallerstein & Duran, 2006; Wallerstein et al., 2018):

- Research participants are viewed as experts in their own lives
- Concerted effort to reduce/eliminate power imbalances between the researchers and the community
- Equal value placed on academic (researcher) knowledge and experiential (community agency/youth) knowledge
- Commitment to producing practical, 'actionable' data to build community capacity and improve/transform the lives of the research participants
- Duty to remain invested with the community beyond the life of the research project

3d. Participant Eligibility and Recruitment

Twenty-four young people ages 16 – 26 who have left homelessness within the past year and are living in market rent housing will be collaboratively recruited by our research team and

our community partners Covenant House Toronto, Living Rock Ministries, and The RAFT (St. Catharines). We will aim to have a roughly even balance of gender and ethno-racial representation at each site.

In addition to the above age and housing **inclusion** criteria, study participants must:

- Be able to provide free and informed consent
- Be fluent in English
- Plan on staying in or nearby the community in which they were recruited (Toronto, Hamilton, or St. Catharines) for the duration of the 24-month study
- Be willing to be matched with an adult mentor who has been screened and recommended by one of our three community partners (Covenant House Toronto, Living Rock Ministries, or The RAFT) **Note:** Each study participate will be able to select their own mentor once the potential mentors have been carefully screened by our community partners (see attached *Covenant House Toronto Mentor Program: Initial Screening Application; Covenant House Toronto Mentor Program: Screening Interview;* and *Covenant House Toronto Mentor Program: Confidential Volunteer Reference Form,* which our partners in Hamilton and St. Catharines will adopt as well*).*

Young people will be **excluded** from the study if they are:

- In imminent danger of losing their housing (e.g., facing jail time or impending eviction)
- Enrolled in another study with enhanced financial and social supports

Initial introduction to the study will be done by our community partners by someone within the youths' circle of care (e.g., an outreach worker or mental health counselor). This initial introduction will be done in-person (e.g., if the young person is visiting the agency) or over-the-phone. Agency staff will be instructed to utilize the *Transitioning Youth Out of Homelessness Information and Recruitment Poster* (see attached) and *Telephone Script for Contacting Potential Participants: Community Partners* (see attached) to guide their conversation. If a young person expresses interest in participating in the study, agency staff will obtain verbal consent to provide the young person's name, e-mail address and/or cell phone number to Dr. Naomi Thulien. Dr. Thulien will then forward this information on to the

appropriate research team member (e.g., research assistant or research coordinator), who will connect with the youth over the phone (see attached *Telephone Script for Contacting Potential Participants: Research Team Member*).

3e. Consent Process

Free and informed consent will be obtained verbally and in writing from all study participants. A concerted effort has been made to ensure the consent form is in plain language. Highlighted throughout the document is the fact that informed consent is an ongoing process and can be negotiated at any time.

All of the study participants will be screened for eligibility (see criteria above) and recommended for the study by one of our community partners (Covenant House Toronto, Living Rock Ministries, and The RAFT). A member of the research team will call each recruited youth on the telephone and arrange to meet them at a location most convenient for the youth. Potential participants will be given a copy of the participant information and consent form to read. This document will also be reviewed verbally to ensure that those who have low literacy levels have been given the information required to give informed consent. During this process, the research team member will assess the capacity of the potential participant to provide free and informed consent. If it is unclear whether a youth is able to provide consent, the study co-investigator (Dr. Naomi Thulien) will be contacted immediately. Dr. Thulien will arrange for a qualified member of the research team to conduct a capacity assessment. If it is determined that a youth is not able to consent, they will be informed of this and they will be excluded from the study. The appropriate community partner will be notified as well.

3f. Allocation Procedure

If the participant meets the eligibility criteria, informed consent will be obtained, and the participant will be enrolled in the study. During this initial meeting, enrolled participants will participate in a baseline interview. There will be no unmasking of assignment prior to randomization. Following the baseline interview, participants at each of the three study sites (Toronto, Hamilton, and St. Catharines) will be randomized (stratified by site) to either the

intervention (rent subsidies plus mentoring) or control (rent subsidies only) group via computer using the software program Research Randomizer (Urbaniak & Plous, 2013). Participants will be informed immediately if they have been allocated to the intervention or control group (Figure 1). In keeping with typical community-based RCTs with psychosocial interventions, 'blinding' in this study would not be pragmatic (e.g., social service providers and mentors will know if participants are in the 'treatment' group) after the baseline interviews and random assignments have been conducted (Solomon, Cavanaugh, & Draine, 2009).

Figure 1.

CONSORT (**CON**solidation of the **S**tandards **O**f **R**eporting **T**rials) Diagram of Ideal Flow of Participants Through the Study*

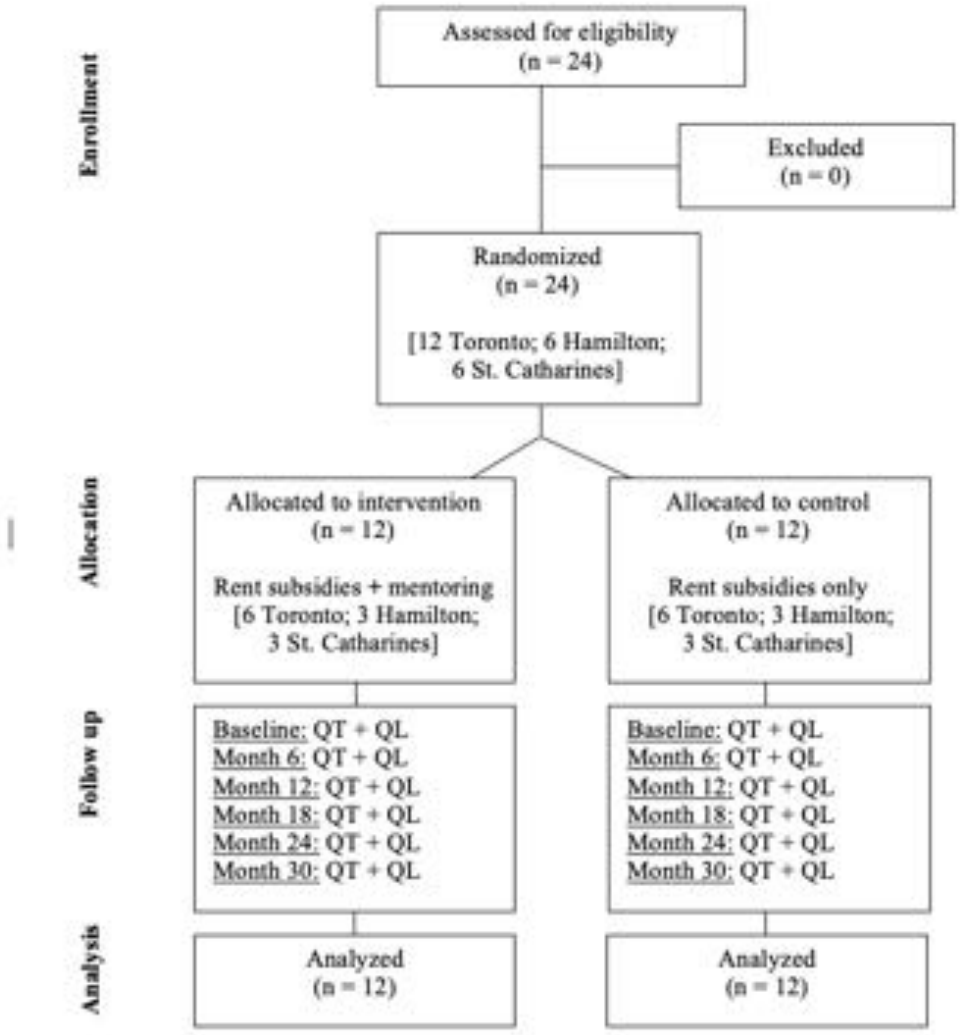

*__QT__ = quantitative measures (all participants). These will consist of six standardized measures to assess: community integration, social connectedness, engulfment, hope, self-esteem, and psychiatric symptoms. As well, participants will complete two brief questionnaires pertaining to: 1) education (includes skills training), employment, and income; and 2) perceived housing quality. **QL** = qualitative measures (12 participants). These will consist of one-on-one semi-structured interviews with the same 12 participants (six from each arm). The interview questions will explore issues related to feasibility and acceptability, and provide context to the quantitative responses.

4. Data Generation

Undertaking a mixed methods study where data is truly 'mixed' at the level of collection (not just at analysis) is challenging because it requires a solid understanding of the data generation requirements of each research paradigm, and the interviews can take longer to conduct than

those focusing on qualitative or quantitative methods alone (Farquhar, Ewing, & Booth, 2011). To minimize respondent burden, we have given careful consideration to the type of quantitative instruments chosen (e.g., number and length of time to complete) and will ensure that appropriate components of the qualitative interviews are prioritized (e.g., follow-up on changes in instrument scores) at each data generation session (Farquhar et al.). Additionally, all of the mixed method interviews will be conducted by the co-investigator, Dr. Naomi Thulien, who has expertise in conducting mixed method interviews with young people who have experienced homelessness.

4a. Study Outcomes

As mentioned previously, the mixed methods design of this pilot RCT reflects our desire to capture the complex independent and interdependent explanatory pathways of the intervention. This is especially crucial during this pilot stage, where we will be paying particular attention to feasibility, context, and unexpected mechanisms that produce change – factors that will influence study outcomes and provide important information regarding scalability and sustainability (Craig et al., 2008; Moore et al., 2015). To fully apprehend these complex explanatory pathways, "represent the best use of the data," and "provide an adequate assessment of the success or otherwise of an intervention that has effects across a range of domains" (Craig et al., p.3), we have aligned our key outcome variables (Table 1) with the Medical Research Council guidance on evaluating complex interventions and identified more than one primary outcome measure.

Table 1. Key Outcome Variables

**Variables**	**Instruments***
Community integration (psychological and physical)<br><br>Social connectedness<br>Engulfment	Community Integration Scale<br><br><br>Social Connectedness Scale – Revised<br>Modified Engulfment Scale
Self-esteem	Rosenberg Self-Esteem Scale

Hope<br>Psychiatric symptoms	Beck Hopelessness Scale<br>Modified Colorado Symptom Index
Enrollment in education (includes skills training)<br>Employment	Composite checklist
Income	Composite checklist
Perceived housing quality	Perceived Housing Quality Scale
Participant perspectives of barriers and facilitators	Individual semi-structured interviews (youth) and focus groups (mentors)<br>Composite checklist (Mentor Evaluation)

*See Table 2 for references and psychometric properties. All will be administered every six months for 30 months (except for the mentor evaluation – see Table 2).

The **primary outcome measures** for this study are: community integration (psychological and physical) and self-esteem. **Secondary outcomes** include: social connectedness, hope, and academic and vocational participation. **Exploratory outcomes** include: engulfment, psychiatric symptoms, income, perceived housing quality, and participant perspectives of intervention barriers and facilitators.

4b. Study Hypothesis

We hypothesize that, for the **primary outcome** measures of community integration and self-esteem:

1. We will observe better mean scores (community integration and self-esteem) in the participants who receive rent subsidies plus mentorship (intervention group) compared to the participants who receive rent subsidies only (control group) by our **primary endpoint of 18 months** of study participation.

We hypothesise that, for the **secondary outcome** measures of social connectedness, hope, and academic and vocational participation:

1. We will observe better mean scores (social connectedness and hope) in the intervention group relative to participants in the control group by our **secondary endpoint of 18 months** of study participation.
2. Participants in the intervention group will be more likely than the control group to demonstrate sustained engagement in academic and vocational activities (education, employment, and/or skills training) by our **secondary endpoint of 18 months** of study participation.

4c. Quantitative Measures

Quantitative data (Appendix A – Quantitative Data Collection) will be collected at **six points** in time over the course of 30 months: baseline, month six, month 12, month 18, month 24, and month 30. **Nine instruments** (Table 2) will be employed to assess the outcome variables. We have purposely chosen instruments utilized in previous research with young people who have experienced homelessness (e.g., Kidd et al., 2016; Kozloff et al., 2016; McCay et al., 2015) so that meaningful comparisons can be made across studies (Moore et al., 2015) in this nascent area of research. One instrument we do not believe has been used with this population is the Modified Engulfment Scale (McCay & Seeman, 1998). We have included engulfment as an exploratory outcome given the emerging qualitative evidence on the crucial role of identity (self-concept) in a young person's transition away from homelessness (Brueckner et al., 2011; Karabanow et al., 2016; Thulien et al., 2018).

Table 2. Quantitative Instruments

**Instrument**	**Psychometric Information**
Beck Hopelessness Scale (Beck, Weissman, Lester, & Trexler, 1974).	This 20-item scale measures motivation, expectations, and feelings about the future (internal consistency $\alpha = .93$).
Community Integration Scale (Stergiopoulos et al., 2014).	This 11-item scale measures behavioral (e.g., participation in activities) and psychological (e.g., sense of belonging) aspects of community integration. This scale was used extensively in the Chez Soi/At Home study, but psychometric properties have yet to be reported.

Education, Employment, and Income Questionnaire	This 13-item questionnaire assesses education, employment, and income. We developed this questionnaire for the study.
Mentor Evaluation Questionnaire	This 10-item questionnaire assesses mentor effectiveness. It will be completed at month 24 by those in the intervention group. We developed this questionnaire for the study in collaboration with our community partners.
Modified Colorado Symptom Index (Ciarolo et al., 1981).	This 14-item scale measures the presence and frequency of psychiatric symptoms experienced in the past month. (internal consistency α = .90 – .92).
Modified Engulfment Scale (McCay & Seeman, 1998).	This 30-item scale measures the degree to which an individual's self-concept is defined by their experience of homelessness (internal consistency α = .91). We have adapted the scale for this study, substituting "experience of homelessness" for "illness".
Perceived Housing Quality (Toro et al., 1997).	This seven-item scale measures participant perception of housing choice and quality. This scale was used extensively in the Chez Soi/At Home study, but psychometric properties have yet to be reported. We have shortened it from 10 items (Chez Soi/At Home) to seven relevant items.
Rosenberg Self-Esteem Scale (Rosenberg, 1965).	This 10-item scale measures global self-worth (internal consistency α = .77 – .88).
Social Connectedness Scale – Revised (Lee & Robbins, 1995).	This 20-item scale measures belongingness – the degree to which an individual feels connected to others (internal consistency α = .92).

4d. Qualitative Measures

Qualitative measures (see Appendix B – Qualitative Data Generation) are an important feature of this study and will consist of: **1) semi-structured individual interviews** (study participants) and **2) focus groups** (mentors).

At baseline, twelve participants (six from each arm of the study) will be invited to participate in **six semi-structured individual interviews**, which will take place at the same time as the quantitative data collection: **baseline, month six, month 12, month 18, month 24, and month 30**. Participants will be purposively selected with a goal of having input from each of the three communities and a fairly equal gender and ethno-racial representation.

All of the mentors (n = 12) will be invited to participate in **two focus groups**, which will take place at **month 12 and month 24**.

The questions posed during the semi-structured interviews and focus groups will be guided by the study objectives, but will be conversational and exploratory in nature with particular attention to understanding *how* mentoring and/or rent subsidies influence social integration outcomes for formerly homeless young people living in market rent housing. Given the emergent nature of qualitative inquiry (Denzin & Lincoln, 2011; Eakin & Mykhalovskiy, 2003), we expect the interview and focus group questions to evolve over time as key preliminary themes begin to surface. It is anticipated that the individual interviews (including quantitative data collection, which will consistently take place first) will last approximately 60 – 75 minutes, and the focus groups approximately 60 – 90 minutes. The individual interviews and the focus groups will be conducted by Dr. Naomi Thulien at locations most convenient for those participating. To get a better sense of each young person's living situation and to minimize researcher – participant power imbalance (Israel et al., 2018), Dr. Thulien will suggest that the individual interviews take place in or nearby the young people's homes. The individual interviews and focus groups will be audio recorded and transcribed verbatim.

4e. Honoraria

All of the study participants will be paid an honorarium of $20 at each of the six quantitative data collection points. Those participating in semi-structured interviews will be paid an additional $30 at each interview. This amount was based on the co-investigator's previous experience with this population and after consulting with our community partners.

5. Data Analysis

One major critique of mixed methods RCTs is that, typically, there is no true integration (i.e., 'mixing') of quantitative and qualitative findings at the level of analysis or interpretation (Lewin, Glenton, & Oxman, 2009). Moreover, it is often unclear whether or how the quantitative and qualitative researchers have worked together to maximize the potential synergies between these different approaches (Lewin et al.). With this in mind, our study team, consisting of researchers with quantitative and qualitative expertise, worked together to develop this study protocol and anticipate meeting quarterly to discuss the emerging analysis and to explore (and follow up on) similarities or discrepancies between the quantitative and qualitative data.

5a. Quantitative Data

All analyses will be performed using the intention-to-treat principle; that is, all participants will be included and analyzed in the groups they were originally randomized. Baseline characteristics of the intervention and control groups will be summarized using descriptive statistics (i.e., mean, standard deviation, median and interquartile range for continuous variables, and frequencies and proportions for categorical variables). We will also calculate descriptive statistics for outcomes at each study time point, and will explore differences in trajectories from baseline to 30 months follow-up between intervention and control groups using scatterplots and box-plots. Differences with 95% confidence intervals in continuous outcomes at 18 months (psychological community integration, self-esteem, social connectedness, hope, perceived housing quality, psychiatric symptoms, and sense of engulfment) between participants who received rent subsidies plus mentorship and participants who only received rent subsidies will be estimated using Analysis of Covariance (i.e., linear regression models), including an indicator of intervention group and the baseline value of the outcome. We will perform regression diagnostics and will repeat analyses using the non-parametric Wilcoxon rank-sum test if there are extreme outliers or influential observations. Groups will be compared with respect to count outcomes at 18 months (physical community integration) using graphical tools and the non-parametric Wilcoxon rank-sum test. For binary outcomes at 18 months (sustained academic and vocational

participation, and income above low income cut-off[4]), differences in proportions with 95% confidence intervals will be estimated and tested using the chi-square or Fisher's exact test. Given the small sample size of this pilot randomized trial, all results will be interpreted with caution and with the intention of generating data and hypotheses for conducting a larger trial.

All efforts will be made to reduce participants' attrition and drop-out. As mentioned previously, we believe our strong relationship with the outreach workers and community agencies will help minimize loss to follow-up (Slesnick et al., 2009). In addition, we have made it very clear in the participant information and consent form that participants in the rent subsidies plus mentorship arm may continue in the study (receiving only rent subsidies) if they are unable to continue in a mentor-mentee relationship.

5b. Qualitative Data

In keeping with the emergent, iterative nature of research using a qualitative design (Denzin & Lincoln, 2011; Eakin & Mykhalovskiy, 2003), data analysis and interpretation will begin immediately after the first qualitative data generation session (at baseline). The semi-structured individual interviews and focus groups will be audio recorded and transcribed verbatim. In order to conduct a more nuanced analysis of the data, the transcriptionist will be instructed to note short responses, uncooperative tones, and literal silence (Eakin & Mykhalovskiy; Kawabata & Gastaldo, 2015). Prior to each subsequent qualitative data generation session, members of the research team will conduct a preliminary data analysis, reading the interview transcripts multiple times, separating the data into coded segments, making analytic memos beside sections of the transcripts, identifying emerging themes (and comparing/contrasting these between respondents), and compiling new questions (Creswell, 2014; Denzin & Lincoln). Those participating in the individual interviews and the focus groups will be asked for their perspectives on the emerging interpretations at each visit and these perspectives will play a key role in helping shape the data analysis and help ensure the trustworthiness of the data (Creswell; Loiselle, Profetto-McGrath, Polit, & Tatano Beck, 2004). The web-based application Dedoose (SocioCultural Research Consultants, LLC,

[4] Based on family and community size.

2018) will be utilized to assist with sorting and coding the qualitative data.

6. Ethical Considerations

There are important ethical considerations that must be considered with any type of research. This is especially true of RCTs conducted with marginalized populations (Solomon et al., 2009). Accordingly, we have endeavored to weave ethical considerations into all aspects of the study design (Solomon et al.), including our decision to utilize a CBPAR methodology and to modify the study design so that all of the participants will receive rent subsidies. Ethical approval for this study will be obtained from the Providence St. Joseph's and St. Michael's Healthcare Research Ethics Board (REB).

6a. Benefits and Risks to Participants

All of the study participants will likely find it beneficial to receive rent subsidies. Those randomized to the intervention group may also benefit from receiving regular interactions with a mentor. Participants selected for qualitative interviews might benefit from the opportunity to share their integration-related experiences with Dr. Thulien on a regular basis. Additionally, participants may derive satisfaction from knowing that their contributions will help advance our understanding about how best to design interventions that assist formerly homeless young people to achieve meaningful social integration.

We believe theoretical justification exists for expecting that the proposed mentoring intervention is likely to produce effective outcomes; however, research ethics demands that we mitigate any potential risk to the research participants (Solomon et al., 2009). As previously mentioned, we will rely on the expertise of our community partners to screen and train the mentors. In addition, as highlighted previously, the outreach workers will work closely with the study participants and with our research team, and will alert our team if there are any concerns about a mentor-mentee relationship. These concerns will be relayed to our community partners so they can take appropriate action. The Providence St. Joseph's and St. Michael's Healthcare REB will be notified if a mentor-mentee relationship is terminated due to actions that violate the mentor-mentee code of conduct.

Participants will be assured that their participation or lack of participation in the study will not negatively impact their relationship with our community partners or their ability to access services at St. Michael's Hospital or support from other social services agencies (e.g., OW/ODSP).

6b. Privacy and Confidentiality

This mixed methods RCT will use multiple and varied data sources. This comprehensiveness is critical to the objectives of the study, yet may increase invasion of participant privacy. This privacy concern will be clearly communicated to potential participants, as will the measures for protecting security and confidentiality, prior to consent.

All of the data collected will be kept in strict confidence. While participants' names will appear on the consent forms, pseudonyms (created by the participants) will be used in place of their real names on all documents related to data generation, including the audio recordings and interview transcripts. A key that links each participant name with a pseudonym will be created and stored as a separate electronic file. All electronic data will be stored on a secure server at the Centre for Urban Health Solutions or the McMaster University School of Nursing and only accessible by select members of the research team.

The individual interviews and focus groups will be audio recorded using a password protected application on a password protected electronic device. The audio recordings from the individual interviews and focus groups will be deleted once the transcripts have been stored on the secure server and entered into Dedoose (encrypted and password-protected) (SocioCultural Research Consultants, LLC, 2018). Paper copies of the data (e.g., consent forms and standardized quantitative measures) will be stored in a locked filing cabinet at the Centre for Urban Health Solutions or the McMaster University School of Nursing – an area only accessible to those with electronic key access. All paper and electronic files will be retained for a period of up to five years from study closure.

The consent form indicates that limits to confidentiality apply if a participant discloses that they intended to hurt themselves or others, or if they inform a member of the research team that someone under the age of 16 years is suffering abuse and/or neglect.

7. Dissemination

In keeping with our CBPAR methodology, we are committed to disseminating evidence *with* our community partners to build community capacity and improve the lives of the young people participating in this study (Chenail et al., 2012; Wallerstein et al., 2018). Moreover, given our use of Critical Social Theory, we are obliged to not only present our findings, but to expose and explicate the relational processes (e.g., subjective experience of low socioeconomic position and low social class) that may be preventing formerly homeless young people from achieving meaningful social integration (Madison, 2012; Strega, 2005). With an emphasis on 'actionable' data (Chenail et al.), we anticipate disseminating our findings broadly to both academic and community-based audiences in a variety of formats ranging from scientific journal papers to oral presentations.

8. Limitations

This study has a number of limitations. First, the young people recruited for this pilot study will be a small sample of youth connected to urban-based social service providers in the province of Ontario. Thus, the findings may not be generalizable to formerly homeless young people living in other contexts and/or not connected to social service agencies. Second, the quantitative instruments are based on self-report and may involve a degree of response bias. Finally, the quantitative measures we have chosen are what we believe to be surrogate markers of meaningful social integration. Future work will likely be needed to more accurately capture this complex concept.

9. Significance

This pilot RCT study will be the first to test the impact of economic and social supports on meaningful social integration for formerly homeless young people living in market rent housing. We believe the mixed methods design will illuminate important contextual factors that must be considered if the intervention is to be scaled up and replicated elsewhere.

Importantly, the CBPAR framework will incorporate the perspectives of the community, including formerly homeless young people, who are in the best position to determine what might work best in the context of their lives.

References

Altena, A. M., Brilleslijper-Kater, S. N., & Wolf, J. R. (2010). Effective interventions for homeless youth: A systematic review. *American Journal of Preventive Medicine*, *38*(6), 637-645. doi: 10.1016/j.amepre.2010.02.017.

Beck, A. T., Weissman, A., Lester, D., & Trexler, L. (1974). The measurement of pessimism: The hopelessness scale. *Journal of Consulting and Clinical Psychology, 41*(6), 639–660. doi: 10.1037/h0037562.

Brueckner, M., Green, M., & Saggers, S. (2011). The trappings of home: Young homeless people's transitions towards independent living. *Housing Studies*, *26*(1), 1-16. doi:10.1080/02673037.2010.512751.

Chenail. R. J., St. Goerge, S., Wulff, D., & Cooper, R. (2012). Action research: The methodologies. In P. L. Munhall (Ed.), *Nursing research: A qualitative perspective* (5th ed.) (pp. 455-470). Sudbury, MS: Jones & Bartlett.

Ciarolo, J. A., Edwards, D. W., Kiresuk, T. J., Newman, F. L., & Brown, T. R. (1981). *Colorado symptom index.* Washington, DC: National Institute of Mental Health.

Coren, E., Hossain, R., Pardo Pardo, J., & Bakker, B. (2016). Interventions for promoting

reintegration and reducing harmful behaviour and lifestyles in street-connected children and young people. *Cochrane database of systematic reviews*, *2016*(1), 1-152. doi: 10.1002/14651858.CD009823.pub3.

Craig, P., Dieppe, P., Macintyre, S., Michie, S., Nazareth, I., & Petticrew, M. (2008). Developing and evaluating complex interventions: The new Medical Research Council guidance. *BMJ*, *337*(a1655), 1-6. doi: 10.1136/bmj.a1655.

Creswell, J.W. (2014). *Research design: Qualitative, quantitative, and mixed method approaches* (4th ed.). Thousand Oaks, CA: Sage.

Creswell, J.W., & Plano Clark, V.L. (2018). Designing and conducting mixed methods research (3rd ed.). Thousand Oaks, CA: Sage.

Dang, M. T., & Miller, E. (2013). Characteristics of natural mentoring relationships from the perspectives of homeless youth. *Journal of Child and Adolescent Psychiatric Nursing*, *26*(4), 246-253. doi: 10.1111/jcap.12038.

Denzin, N.K., & Lincoln, Y.S. (2011). *The SAGE handbook of qualitative research*. Thousand Oaks, CA: SAGE Publications Inc.

Eakin, J.M., & Mykhalovskiy, E. (2003). Reframing the evaluation of qualitative health research: Reflections on a review of appraisal guidelines in the health sciences. *Journal of Evaluation in Clinical Practice*, *9*(2), 187-194. doi: 10.1046/j.1365-2753.2003.00392.x.

Farquhar, M. C., Ewing, G., & Booth, S. (2011). Using mixed methods to develop and evaluate complex interventions in palliative care research. *Palliative Medicine*, *25*(8), 748-757. doi: 10.1177/0269216311417919.

Fletcher, R.H., Fletcher, S.W., & Fletcher, G.S. (2014). *Clinical epidemiology: The essentials* (5th ed.). Philadelphia, PA: Lippincott Williams & Wilkins.

Frederick, T., Chwalek, M., Hughes, J., Karabanow, J., & Kidd, S. (2014). How stable is stable? Defining and measuring housing stability. *Journal of Community Psychology, 42*(8), 964- 979. doi:10.1002/jcop.21665.

Gaetz, S. (2014). *Coming of age: Reimagining the response to youth homelessness in Canada.* Toronto, ON: The Canadian Homelessness Research Network Press. Retrieved from http://www.homelesshub.ca/comingofage

Gaetz, S., Dej, E., Richter, T., & Redman, M. (2016) *The state of homelessness in Canada*

2016. Toronto, ON: Canadian Observatory on Homelessness Press. Retrieved from http://www.homelesshub.ca/SOHC2016

Gaetz, S., O'Grady, B., Kidd, S., & Schwan, K. (2016). *Without a home: The national youth homelessness survey.* Toronto: Canadian Observatory on Homelessness Press. Retrieved from http://homelesshub.ca/sites/default/files/WithoutAHome-final.pdf

Gaetz, S., & Redman, M. (2016). *Federal investment in youth homelessness: Comparing Canada and the United States and a proposal for reinvestment. Canadian observatory on homelessness policy brief.* Toronto, ON: The Homeless Hub Press. Retrieved from http://homelesshub.ca/sites/default/files/Policy_Brief.pdf

Goodkind, J. R., Amer, S., Christian, C., Hess, J. M., Bybee, D., Isakson, B. L., ... Shantzek, C. (2017). Challenges and innovations in a community-based participatory randomized controlled trial. *Health Education & Behavior*, *44*(1), 123-130. doi: 10.1177/1090198116639243

Hammersley, M., & Atkinson, P. (2007). *Ethnography: Principles in Practice* (3rd ed.). London, UK: Routledge.

Hwang, S.W., & Burns, T. (2014). Health interventions for people who are homeless. *The Lancet, 384*(9953), 1541-1547. doi: 10.1016/S0140-6736(14)61133-8.

Israel, B.A., Schulz, A.J., Parker, E.A., Becker, A.B., Allen, III, Guzman, R.J., & Lichtenstein, R. (2018). Critical issues in developing and following CBPR principles. In N. Wallerstein, B. Duran, J. Oetzel, & M. Minkler (Eds.), *Community-based participatory research for health: Advancing social and health equity* (3rd ed.) (pp. 31-44). San Francisco, CA: Jossey-Bass.

Karabanow, J. (2008). Getting off the street: Exploring the process of young people's street exits. *American Behavioral Scientist, 51*(6)*,* 772-788. doi:10.1177/0002764207311987.

Karabanow, J., Carson, A., & Clement, P. (2010). *Leaving the streets: Stories of Canadian youth*. Halifax, NS: Fernwood Publishing.

Karabanow, J., Kidd, S., Frederick, T., & Hughes, J. (2016). Toward housing stability: Exiting homelessness as an emerging adult. *Journal of Sociology & Social Welfare, 43*(1), 121- 148. Retrieved from https://wmich.edu/socialworkjournal

Kawabata, M., & Gastaldo, D. (2015). The less said, the better: Interpreting silence in qualitative research. *International Journal of Qualitative Research Methods, 14*(4), 1-9.

doi:10.1177/1609406915618123.

Kidd, S.A., Frederick, T., Karabanow, J., Hughes, J., Naylor, T., & Barbic, S. (2016). A mixed methods study of recently homeless youth efforts to sustain housing and stability. *Child and Adolescent Social Work Journal*, *33*(3), 207-218. doi:10.1007/s10560-015-0424.

Kozloff, N., Adair, C. E., Lazgare, L. I. P., Poremski, D., Cheung, A. H., Sandu, R., & Stergiopoulos, V. (2016). "Housing first" for homeless youth with mental illness. *Pediatrics*, *138*(4), e20161514. doi: 10.1542/peds.2016-1514

Kulik, D.M., Gaetz, S., Crowe, C., & Ford-Jones, E.L. (2011). Homeless youth's overwhelming health burden: A review of the literature. *Paediatric Child Health*, *16*(6), e43-e47. doi: 10.1093/pch/16.6e43.

Kusenbach, M. (2003). Street phenomenology: The go-along as ethnographic research tool. *Ethnography, 4*(3), 455-485. doi: 10.1177/146613810343007.

Lee, R. M., & Robbins S. B. (1995). Measuring belongingness: The social connectedness and the social assurance scales. *Journal of Counseling Psychology*, *42*(2), 232-241. doi: 10.1037/0022-0167.42.2.232.

Lewin, S., Glenton, C., & Oxman, A. D. (2009). Use of qualitative methods alongside randomised controlled trials of complex healthcare interventions: methodological study. *BMJ*, *339*(b3496), 1-7. doi: 10.1136/bmj.b3496.

Loiselle, C.G., Profetto-McGrath, J., Polit, D.F., & Tatano Beck, C.T. (2004). *Canadian essentials of nursing research*. Philadelphia, PA: Lippincott Williams & Wilkins.

Luchenski, S., Maguire, N., Aldridge, R. W., Hayward, A., Story, A., Perri, P., ... Hewett, N. (2017). What works in inclusion health: overview of effective interventions for marginalised and excluded populations. *The Lancet*, *391*(10117), 266-280. doi:10.1016/S0140-6736(17)31959-1.

Madison, D.S. (2012). *Critical ethnography: Method, ethics, and performance* (2nd ed.). Thousand Oaks, CA: Sage Publication, Inc.

Mayock, P., O'Sullivan, E., & Corr, M.L. (2011). Young people exiting homelessness: An exploration of process, meaning and definition. *Housing Studies, 26*(6), 803-826. doi:10.1080/02673037.2011.593131.

McCay, E., Carter, C., Aiello, A., Quesnel, S., Langley, J., Hwang, S., ... Karabanow, J.

(2015). Dialectical Behavior Therapy as a catalyst for change in street-involved youth: A mixed methods study. *Children and Youth Services Review*, *58*, 187-199. doi: 10.1016/j.childyouth.2015.09.021.

McCay, E. & Seeman, M. (1998). A scale to measure the impact of a schizophrenic illness on an individual's self-concept. *Archives of Psychiatric Nursing, 12(1),* 41-49. doi: 10.1016/S0883-9417(98)80007-1.

Milburn, N.G., Rice, E., Rotheram-Borus, M.J., Mallett, S., Rosenthal, D., Batterham, P., ... Duan, N. (2009). Adolescents exiting homelessness over two years: The risk amplification and abatement model. *Journal of Research on Adolescence, 19*(4), 762-785. doi:10.1111/j.1532-7795.2009.00610.x.

Moore, G. F., Audrey, S., Barker, M., Bond, L., Bonell, C., Hardeman, W., ... Baird, J. (2015). Process evaluation of complex interventions: Medical Research Council guidance. *BMJ, 350*(h1258), 1-6. doi: 10.1136/bmj.h1258.

Ontario Human Rights Commission. (n.d.). *Housing as a human right.* Retrieved from http://www.ohrc.on.ca/en/right-home-report-consultation-human-rights-and-rental-housing-ontario/housing-human-right

Popay, J., Escorel, S., Hernandez, M., Johnston, H., Mathieson, J., & Rispel, L. (2008). *Understanding and tackling social exclusion: Final report to the WHO commission on social determinants of health from the social exclusion knowledge network*. Retrieved from http://www.who.int/social_determinants/themes/socialexclusion/en/

Prasad, P. (2005). *Crafting qualitative research: Working in the postpositivist traditions.* New York, NY: M.E. Sharpe.

Public Interest. (2009). *Changing patterns for street involved youth.* Toronto, ON: Author. Retrieved from http://www.worldvision.ca/Programs-and- Projects/Canadian Programs/Documents/ChangingPatternsForStreetInvolvedYouth.pdf

Quilgars, D., & Pleace, N. (2016). Housing First and Social Integration: A Realistic Aim? *Social Inclusion*, *4*(4), 5-15. doi:10.17645/si.v4i4.672.

Rosenberg, M. (1965). Society and the adolescent self-image. Princeton, NJ: Princeton University Press.

Rutman, A., Hubberstey, A., Barlow, A., & Brown, E. (2005). Supporting young people's

transitions from care: Reflections on doing participatory action research with youth from care. In L. Brown & S. Strega (Eds.), *Research as resistance: Critical, Indigenous, & anti-oppressive approaches* (pp. 153-179). Toronto, ON: Canadian Scholars' Press/Women's Press.

Slesnick, N., Dashora, P., Letcher, A., Erdem, G., & Serovich, J. (2009). A review of services and interventions for runaway and homeless youth: Moving forward. *Children and Youth Services Review*, *31*(7), 732-742. doi: 10.1016/j.childyouth.2009.01.006.

SocioCultural Research Consultants, LLC. (2018). Dedoose (Version 8.0.35) [web application]. Retrieved from http://www.dedoose.com

Solar, O., & Irwin, A. (2010). *A conceptual framework for action on the social determinants of health: Social determinants of health discussion paper 2.* Geneva, Switzerland: World Health Organization Press. Retrieved from http://www.who.int/sdhconference/resources/ConceptualframeworkforactiononSDH_eng.pdf

Solomon, P., Cavanaugh, M.M., & Draine, J. (2009). *Randomized controlled trials: Design and implementation for community-based psychosocial interventions.* New York, NY: Oxford University Press.

Stergiopoulos, V., Gozdzik, A., O'Campo, P., Holtby, A., Jeyaratnam, J., & Tsemberis, S. (2014). Housing first: Exploring participants' early support needs. *BMC Health Services Research, 14*(167), 1-15. doi:10.1186/1472-6963-14-16.

Strega, B. (2005). The view from the poststructural margins: Epistemology and methodology reconsidered. In L. Brown & S. Strega (Eds.), *Research as resistance: Critical, Indigenous, & anti-oppressive approaches* (pp. 199-235). Toronto, ON: Canadian Scholars' Press/Women's Press.

Thompson, A. E., Greeson, J. K., & Brunsink, A. M. (2016). Natural mentoring among older youth in and aging out of foster care: A systematic review. *Children and Youth Services Review*, *61*, 40-50. doi: 10.1016/j.childyouth.2015.12.006.

Thulien, N. S., Gastaldo, D., Hwang, S. W., & McCay, E. (2018). The elusive goal of social integration: A critical examination of the socio-economic and psychosocial consequences experienced by homeless young people who obtain housing. *Canadian Journal of Public Health*, *109*(1), 89-98. doi: 10.17269/s41997-018-0029-6.

Toro, P. A., Rabideau, J. M. P., Bellavia, C. W., Daeschler, C. V., Wall, D. D., Thomas, D. M., & Smith, S. J. (1997). Evaluating an intervention for homeless persons: results of a field experiment. *Journal of consulting and clinical psychology*, *65*(3), 476-484. doi: 10.1037//0022-006X.65.3.476.

Urbaniak, G.C., & Plous,S. (2013). Research Randomizer (Version 4.0) [Computer software]. Retrieved from https://www.randomizer.org

Van Dam, L., Smit, D., Wildschut, B., Branje, S. J. T., Rhodes, J. E., Assink, M., & Stams, G. J. J. M. (2018). Does natural mentoring matter? A multilevel meta-analysis on the association between natural mentoring and youth outcomes. *American Journal of Community Psychology, 0,* 1-18. doi: 10.1002/ajcp.12248.

Wallerstein, N. & Duran, B. (2006). Using community-based participatory research to address health disparities. *Health Promotion Practice, 7*(3), 312-323. doi: 10.1177/1524839906289376.

Wallerstein, N. & Duran, B. (2010). Community-based participatory research contributions to intervention research: The intersection of science and practice to improve health equity. *American Journal of Public Health, 100*(S1), S40-S46. doi: 10.2105/AJPH.2009.

Wallerstein, N., Duran, B., Oetzel, J.G., & Minkler, M. (2018). *Community-based participatory research for health: Advancing social and health equity* (3rd ed.). San Francisco, CA: Jossey-Bass.

Transitioning Youth Out of Homelessness: A Mixed Methods Community-Based Pilot Randomized Controlled Trial of a Rent Subsidy and Mentoring Intervention in Three Canadian Cities

1. Background and Rationale

Young people comprise almost 20% of the homeless population in Canada (Gaetz, Dej, Richter, & Redman, 2016). It is estimated between 35,000 and 40,000 Canadian youth (ages 13 – 25) are homeless at some point during the year and at least 6,000 on any given night (Gaetz, O'Grady, Kidd, & Schwan, 2016; Gaetz & Redman, 2016).

We know a great deal about the risk factors associated with young people entering and becoming entrenched in street life (e.g., intergenerational poverty, childhood abuse, inadequate education, and limited employment opportunities), but we know much less about how to facilitate and sustain transitions off the streets (Karabanow, 2008; Kidd et al., 2016; Kulik, Gaetz, Crowe, & Ford-Jones, 2011; Mayock, O'Sullivan, & Corr, 2011). In fact, in the peer-reviewed literature, the evidence is scarce to non-existent for rigorous interventions targeting housing outcomes, life trajectories, quality of life, and social integration[1] for young people experiencing homelessness (Altena, Brilleslijiper-Kater, & Wolf, 2010; Coren, Hossain, Pardo, & Bakker, 2016; Hwang & Burns, 2014; Luchenski et al., 2017). Understanding how to create and support successful pathways out of homelessness is crucial, because once youth become entrenched in street life, it becomes much harder for them to exit homelessness and escape a life of poverty (Gaetz, 2014; Karabanow, Carson, & Clement, 2010; Milburn et al., 2009; Public Interest, 2009).

Intuitively, it may seem that one important way to improve the life trajectories of young people experiencing homelessness is to provide them with a home. However, from the limited research that

[1] The concept of social integration is complex and often inconsistently defined and poorly measured (Quilgars & Pleace, 2016). For the purpose of this study, we drew from the literature on the social determinants of health and social exclusion, and adopted a holistic definition of social integration, incorporating both the tangible (e.g., access to education and a living wage) and intangible (e.g., sense of connection and belonging) aspects of meaningful and equitable societal participation (Luchenski et al., 2017; Popay et al., 2008; Solar & Irwin, 2010).

has been done in this area, we know that formerly homeless young people continue to experience significant challenges – particularly when it comes to mainstream social integration – even after they are 'successfully' housed (Thulien, Gastaldo, Hwang, & McCay, 2018). Moreover, these challenges seem to persist regardless of the type of housing (e.g., subsidized vs. market rent) provided (Brueckner, Green, & Saggers, 2011; Kidd et al., 2016; Kozloff et al., 2016).

Evidence from one of the most rigorous longitudinal studies with formerly homeless youth (ages 16 – 25) to date highlights that the procurement of a home does not necessarily translate into a sense of belonging or connection to mainstream society (Kidd et al., 2016). This mixed methods study of 51 formerly homeless young people living in two major urban centers in Canada showed that, despite living in stable or semi-stable accommodations (53% lived in subsidized housing), participants continued to face substantial challenges such as poverty-level incomes and limited mainstream social networks which, over the course of one year, contributed to a significant decline in hope, no gains in community integration, and a sense of being 'stuck' (Frederick, Chwalek, Hughes, Karabanow, & Kidd, 2014; Karabanow, Kidd, Frederick, & Hughes, 2016; Kidd et al.). Moreover, community integration challenges were significantly worse for participants living in independent (market rent) housing.

A sub-group analysis of 156 young people (ages 18 – 24) with mental health challenges who participated in a 24-month randomized controlled trial (RCT) of 'Housing First' (access to subsidized housing and comprehensive social service supports [e.g., treatment for mental health challenges] at home or in the community) in five Canadian cities – the largest RCT of Housing First to date – indicate similar findings of ongoing hardship despite achieving housing stability (Kozloff et al., 2016). While the young people who received the Housing First intervention achieved significantly better housing stability compared to the treatment as usual group, they did not experience any additional improvements to other outcomes such as employment, generic quality of life, and community integration relative to treatment as usual (Kozloff et al.). Notably, the same community integration scale (Stergiopoulos et al., 2014) was used in this RCT and the aforementioned Kidd et al. study, and measures both physical integration (e.g., attending a movie or community event) and psychological integration (e.g., interactions with others and feeling like one belongs).

Findings from a ten-month ethnographic study with nine formerly homeless young people (ages 18 – 24) living in Canada's largest city also support the idea that transitioning young people out of homelessness and helping them integrate into the mainstream likely requires much more than simply providing them with a home (Thulien et al., 2018). This study is believed to be the first ethnographic study to exclusively focus on the integration experiences of formerly homeless young people living in market rent housing and showed that, despite the appearance of housing stability, the participants were living a precarious existence, attributed in part to the chronic stress and exhaustion of living in poverty and to their limited knowledge about how to move forward in life (Thulien et al.). In addition, the authors note that participants underutilized transition-related social supports (e.g., food banks and employment counseling) because these supports tended to be deficit-focused (e.g., focused on what youth did not have, not on what they had achieved) and located in areas (e.g., homeless shelters) that reminded them of their old identities as homeless youth.

As previously mentioned, little evidence exists for effective interventions that target social integration for young people who have experienced homelessness. This includes evidence on the impact of mentorship. In fact, for formal mentorship programs in general, meta-analyses have only found small overall positive effect sizes (i.e., the impact of the average mentoring program in improving youth outcomes) on the psychological, emotional, behavioral, and educational functioning of participating young people (Thompson, Greeson, & Brunsink, 2016; Van Dam et al., 2018). However, there is some emerging evidence on the benefits of 'natural mentors' – generally defined as an important, encouraging, non-parental adult that exists in a youth's social network – that may be transferrable to youth who have experienced homelessness.

A systematic review of natural mentoring for youth (ages 13 – 25) transitioning out of foster care showed that the young people benefited from a supportive adult not "tasked with enforcing daily rules and addressing misbehavior" (p. 48) and that this intervention resulted in improved behavioral, psychosocial, and academic outcomes (Thompson et al.). The authors stress the importance of cultivating *inter*dependence (as opposed to independence) for young people leaving foster care and suggest that, while traditional natural mentoring relationships tend to emerge organically, they can be facilitated and supported programmatically as well (see https://vimeo.com/115837436).

A more recent meta-analysis of natural mentoring in youth (ages 13 – 24) also supports the notion that the presence of a natural mentor can positively impact young people (Van Dam et al., 2018). This meta-analysis included all young people (not just 'at-risk' youth) and found that, similar to the aforementioned systematic review, positive youth outcomes were particularly significant in the domains of social and emotional development, and academic and vocational functioning (Van Dam et al.). Moreover, the authors found that risk status (e.g., young people who were homeless or living in foster care) did *not* moderate these positive outcomes.

While almost all of the reviewed studies of at-risk youth in the meta-analysis and the systematic review were limited by their cross-sectional design, the results do hold promise for mentoring interventions that incorporate the positive characteristics of natural mentors (i.e., more of a friendship-like, 'coach', or 'cheerleader' role) for young people who have experienced homelessness. These findings are supported by a small (n = 23) qualitative study of natural mentoring relationships among homeless youth (ages 14 – 21) that suggests "natural mentors could feasibly serve as a bridge in a coordinated effort to assist youth out of homelessness" (Dang & Miller, 2013, p. 7).

From the limited research that has been done with young people transitioning away from homelessness, the emerging evidence seems to indicate that, while structural supports such as subsidized housing and social service providers are important, these things alone are insufficient to help young people integrate into mainstream society. As it currently stands, it appears as if the burden for achieving meaningful social integration is on the formerly homeless young people, who continue to be marginalized despite achieving stable or semi-stable housing (Quilgars & Pleace, 2016; Thulien et al., 2018). Connecting these young people with an adult who exhibits the relationship-based components of natural mentoring that young people value most (e.g., genuine interest in their well-being and belief in their ability to succeed, a non-judgmental attitude and a willingness to listen, the provision of advice, guidance, affirmation and encouragement) (Dang & Miller, 2013; Thompson et al., 2016; Van Dam et al., 2018) may be key to helping them move forward and integrate into the mainstream.

This intervention will provide 24 young people (ages 16 – 26) who have transitioned out of homelessness and into market rent housing within the past year with rent subsidies for 24 months. Half of the young people will also receive mentorship. We chose to focus on young people living in market rent housing because, due to limited subsidized housing options, this is the reality for most young people exiting homelessness.

Initially, we proposed to our community partners a study design where only half the young people would receive rent subsidies, with the other half receiving 'treatment as usual'; however, we abandoned this idea after our community partners challenged the ethics of not providing or delaying rent subsidies for young people living a precarious existence and desperate for immediate, tangible support to help them remain in market rent housing. Consequently, we adopted the stance that, given housing is a basic human right[2], we would offer rent subsidies to *all* of the study participants. While this does move us away from the 'gold standard' in terms of measuring the impact of rent subsidies on social integration, we believe the mixed methods longitudinal design will still yield important insights in this regard. Moreover, this design adaptation reflects our deep commitment to engage in community-based participatory action research (CBPAR) – a methodology that challenges traditional epistemological assumptions of what constitutes 'good' evidence, demands researcher humility, stresses genuine and equitable academic-community partnerships, and facilitates the undertaking of research in a way that the *community* feels is most beneficial to their members (Goodkind et al., 2017; Wallerstein & Duran, 2006; Wallerstein, Duran, Oetzel, & Minkler, 2018).

Half of the young people will be randomized to receive regular mentorship from an adult mentor, tasked with helping their mentee bridge the gap between homelessness and mainstream living. While some of these mentors will not be 'natural' in the sense that these may not be pre-existing, organically-formed relationships (see 3a. Mentorship), the mentors will incorporate the key relationship-based components of natural mentoring mentioned previously, with a strong emphasis on a strengths-based approach (i.e., focus on the young person's strengths as opposed to their

[2] "Adequate housing is essential to one's sense of dignity, safety, inclusion and ability to contribute to the fabric of our neighbourhoods and societies…without appropriate housing it is often not possible to get and keep employment, to recover from mental illness or other disabilities, to integrate into the community, to escape physical or emotional violence or to keep custody of children" (Ontario Human Rights Commission, n.d.).

limitations) and the connection of participants to larger social networks (including education and employment).

Findings from this longitudinal pilot randomized controlled trial will help address the gap in our knowledge about the impact of financial support and mentorship on meaningful social integration for young people who have experienced homelessness and are living in market rent housing.

2. Study Aim and Objectives

The overarching aim of this mixed methods study is to assess whether and how rent subsidies and mentorship influence social integration outcomes for formerly homeless young people living in market rent housing in three urban settings.

Specifically, the objectives of this study are to:

1. Determine whether rent subsidies plus mentorship results in better social integration outcomes than only receiving rent subsidies with respect to: a) community integration (psychological and physical); and b) self-esteem at our primary endpoint of 18 months.
2. Determine whether rent subsidies plus mentorship results in better social integration outcomes than only receiving rent subsidies with respect to: a) social connectedness; b) hope; and c) sustained academic and vocational participation at our secondary endpoint of 18 months.
3. Explore whether rent subsidies plus mentorship results in better social integration outcomes than only receiving rent subsidies with respect to: a) income; b) perceived housing quality; c) psychiatric symptoms; and d) sense of engulfment at our exploratory endpoint of 18 months.
4. Integrate qualitative data to facilitate a fuller understanding of the quantitative data and deepen our understanding of what the study participants (young people and mentors) found most beneficial about the intervention and how it could be improved.

3. Study Design

This study will employ a convergent mixed methods design (i.e., quantitative and qualitative data are collected concurrently, and the findings combined) embedded within a RCT and a CBPAR framework (Creswell, 2014; Creswell & Plano Clark, 2018). We believe a mixed methods RCT is appropriate given the complex explanatory pathways (i.e., social and behavioral processes that may

act independently and interdependently) of this intervention (Lewin, Glenton, & Oxman, 2009). In addition, the qualitative data will provide insights on contextual factors that may impact the external validity of our findings (Goodkind et al., 2017). Most importantly, this design provides a crucial (and underutilized) youth-informed perspective on social integration.

The study will be conducted in three Canadian cities: Toronto, Ontario (pop. 2.8 million); Hamilton, Ontario (pop. 552,000); and St. Catharines, Ontario (pop. 133,000). The design and implementation of this study is very much collaborative effort between our research team and the following community partners: a) Covenant House Toronto; b) Living Rock Ministries; and c) The RAFT (St. Catharines).

All of the study participants (n = 24) will receive rent subsidies (ranging from \$400 – \$500/month)[3] for 24 months. This study includes funding for the rent subsidies and will be paid directly to the landlords by our community partners. St. Michael's Hospital will establish a service provider agreement with each of our community partners for this purpose.

3a. Mentorship

Participants in the intervention group (n = 12) will be matched with an adult mentor recruited by one of our community partners. Each of our community partners expressed a strong desire to take the lead in the screening and recruitment of mentors as they feel they are in the best position to work with the study participants to ensure the best mentor 'fit'. Drawing on the expertise of our community partners and sharing decision-making power is aligned with CBPAR principles and highlights our commitment to collaborative, equitable partnerships in all phases of the research process (Israel et al., 2018). Moreover, working with established community resources makes practical sense; not only will this facilitate co-learning and capacity building between the research team and our community partners (Israel et al.), but delivering the mentorship intervention under 'real world' conditions will provide important insights into scalability and sustainability (Wallerstein & Duran, 2010).

[3] Given the higher cost of rent in Toronto, youth living in Toronto will receive \$500/month, while youth living in Hamilton and St. Cathatrines will receive \$400/month.

To build capacity between community partners, Covenant House Toronto will share their comprehensive *Mentor Program Guidelines* and *Mentor Orientation Handbook* (attached to this study protocol), which will act as a guide for all sites. These booklets cover information ranging from ideal mentor characteristics to mentor code of conduct. Each of our partners will designate one person currently serving in a leadership role within the organization to conduct one-on-one interviews with potential mentors and make the final decision (in conjunction with study participants) about mentor-mentee matches. In Hamilton and St. Catharines, this process will be undertaken by the executive director of each organization and, in Toronto, it will be done by the co-ordinator of their mentorship program.

As mentioned previously, our community partners are firm in their desire to control the mentorship screening and recruitment process, and will do so in a way that works best for each organization. That being said, all three organizations have agreed to the following preliminary screening process prior to meeting potential mentors:

- The mentor must show original documentation of passing a Vulnerable Sector police check within the past three months
- The mentor should ideally be at least five years older than the mentee
- The mentor must provide three references; one must be from a current employer

The mentors will be encouraged to incorporate the key relationship-based components of natural mentors previously described (e.g., a ‘coach’ or ‘cheerleader’ role) to assist with mainstream integration. To facilitate more of an organic, natural mentor-mentee relationship, the mentors will have more flexibility than a typical formal mentorship program in the types of activities they pursue with their mentees. For example, they will not be mandated to attend shelter-based social events. Instead, mentors will be encouraged to initiate activities that direct their mentees *away* from the shelter system (and their old identities as homeless youth) and toward the mainstream (e.g., meeting for coffee at a local university campus, touring a local library, or visiting the mentor’s place of employment during business hours). All of the mentors will meet monthly with their mentees for two years. In addition, the mentor will be encouraged to touch base with their mentee via phone or text message every week. If a mentor is unable to continue their role and there are at least six months left in the study, the study participant will be matched with a new mentor.

3b. Outreach Worker

Our community partners will match all participants with an outreach worker (already employed by each agency and considered 'standard of care') who will communicate regularly with the research team, help ensure the rent subsidies are being distributed appropriately, maintain an ongoing relationship with the study participants, and monitor for 'red flags' in participants matched in mentor-mentee relationships (e.g., mentee reluctant to meet with their mentor). Matching all of the study participants with a worker will also help ensure that everyone is receiving a fairly equal level of social support from our community partners, making it easier for the research team to discern whether the outcomes of interest are more likely attributable to mentorship rather than to varied levels of agency-based support. Moreover, a review of services and interventions designed to reduce "problem behaviors" (p. 733) (e.g., substance use and risky sexual practices) among street-involved and homeless young people (ages 12 – 24) found that researchers who had strong relationships with outreach workers and the community had more effective interventions and lower attrition rates than those who did not (Slesnick, Dashora, Letcher, Erdem, & Serovich, 2009).

3c. Methodology

As previously noted, this mixed methods RCT is embedded within a CBPAR framework. With the goal of reducing health inequities through knowledge and action, CBPAR can be a powerful tool for those working with marginalized populations (Chenail, St. George, Wulff, & Cooper, 2012; Rutman, Hubberstey, Barlow, & Brown, 2005; Wallerstein & Duran, 2006; Wallerstein et al., 2018). The ontological and epistemological assumptions underpinning CBPAR methodology are closely aligned with Critical Social Theory – that is, the belief that social conditions (e.g., socioeconomic contexts) perpetuate societal power imbalances and shape our version of 'truth' (Denzin & Lincoln, 2011; Prasad, 2005). For example, some may underestimate the social integration challenges faced by formerly homeless young people because they believe that everyone is afforded the same life chances. Thus, researchers operating within this paradigm have a goal of exposing and critiquing the inequitable (and often invisible) conditions that make it challenging for the marginalized to move forward (Strega, 2005).

We will draw on the following key principles of CBPAR as we generate and analyse data (Chenail et al., 2012; Rutman et al., 2005; Wallerstein & Duran, 2006; Wallerstein et al., 2018):

- Research participants are viewed as experts in their own lives
- Concerted effort to reduce/eliminate power imbalances between the researchers and the community
- Equal value placed on academic (researcher) knowledge and experiential (community agency/youth) knowledge
- Commitment to producing practical, 'actionable' data to build community capacity and improve/transform the lives of the research participants
- Duty to remain invested with the community beyond the life of the research project

3d. Participant Eligibility and Recruitment

Twenty-four young people ages 16 – 26 who have left homelessness within the past year and are living in market rent housing will be collaboratively recruited by our research team and our community partners Covenant House Toronto, Living Rock Ministries, and The RAFT (St. Catharines). We will aim to have a roughly even balance of gender and ethno-racial representation at each site.

In addition to the above age and housing **inclusion** criteria, study participants must:

- Be able to provide free and informed consent
- Be fluent in English
- Plan on staying in or nearby the community in which they were recruited (Toronto, Hamilton, or St. Catharines) for the duration of the 24-month study
- Be willing to be matched with an adult mentor who has been screened and recommended by one of our three community partners (Covenant House Toronto, Living Rock Ministries, or The RAFT) **Note:** Each study participate will be able to select their own mentor once the potential mentors have been carefully screened by our community partners (see attached *Covenant House Toronto Mentor Program: Initial Screening Application; Covenant House Toronto Mentor Program: Screening Interview;* and *Covenant House Toronto Mentor Program: Confidential Volunteer Reference Form,* which our partners in Hamilton and St. Catharines will adopt as well*).*

Young people will be **excluded** from the study if they are:

- In imminent danger of losing their housing (e.g., facing jail time or impending eviction)
- Enrolled in another study with enhanced financial and social supports

Initial introduction to the study will be done by our community partners by someone within the youths' circle of care (e.g., an outreach worker or mental health counselor). This initial introduction will be done in-person (e.g., if the young person is visiting the agency) or over-the-phone. Agency staff will be instructed to utilize the *Transitioning Youth Out of Homelessness Information and Recruitment Poster* (see attached) and *Telephone Script for Contacting Potential Participants: Community Partners* (see attached) to guide their conversation. If a young person expresses interest in participating in the study, agency staff will obtain verbal consent to provide the young person's name, e-mail address and/or cell phone number to Dr. Naomi Thulien. Dr. Thulien will then forward this information on to the appropriate research team member (e.g., research assistant or research coordinator), who will connect with the youth over the phone (see attached *Telephone Script for Contacting Potential Participants: Research Team Member*).

3e. Consent Process

Free and informed consent will be obtained verbally and in writing from all study participants. A concerted effort has been made to ensure the consent form is in plain language. Highlighted throughout the document is the fact that informed consent is an ongoing process and can be negotiated at any time.

All of the study participants will be screened for eligibility (see criteria above) and recommended for the study by one of our community partners (Covenant House Toronto, Living Rock Ministries, and The RAFT). A member of the research team will call each recruited youth on the telephone and arrange to meet them at a location most convenient for the youth. Potential participants will be given a copy of the participant information and consent form to read. This document will also be reviewed verbally to ensure that those who have low literacy levels have been given the information required to give informed consent. During this process, the research team member will assess the capacity of the potential participant to provide free and informed consent. If it is unclear whether a youth is able to provide consent, the study co-investigator (Dr. Naomi Thulien) will be contacted immediately. Dr. Thulien will arrange for a qualified member of the research team to conduct a capacity assessment. If

it is determined that a youth is not able to consent, they will be informed of this and they will be excluded from the study. The appropriate community partner will be notified as well.

3f. Allocation Procedure

If the participant meets the eligibility criteria, informed consent will be obtained, and the participant will be enrolled in the study. During this initial meeting, enrolled participants will participate in a baseline interview. There will be no unmasking of assignment prior to randomization. Following the baseline interview, participants at each of the three study sites (Toronto (n=12), Hamilton (n=6), and St. Catharine's (n=6)) will be randomized using block randomization to either the intervention (rent subsidies plus mentoring) or control (rent subsidies only) group. Randomization will be balanced by site based on random block sizes of two and four. The advantage of using block randomization is to uniformly distribute participants into treatment groups within each site (Efird, 2010). Because small block sizes may increase the risk of guessing the allocation procedure and subsequently introducing bias into the enrolment procedure, random block sizes will be used to avoid this potential selection bias (Suresh, 2011).

A unique randomization schedule will be produced for each site using SAS (SAS Institute Inc., Cary, NC, USA), with the algorithm described in Efird (2010) and will be generated by a statistician based at St. Michael's Hospital. A research coordinator based at St. Michael's Hospital and not affiliated with the study will be the only person with access to the randomization schedule. The research coordinator will prepare sealed, opaque and sequentially numbered envelopes with the randomization results of participants. After assessing for eligibility and obtaining consent of each participant, research personnel responsible for enrolling participants will open the next randomization envelope from the sequentially ordered randomization envelope file to obtain the participant's randomized group assignment. Randomization envelopes for all sites will be held in a locked cabinet at Centre for Urban Health Solutions, St. Michael's Hospital. The research coordinator enrolling participants will record the participant's group allocation into the Participant Linking Log, record their Participant ID number onto the randomization envelope, and return all opened randomization envelopes to the independent research coordinator at St. Michael's Hospital. Subsequently, the independent St. Michael's research coordinator will complete the Master Randomization Assignment List with Participant ID numbers corresponding to each envelope

number to check for consistency in participant allocation. Both the Participant Linking Log and Master Randomization Assignment List will be securely kept on St. Michael's Hospital servers.

Participants will be informed immediately if they have been allocated to the intervention or control group (Figure 1). In keeping with typical community-based RCTs with psychosocial interventions, 'blinding' in this study would not be pragmatic (e.g., social service providers and mentors will know if participants are in the 'treatment' group) after the baseline interviews and random assignments have been conducted (Solomon, Cavanaugh, & Draine, 2009).

Figure 1.
CONSORT (**CON**solidation of the **S**tandards **O**f **R**eporting **T**rials) Diagram of Ideal Flow of Participants Through the Study.

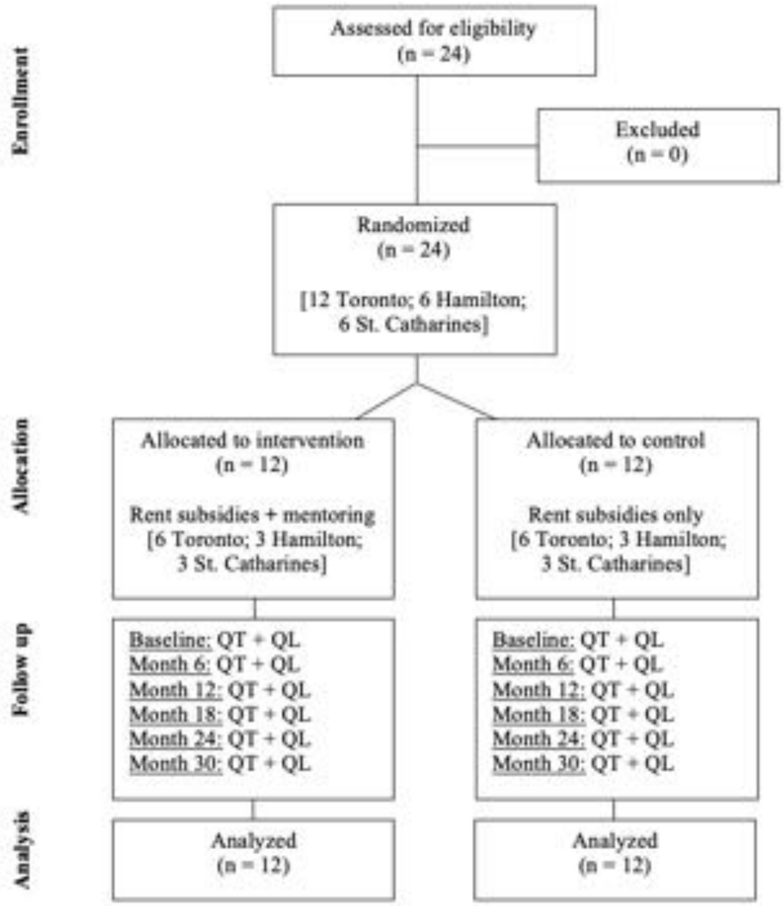

---

*__QT__ = quantitative measures (all participants). These will consist of six standardized measures to assess: community integration, social connectedness, engulfment, hope, self-esteem, and psychiatric symptoms. As well, participants will complete two brief questionnaires pertaining to: 1) education (includes skills training), employment, and income; and 2) perceived housing quality. **QL** = qualitative measures (12 participants). These will consist of one-on-one semi-structured interviews with the same 12 participants (six from each arm). The interview questions will explore issues related to feasibility and acceptability, and provide context to the quantitative responses.

4. Data Generation

Undertaking a mixed methods study where data is truly 'mixed' at the level of collection (not just at analysis) is challenging because it requires a solid understanding of the data generation requirements

of each research paradigm, and the interviews can take longer to conduct than those focusing on qualitative or quantitative methods alone (Farquhar, Ewing, & Booth, 2011). To minimize respondent burden, we have given careful consideration to the type of quantitative instruments chosen (e.g., number and length of time to complete) and will ensure that appropriate components of the qualitative interviews are prioritized (e.g., follow-up on changes in instrument scores) at each data generation session (Farquhar et al.). Additionally, all of the mixed method interviews will be conducted by the co-investigator, Dr. Naomi Thulien, who has expertise in conducting mixed method interviews with young people who have experienced homelessness.

4a. Study Outcomes

As mentioned previously, the mixed methods design of this pilot RCT reflects our desire to capture the complex independent and interdependent explanatory pathways of the intervention. This is especially crucial during this pilot stage, where we will be paying particular attention to feasibility, context, and unexpected mechanisms that produce change – factors that will influence study outcomes and provide important information regarding scalability and sustainability (Craig et al., 2008; Moore et al., 2015). To fully apprehend these complex explanatory pathways, "represent the best use of the data," and "provide an adequate assessment of the success or otherwise of an intervention that has effects across a range of domains" (Craig et al., p.3), we have aligned our key outcome variables (Table 1) with the Medical Research Council guidance on evaluating complex interventions and identified more than one primary outcome measure.

Table 1. Key Outcome Variables

**Variables**	**Instruments***
Community integration (psychological and physical)<br><br>Social connectedness<br>Engulfment	Community Integration Scale<br><br>Social Connectedness Scale – Revised<br>Modified Engulfment Scale
Self-esteem<br><br>Hope	Rosenberg Self-Esteem Scale<br><br>Beck Hopelessness Scale

Psychiatric symptoms	Modified Colorado Symptom Index
Enrollment in education (includes skills training)<br>Employment	Composite checklist
Income	Composite checklist
Perceived housing quality	Perceived Housing Quality Scale
Participant perspectives of barriers and facilitators	Individual semi-structured interviews (youth) and focus groups (mentors)<br>Composite checklist (Mentor Evaluation)

*See Table 2 for references and psychometric properties. All will be administered every six months for 30 months (except for the mentor evaluation – see Table 2).

The **primary outcome measures** for this study are: community integration (psychological and physical) and self-esteem. **Secondary outcomes** include: social connectedness, hope, and academic and vocational participation. **Exploratory outcomes** include: engulfment, psychiatric symptoms, income, perceived housing quality, and participant perspectives of intervention barriers and facilitators.

4b. Study Hypothesis

We hypothesize that, for the **primary outcome** measures of community integration and self-esteem:

1. We will observe better mean scores (community integration and self-esteem) in the participants who receive rent subsidies plus mentorship (intervention group) compared to the participants who receive rent subsidies only (control group) by our **primary endpoint of 18 months** of study participation.

We hypothesise that, for the **secondary outcome** measures of social connectedness, hope, and academic and vocational participation:

1. We will observe better mean scores (social connectedness and hope) in the intervention group relative to participants in the control group by our **secondary endpoint of 18 months** of study participation.
2. Participants in the intervention group will be more likely than the control group to demonstrate sustained engagement in academic and vocational activities (education,

employment, and/or skills training) by our **secondary endpoint of 18 months** of study participation.

4c. Quantitative Measures

Quantitative data (Appendix A – Quantitative Data Collection) will be collected at **six points** in time over the course of 30 months: baseline, month six, month 12, month 18, month 24, and month 30. **Nine instruments** (Table 2) will be employed to assess the outcome variables. We have purposely chosen instruments utilized in previous research with young people who have experienced homelessness (e.g., Kidd et al., 2016; Kozloff et al., 2016; McCay et al., 2015) so that meaningful comparisons can be made across studies (Moore et al., 2015) in this nascent area of research. One instrument we do not believe has been used with this population is the Modified Engulfment Scale (McCay & Seeman, 1998). We have included engulfment as an exploratory outcome given the emerging qualitative evidence on the crucial role of identity (self-concept) in a young person's transition away from homelessness (Brueckner et al., 2011; Karabanow et al., 2016; Thulien et al., 2018).

Table 2. Quantitative Instruments

Instrument	Psychometric Information
Beck Hopelessness Scale (Beck, Weissman, Lester, & Trexler, 1974).	This 20-item scale measures motivation, expectations, and feelings about the future (internal consistency $\alpha = .93$).
Community Integration Scale (Stergiopoulos et al., 2014).	This 11-item scale measures behavioral (e.g., participation in activities) and psychological (e.g., sense of belonging) aspects of community integration. This scale was used extensively in the Chez Soi/At Home study, but psychometric properties have yet to be reported.
Education, Employment, and Income Questionnaire	This 13-item questionnaire assesses education, employment, and income. We developed this questionnaire for the study.
Mentor Evaluation Questionnaire	This 10-item questionnaire assesses mentor effectiveness. It will be completed at month 24 by those in the intervention

	group. We developed this questionnaire for the study in collaboration with our community partners.
Modified Colorado Symptom Index (Ciarolo et al., 1981).	This 14-item scale measures the presence and frequency of psychiatric symptoms experienced in the past month. (internal consistency α = .90 – .92).
Modified Engulfment Scale (McCay & Seeman, 1998).	This 30-item scale measures the degree to which an individual's self-concept is defined by their experience of homelessness (internal consistency α = .91). We have adapted the scale for this study, substituting "experience of homelessness" for "illness".
Perceived Housing Quality (Toro et al., 1997).	This seven-item scale measures participant perception of housing choice and quality. This scale was used extensively in the Chez Soi/At Home study, but psychometric properties have yet to be reported. We have shortened it from 10 items (Chez Soi/At Home) to seven relevant items.
Rosenberg Self-Esteem Scale (Rosenberg, 1965).	This 10-item scale measures global self-worth (internal consistency α = .77 – .88).
Social Connectedness Scale – Revised (Lee & Robbins, 1995).	This 20-item scale measures belongingness – the degree to which an individual feels connected to others (internal consistency α = .92).

4d. Qualitative Measures

Qualitative measures (see Appendix B – Qualitative Data Generation) are an important feature of this study and will consist of: **1) semi-structured individual interviews** (study participants) and **2) focus groups** (mentors).

At baseline, twelve participants (six from each arm of the study) will be invited to participate in **six semi-structured individual interviews**, which will take place at the same time as the quantitative data collection: **baseline, month six, month 12, month 18, month 24, and month 30**. Participants will be purposively selected with a goal of having input from each of the three communities and a fairly equal gender and ethno-racial representation.

All of the mentors (*n* = 12) will be invited to participate in **two focus groups**, which will take place at **month 12 and month 24**.

The questions posed during the semi-structured interviews and focus groups will be guided by the study objectives, but will be conversational and exploratory in nature with particular attention to understanding *how* mentoring and/or rent subsidies influence social integration outcomes for formerly homeless young people living in market rent housing. Given the emergent nature of qualitative inquiry (Denzin & Lincoln, 2011; Eakin & Mykhalovskiy, 2003), we expect the interview and focus group questions to evolve over time as key preliminary themes begin to surface. It is anticipated that the individual interviews (including quantitative data collection, which will consistently take place first) will last approximately 60 – 75 minutes, and the focus groups approximately 60 – 90 minutes. The individual interviews and the focus groups will be conducted by Dr. Naomi Thulien at locations most convenient for those participating. To get a better sense of each young person's living situation and to minimize researcher – participant power imbalance (Israel et al., 2018), Dr. Thulien will suggest that the individual interviews take place in or nearby the young people's homes. The individual interviews and focus groups will be audio recorded and transcribed verbatim.

4e. Honoraria

All of the study participants will be paid an honorarium of $20 at each of the six quantitative data collection points. Those participating in semi-structured interviews will be paid an additional $30 at each interview. This amount was based on the co-investigator's previous experience with this population and after consulting with our community partners.

5. Data Analysis

One major critique of mixed methods RCTs is that, typically, there is no true integration (i.e., 'mixing') of quantitative and qualitative findings at the level of analysis or interpretation (Lewin, Glenton, & Oxman, 2009). Moreover, it is often unclear whether or how the quantitative and qualitative researchers have worked together to maximize the potential synergies between these different approaches (Lewin et al.). With this in mind, our study team, consisting of researchers with

quantitative and qualitative expertise, worked together to develop this study protocol and anticipate meeting quarterly to discuss the emerging analysis and to explore (and follow up on) similarities or discrepancies between the quantitative and qualitative data.

5a. Quantitative Data

All analyses will be performed using the intention-to-treat principle; that is, all participants will be included and analyzed in the groups they were originally randomized. Baseline characteristics of the intervention and control groups will be summarized using descriptive statistics (i.e., mean, standard deviation, median and interquartile range for continuous variables, and frequencies and proportions for categorical variables). We will also calculate descriptive statistics for outcomes at each study time point, and will explore differences in trajectories from baseline to 30 months follow-up between intervention and control groups using scatterplots and box-plots. Differences with 95% confidence intervals in continuous outcomes at 18 months (psychological community integration, self-esteem, social connectedness, hope, perceived housing quality, psychiatric symptoms, and sense of engulfment) between participants who received rent subsidies plus mentorship and participants who only received rent subsidies will be estimated using Analysis of Covariance (i.e., linear regression models), including an indicator of intervention group and the baseline value of the outcome. We will perform regression diagnostics and will repeat analyses using the non-parametric Wilcoxon rank-sum test if there are extreme outliers or influential observations. Groups will be compared with respect to count outcomes at 18 months (physical community integration) using graphical tools and the non-parametric Wilcoxon rank-sum test. For binary outcomes at 18 months (sustained academic and vocational participation, and income above low income cut-off[4]), differences in proportions with 95% confidence intervals will be estimated and tested using the chi-square or Fisher's exact test. Given the small sample size of this pilot randomized trial, all results will be interpreted with caution and with the intention of generating data and hypotheses for conducting a larger trial.

All efforts will be made to reduce participants' attrition and drop-out. As mentioned previously, we believe our strong relationship with the outreach workers and community agencies will help minimize loss to follow-up (Slesnick et al., 2009). In addition, we have made it very clear in the

[4] Based on family and community size.

participant information and consent form that participants in the rent subsidies plus mentorship arm may continue in the study (receiving only rent subsidies) if they are unable to continue in a mentor-mentee relationship.

5b. Qualitative Data

In keeping with the emergent, iterative nature of research using a qualitative design (Denzin & Lincoln, 2011; Eakin & Mykhalovskiy, 2003), data analysis and interpretation will begin immediately after the first qualitative data generation session (at baseline). The semi-structured individual interviews and focus groups will be audio recorded and transcribed verbatim. In order to conduct a more nuanced analysis of the data, the transcriptionist will be instructed to note short responses, uncooperative tones, and literal silence (Eakin & Mykhalovskiy; Kawabata & Gastaldo, 2015). Prior to each subsequent qualitative data generation session, members of the research team will conduct a preliminary data analysis, reading the interview transcripts multiple times, separating the data into coded segments, making analytic memos beside sections of the transcripts, identifying emerging themes (and comparing/contrasting these between respondents), and compiling new questions (Creswell, 2014; Denzin & Lincoln). Those participating in the individual interviews and the focus groups will be asked for their perspectives on the emerging interpretations at each visit and these perspectives will play a key role in helping shape the data analysis and help ensure the trustworthiness of the data (Creswell; Loiselle, Profetto-McGrath, Polit, & Tatano Beck, 2004). The web-based application Dedoose (SocioCultural Research Consultants, LLC, 2018) will be utilized to assist with sorting and coding the qualitative data.

6. Ethical Considerations

There are important ethical considerations that must be considered with any type of research. This is especially true of RCTs conducted with marginalized populations (Solomon et al., 2009). Accordingly, we have endeavored to weave ethical considerations into all aspects of the study design (Solomon et al.), including our decision to utilize a CBPAR methodology and to modify the study design so that all of the participants will receive rent subsidies. Ethical approval for this study will be obtained from the Providence St. Joseph's and St. Michael's Healthcare Research Ethics Board (REB).

6a. Benefits and Risks to Participants

All of the study participants will likely find it beneficial to receive rent subsidies. Those randomized to the intervention group may also benefit from receiving regular interactions with a mentor. Participants selected for qualitative interviews might benefit from the opportunity to share their integration-related experiences with Dr. Thulien on a regular basis. Additionally, participants may derive satisfaction from knowing that their contributions will help advance our understanding about how best to design interventions that assist formerly homeless young people to achieve meaningful social integration.

We believe theoretical justification exists for expecting that the proposed mentoring intervention is likely to produce effective outcomes; however, research ethics demands that we mitigate any potential risk to the research participants (Solomon et al., 2009). As previously mentioned, we will rely on the expertise of our community partners to screen and train the mentors. In addition, as highlighted previously, the outreach workers will work closely with the study participants and with our research team, and will alert our team if there are any concerns about a mentor-mentee relationship. These concerns will be relayed to our community partners so they can take appropriate action. The Providence St. Joseph's and St. Michael's Healthcare REB will be notified if a mentor-mentee relationship is terminated due to actions that violate the mentor-mentee code of conduct.

Participants will be assured that their participation or lack of participation in the study will not negatively impact their relationship with our community partners or their ability to access services at St. Michael's Hospital or support from other social services agencies (e.g., OW/ODSP).

6b. Privacy and Confidentiality

This mixed methods RCT will use multiple and varied data sources. This comprehensiveness is critical to the objectives of the study, yet may increase invasion of participant privacy. This privacy concern will be clearly communicated to potential participants, as will the measures for protecting security and confidentiality, prior to consent.

All of the data collected will be kept in strict confidence. While participants' names will appear on the consent forms, pseudonyms (created by the participants) will be used in place of their real names on all documents related to data generation, including the audio recordings and interview transcripts.

A key that links each participant name with a pseudonym will be created and stored as a separate electronic file. All electronic data will be stored on a secure server at the Centre for Urban Health Solutions and only be accessible by select members of the research team.

The individual interviews and focus groups will be audio recorded using a password protected application on a password protected electronic device. The audio recordings from the individual interviews and focus groups will be deleted once the transcripts have been stored on the secure server and entered into Dedoose (encrypted and password-protected) (SocioCultural Research Consultants, LLC, 2018). Paper copies of the data (e.g., consent forms and standardized quantitative measures) will be stored in a locked filing cabinet at the Centre for Urban Health Solutions – an area only accessible to those with electronic key access. All paper and electronic files will be retained for a period of up to five years from study closure.

The consent form indicates that limits to confidentiality apply if a participant discloses that they intended to hurt themselves or others, or if they inform a member of the research team that someone under the age of 16 years is suffering abuse and/or neglect.

7. Dissemination

In keeping with our CBPAR methodology, we are committed to disseminating evidence *with* our community partners to build community capacity and improve the lives of the young people participating in this study (Chenail et al., 2012; Wallerstein et al., 2018). Moreover, given our use of Critical Social Theory, we are obliged to not only present our findings, but to expose and explicate the relational processes (e.g., subjective experience of low socioeconomic position and low social class) that may be preventing formerly homeless young people from achieving meaningful social integration (Madison, 2012; Strega, 2005). With an emphasis on 'actionable' data (Chenail et al.), we anticipate disseminating our findings broadly to both academic and community-based audiences in a variety of formats ranging from scientific journal papers to oral presentations.

8. Limitations

This study has a number of limitations. First, the young people recruited for this pilot study will be a small sample of youth connected to urban-based social service providers in the province of Ontario.

Thus, the findings may not be generalizable to formerly homeless young people living in other contexts and/or not connected to social service agencies. Second, the quantitative instruments are based on self-report and may involve a degree of response bias. Finally, the quantitative measures we have chosen are what we believe to be surrogate markers of meaningful social integration. Future work will likely be needed to more accurately capture this complex concept.

9. Significance

This pilot RCT study will be the first to test the impact of economic and social supports on meaningful social integration for formerly homeless young people living in market rent housing. We believe the mixed methods design will illuminate important contextual factors that must be considered if the intervention is to be scaled up and replicated elsewhere. Importantly, the CBPAR framework will incorporate the perspectives of the community, including formerly homeless young people, who are in the best position to determine what might work best in the context of their lives.

References

Altena, A. M., Brilleslijper-Kater, S. N., & Wolf, J. R. (2010). Effective interventions for homeless youth: A systematic review. *American Journal of Preventive Medicine*, *38*(6), 637-645. doi: 10.1016/j.amepre.2010.02.017.

Beck, A. T., Weissman, A., Lester, D., & Trexler, L. (1974). The measurement of pessimism: The hopelessness scale. *Journal of Consulting and Clinical Psychology, 41*(6), 639–660. doi: 10.1037/h0037562.

Brueckner, M., Green, M., & Saggers, S. (2011). The trappings of home: Young homeless people's transitions towards independent living. *Housing Studies*, *26*(1), 1-16. doi:10.1080/02673037.2010.512751.

Chenail. R. J., St. Goerge, S., Wulff, D., & Cooper, R. (2012). Action research: The methodologies. In P. L. Munhall (Ed.), *Nursing research: A qualitative perspective* (5th ed.) (pp. 455-470). Sudbury, MS: Jones & Bartlett.

Ciarolo, J. A., Edwards, D. W., Kiresuk, T. J., Newman, F. L., & Brown, T. R. (1981). *Colorado symptom index.* Washington, DC: National Institute of Mental Health.

Coren, E., Hossain, R., Pardo Pardo, J., & Bakker, B. (2016). Interventions for promoting

reintegration and reducing harmful behaviour and lifestyles in street-connected children and young people. *Cochrane database of systematic reviews*, *2016*(1), 1-152. doi: 10.1002/14651858.CD009823.pub3.

Craig, P., Dieppe, P., Macintyre, S., Michie, S., Nazareth, I., & Petticrew, M. (2008). Developing and evaluating complex interventions: The new Medical Research Council guidance. *BMJ*, *337*(a1655), 1-6. doi: 10.1136/bmj.a1655.

Creswell, J.W. (2014). *Research design: Qualitative, quantitative, and mixed method approaches* (4th ed.). Thousand Oaks, CA: Sage.

Creswell, J.W., & Plano Clark, V.L. (2018). Designing and conducting mixed methods research (3rd ed.). Thousand Oaks, CA: Sage.

Dang, M. T., & Miller, E. (2013). Characteristics of natural mentoring relationships from the perspectives of homeless youth. *Journal of Child and Adolescent Psychiatric Nursing*, *26*(4), 246-253. doi: 10.1111/jcap.12038.

Denzin, N.K., & Lincoln, Y.S. (2011). *The SAGE handbook of qualitative research*. Thousand Oaks, CA: SAGE Publications Inc.

Eakin, J.M., & Mykhalovskiy, E. (2003). Reframing the evaluation of qualitative health research: Reflections on a review of appraisal guidelines in the health sciences. *Journal of Evaluation in Clinical Practice*, *9*(2), 187-194. doi: 10.1046/j.1365-2753.2003.00392.x.

Efird, J. (2011). Block randomization with randomly selected block sizes. *International Journal of Environmental Research and Public Health, 8*(1), 15-20. doi: 10.3390/ijerph8010015.

Farquhar, M. C., Ewing, G., & Booth, S. (2011). Using mixed methods to develop and evaluate complex interventions in palliative care research. *Palliative Medicine*, *25*(8), 748-757. doi: 10.1177/0269216311417919.

Fletcher, R.H., Fletcher, S.W., & Fletcher, G.S. (2014). *Clinical epidemiology: The essentials* (5th ed.). Philadelphia, PA: Lippincott Williams & Wilkins.

Frederick, T., Chwalek, M., Hughes, J., Karabanow, J., & Kidd, S. (2014). How stable is stable? Defining and measuring housing stability. *Journal of Community Psychology, 42*(8), 964-979. doi:10.1002/jcop.21665.

Gaetz, S. (2014). *Coming of age: Reimagining the response to youth homelessness in Canada.* Toronto, ON: The Canadian Homelessness Research Network Press. Retrieved from http://www.homelesshub.ca/comingofage

Gaetz, S., Dej, E., Richter, T., & Redman, M. (2016) *The state of homelessness in Canada 2016.* Toronto, ON: Canadian Observatory on Homelessness Press. Retrieved from http://www.homelesshub.ca/SOHC2016

Gaetz, S., O'Grady, B., Kidd, S., & Schwan, K. (2016). *Without a home: The national youth homelessness survey.* Toronto: Canadian Observatory on Homelessness Press. Retrieved from http://homelesshub.ca/sites/default/files/WithoutAHome-final.pdf

Gaetz, S., & Redman, M. (2016). *Federal investment in youth homelessness: Comparing Canada and the United States and a proposal for reinvestment. Canadian observatory on homelessness policy brief.* Toronto, ON: The Homeless Hub Press. Retrieved from http://homelesshub.ca/sites/default/files/Policy_Brief.pdf

Goodkind, J. R., Amer, S., Christian, C., Hess, J. M., Bybee, D., Isakson, B. L., ... Shantzek, C. (2017). Challenges and innovations in a community-based participatory randomized controlled trial. *Health Education & Behavior*, *44*(1), 123-130. doi: 10.1177/1090198116639243

Hammersley, M., & Atkinson, P. (2007). *Ethnography: Principles in Practice* (3rd ed.). London, UK: Routledge.

Hwang, S.W., & Burns, T. (2014). Health interventions for people who are homeless. *The Lancet, 384*(9953), 1541-1547. doi: 10.1016/S0140-6736(14)61133-8.

Israel, B.A., Schulz, A.J., Parker, E.A., Becker, A.B., Allen, III, Guzman, R.J., & Lichtenstein, R. (2018). Critical issues in developing and following CBPR principles. In N. Wallerstein, B. Duran, J. Oetzel, & M. Minkler (Eds.), *Community-based participatory research for health: Advancing social and health equity* (3rd ed.) (pp. 31-44). San Francisco, CA: Jossey-Bass.

Karabanow, J. (2008). Getting off the street: Exploring the process of young people's street exits. *American Behavioral Scientist, 51*(6), 772-788. doi:10.1177/0002764207311987.

Karabanow, J., Carson, A., & Clement, P. (2010). *Leaving the streets: Stories of Canadian youth*. Halifax, NS: Fernwood Publishing.

Karabanow, J., Kidd, S., Frederick, T., & Hughes, J. (2016). Toward housing stability: Exiting homelessness as an emerging adult. *Journal of Sociology & Social Welfare, 43*(1), 121-148. Retrieved from https://wmich.edu/socialworkjournal

Kawabata, M., & Gastaldo, D. (2015). The less said, the better: Interpreting silence in

qualitative research. *International Journal of Qualitative Research Methods, 14*(4), 1-9. doi:10.1177/1609406915618123.

Kidd, S.A., Frederick, T., Karabanow, J., Hughes, J., Naylor, T., & Barbic, S. (2016). A mixed methods study of recently homeless youth efforts to sustain housing and stability. *Child and Adolescent Social Work Journal*, *33*(3), 207-218. doi:10.1007/s10560-015-0424.

Kozloff, N., Adair, C. E., Lazgare, L. I. P., Poremski, D., Cheung, A. H., Sandu, R., & Stergiopoulos, V. (2016). "Housing first" for homeless youth with mental illness. *Pediatrics*, *138*(4), e20161514. doi: 10.1542/peds.2016-1514

Kulik, D.M., Gaetz, S., Crowe, C., & Ford-Jones, E.L. (2011). Homeless youth's overwhelming health burden: A review of the literature. *Paediatric Child Health*, *16*(6), e43-e47. doi: 10.1093/pch/16.6e43.

Kusenbach, M. (2003). Street phenomenology: The go-along as ethnographic research tool. *Ethnography, 4*(3), 455-485. doi: 10.1177/146613810343007.

Lee, R. M., & Robbins S. B. (1995). Measuring belongingness: The social connectedness and the social assurance scales. *Journal of Counseling Psychology*, *42*(2), 232-241. doi: 10.1037/0022-0167.42.2.232.

Lewin, S., Glenton, C., & Oxman, A. D. (2009). Use of qualitative methods alongside randomised controlled trials of complex healthcare interventions: methodological study. *BMJ*, *339*(b3496), 1-7. doi: 10.1136/bmj.b3496.

Loiselle, C.G., Profetto-McGrath, J., Polit, D.F., & Tatano Beck, C.T. (2004). *Canadian essentials of nursing research*. Philadelphia, PA: Lippincott Williams & Wilkins.

Luchenski, S., Maguire, N., Aldridge, R. W., Hayward, A., Story, A., Perri, P., ... Hewett, N. (2017). What works in inclusion health: overview of effective interventions for marginalised and excluded populations. *The Lancet*, *391*(10117), 266-280. doi:10.1016/ S0140-6736(17)31959-1.

Madison, D.S. (2012). *Critical ethnography: Method, ethics, and performance* (2nd ed.). Thousand Oaks, CA: Sage Publication, Inc.

Mayock, P., O'Sullivan, E., & Corr, M.L. (2011). Young people exiting homelessness: An exploration of process, meaning and definition. *Housing Studies, 26*(6), 803-826. doi:10.1080/02673037.2011.593131.

McCay, E., Carter, C., Aiello, A., Quesnel, S., Langley, J., Hwang, S., ... Karabanow, J.

(2015). Dialectical Behavior Therapy as a catalyst for change in street-involved youth: A mixed methods study. *Children and Youth Services Review*, *58*, 187-199. doi: 10.1016/j.childyouth.2015.09.021.

McCay, E. & Seeman, M. (1998). A scale to measure the impact of a schizophrenic illness on an individual's self-concept. *Archives of Psychiatric Nursing, 12(1),* 41-49. doi: 10.1016/S0883-9417(98)80007-1.

Milburn, N.G., Rice, E., Rotheram-Borus, M.J., Mallett, S., Rosenthal, D., Batterham, P., ... Duan, N. (2009). Adolescents exiting homelessness over two years: The risk amplification and abatement model. *Journal of Research on Adolescence, 19*(4), 762- 785. doi:10.1111/j.1532-7795.2009.00610.x.

Moore, G. F., Audrey, S., Barker, M., Bond, L., Bonell, C., Hardeman, W., ... Baird, J. (2015). Process evaluation of complex interventions: Medical Research Council guidance. *BMJ, 350*(h1258), 1-6. doi: 10.1136/bmj.h1258.

Ontario Human Rights Commission. (n.d.). *Housing as a human right.* Retrieved from http://www.ohrc.on.ca/en/right-home-report-consultation-human-rights-and-rental-housing-ontario/housing-human-right

Popay, J., Escorel, S., Hernandez, M., Johnston, H., Mathieson, J., & Rispel, L. (2008). *Understanding and tackling social exclusion: Final report to the WHO commission on social determinants of health from the social exclusion knowledge network*. Retrieved from http://www.who.int/social_determinants/themes/socialexclusion/en/

Prasad, P. (2005). *Crafting qualitative research: Working in the postpositivist traditions.* New York, NY: M.E. Sharpe.

Public Interest. (2009). *Changing patterns for street involved youth.* Toronto, ON: Author. Retrieved from http://www.worldvision.ca/Programs-and- Projects/Canadian Programs/Documents/ChangingPatternsForStreetInvolvedYouth.pdf

Quilgars, D., & Pleace, N. (2016). Housing First and Social Integration: A Realistic Aim? *Social Inclusion*, *4*(4), 5-15. doi:10.17645/si.v4i4.672.

Rosenberg, M. (1965). Society and the adolescent self-image. Princeton, NJ: Princeton University Press.

Rutman, A., Hubberstey, A., Barlow, A., & Brown, E. (2005). Supporting young people's

transitions from care: Reflections on doing participatory action research with youth from care. In L. Brown & S. Strega (Eds.), *Research as resistance: Critical, Indigenous, & anti-oppressive approaches* (pp. 153-179). Toronto, ON: Canadian Scholars' Press/Women's Press.

Slesnick, N., Dashora, P., Letcher, A., Erdem, G., & Serovich, J. (2009). A review of services and interventions for runaway and homeless youth: Moving forward. *Children and Youth Services Review*, *31*(7), 732-742. doi: 10.1016/j.childyouth.2009.01.006.

SocioCultural Research Consultants, LLC. (2018). Dedoose (Version 8.0.35) [web application]. Retrieved from http://www.dedoose.com

Solar, O., & Irwin, A. (2010). *A conceptual framework for action on the social determinants of health: Social determinants of health discussion paper 2.* Geneva, Switzerland: World Health Organization Press. Retrieved from http://www.who.int/sdhconference/resources/ConceptualframeworkforactiononS DH_eng.pdf

Solomon, P., Cavanaugh, M.M., & Draine, J. (2009). *Randomized controlled trials: Design and implementation for community-based psychosocial interventions.* New York, NY: Oxford University Press.

Stergiopoulos, V., Gozdzik, A., O'Campo, P., Holtby, A., Jeyaratnam, J., & Tsemberis, S. (2014). Housing first: Exploring participants' early support needs. *BMC Health Services Research, 14*(167), 1-15. doi:10.1186/1472-6963-14-16.

Strega, B. (2005). The view from the poststructural margins: Epistemology and methodology reconsidered. In L. Brown & S. Strega (Eds.), *Research as resistance: Critical, Indigenous, & anti-oppressive approaches* (pp. 199-235). Toronto, ON: Canadian Scholars' Press/Women's Press.

Suresh, K. P. (2011). An overview of randomization techniques: an unbiased assessment of outcome in clinical research. *Journal of human reproductive sciences*, *4*(1), 8-11. doi: 10.4103/0974-1208.82352.

Thompson, A. E., Greeson, J. K., & Brunsink, A. M. (2016). Natural mentoring among older youth in and aging out of foster care: A systematic review. *Children and Youth Services Review*, *61*, 40-50. doi: 10.1016/j.childyouth.2015.12.006.

Thulien, N. S., Gastaldo, D., Hwang, S. W., & McCay, E. (2018). The elusive goal of social

integration: A critical examination of the socio-economic and psychosocial consequences experienced by homeless young people who obtain housing. *Canadian Journal of Public Health*, *109*(1), 89-98. doi: 10.17269/s41997-018-0029-6.

Toro, P. A., Rabideau, J. M. P., Bellavia, C. W., Daeschler, C. V., Wall, D. D., Thomas, D. M., & Smith, S. J. (1997). Evaluating an intervention for homeless persons: results of a field experiment. *Journal of consulting and clinical psychology*, *65*(3), 476-484. doi: 10.1037//0022-006X.65.3.476.

Van Dam, L., Smit, D., Wildschut, B., Branje, S. J. T., Rhodes, J. E., Assink, M., & Stams, G. J. J. M. (2018). Does natural mentoring matter? A multilevel meta-analysis on the association between natural mentoring and youth outcomes. *American Journal of Community Psychology, 0,* 1-18. doi: 10.1002/ajcp.12248.

Wallerstein, N. & Duran, B. (2006). Using community-based participatory research to address health disparities. *Health Promotion Practice, 7*(3), 312-323. doi: 10.1177/1524839906289376.

Wallerstein, N. & Duran, B. (2010). Community-based participatory research contributions to intervention research: The intersection of science and practice to improve health equity. *American Journal of Public Health, 100*(S1), S40-S46. doi: 10.2105/AJPH.2009.

Wallerstein, N., Duran, B., Oetzel, J.G., & Minkler, M. (2018). *Community-based participatory research for health: Advancing social and health equity* (3rd ed.). San Francisco, CA: Jossey-Bass.

Transitioning Youth Out of Homelessness: A Mixed Methods Community-Based Pilot Randomized Controlled Trial of a Rent Subsidy and Mentoring Intervention in Three Canadian Cities

1. Background and Rationale

Young people comprise almost 20% of the homeless population in Canada (Gaetz, Dej, Richter, & Redman, 2016). It is estimated between 35,000 and 40,000 Canadian youth (ages 13 – 25) are homeless at some point during the year and at least 6,000 on any given night (Gaetz, O'Grady, Kidd, & Schwan, 2016; Gaetz & Redman, 2016).

We know a great deal about the risk factors associated with young people entering and becoming entrenched in street life (e.g., intergenerational poverty, childhood abuse, inadequate education, and limited employment opportunities), but we know much less about how to facilitate and sustain transitions off the streets (Karabanow, 2008; Kidd et al., 2016; Kulik, Gaetz, Crowe, & Ford-Jones, 2011; Mayock, O'Sullivan, & Corr, 2011). In fact, in the peer-reviewed literature, the evidence is scarce to non-existent for rigorous interventions targeting housing outcomes, life trajectories, quality of life, and social integration[1] for young people experiencing homelessness (Altena, Brilleslijiper-Kater, & Wolf, 2010; Coren, Hossain, Pardo, & Bakker, 2016; Hwang & Burns, 2014; Luchenski et al., 2017). Understanding how to create and support successful pathways out of homelessness is crucial, because once youth become entrenched in street life, it becomes much harder for them to exit homelessness and escape a life of poverty (Gaetz, 2014; Karabanow, Carson, & Clement, 2010; Milburn et al., 2009; Public Interest, 2009).

Intuitively, it may seem that one important way to improve the life trajectories of young people experiencing homelessness is to provide them with a home. However, from the limited research that has been done in this area, we know that formerly homeless young people continue to experience significant challenges – particularly when it comes to mainstream social integration – even after they are 'successfully' housed (Thulien, Gastaldo, Hwang, & McCay, 2018). Moreover, these challenges seem to persist regardless of the type of housing (e.g., subsidized vs. market rent) provided (Brueckner, Green, & Saggers, 2011; Kidd et al., 2016; Kozloff et al., 2016).

[1] The concept of social integration is complex and often inconsistently defined and poorly measured (Quilgars & Pleace, 2016). For the purpose of this study, we drew from the literature on the social determinants of health and social exclusion, and adopted a holistic definition of social integration, incorporating both the tangible (e.g., access to education and a living wage) and intangible (e.g., sense of connection and belonging) aspects of meaningful and equitable societal participation (Luchenski et al., 2017; Popay et al., 2008; Solar & Irwin, 2010).

Evidence from one of the most rigorous longitudinal studies with formerly homeless youth (ages 16 – 25) to date highlights that the procurement of a home does not necessarily translate into a sense of belonging or connection to mainstream society (Kidd et al., 2016). This mixed methods study of 51 formerly homeless young people living in two major urban centers in Canada showed that, despite living in stable or semi-stable accommodations (53% lived in subsidized housing), participants continued to face substantial challenges such as poverty-level incomes and limited mainstream social networks which, over the course of one year, contributed to a significant decline in hope, no gains in community integration, and a sense of being 'stuck' (Frederick, Chwalek, Hughes, Karabanow, & Kidd, 2014; Karabanow, Kidd, Frederick, & Hughes, 2016; Kidd et al.). Moreover, community integration challenges were significantly worse for participants living in independent (market rent) housing.

A sub-group analysis of 156 young people (ages 18 – 24) with mental health challenges who participated in a 24-month randomized controlled trial (RCT) of 'Housing First' (access to subsidized housing and comprehensive social service supports [e.g., treatment for mental health challenges] at home or in the community) in five Canadian cities – the largest RCT of Housing First to date – indicate similar findings of ongoing hardship despite achieving housing stability (Kozloff et al., 2016). While the young people who received the Housing First intervention achieved significantly better housing stability compared to the treatment as usual group, they did not experience any additional improvements to other outcomes such as employment, generic quality of life, and community integration relative to treatment as usual (Kozloff et al.). Notably, the same community integration scale (Stergiopoulos et al., 2014) was used in this RCT and the aforementioned Kidd et al. study, and measures both physical integration (e.g., attending a movie or community event) and psychological integration (e.g., interactions with others and feeling like one belongs).

Findings from a ten-month ethnographic study with nine formerly homeless young people (ages 18 – 24) living in Canada's largest city also support the idea that transitioning young people out of homelessness and helping them integrate into the mainstream likely requires much more than simply providing them with a home (Thulien et al., 2018). This study is believed to be the first ethnographic study to exclusively focus on the integration experiences of formerly homeless young people living in market rent housing and showed that, despite the appearance of housing stability, the participants were living a precarious existence, attributed in part to the chronic stress and exhaustion of living in poverty and to their limited knowledge about how to move forward in life (Thulien et al.). In addition, the authors note that participants underutilized transition-related social supports (e.g., food banks and employment counseling) because these supports tended to be

deficit-focused (e.g., focused on what youth did not have, not on what they had achieved) and located in areas (e.g., homeless shelters) that reminded them of their old identities as homeless youth.

As previously mentioned, little evidence exists for effective interventions that target social integration for young people who have experienced homelessness. This includes evidence on the impact of mentorship. In fact, for formal mentorship programs in general, meta-analyses have only found small overall positive effect sizes (i.e., the impact of the average mentoring program in improving youth outcomes) on the psychological, emotional, behavioral, and educational functioning of participating young people (Thompson, Greeson, & Brunsink, 2016; Van Dam et al., 2018). However, there is some emerging evidence on the benefits of 'natural mentors' – generally defined as an important, encouraging, non-parental adult that exists in a youth's social network – that may be transferrable to youth who have experienced homelessness.

A systematic review of natural mentoring for youth (ages 13 – 25) transitioning out of foster care showed that the young people benefited from a supportive adult not "tasked with enforcing daily rules and addressing misbehavior" (p. 48) and that this intervention resulted in improved behavioral, psychosocial, and academic outcomes (Thompson et al.). The authors stress the importance of cultivating *inter*dependence (as opposed to independence) for young people leaving foster care and suggest that, while traditional natural mentoring relationships tend to emerge organically, they can be facilitated and supported programmatically as well (see https://vimeo.com/115837436).

A more recent meta-analysis of natural mentoring in youth (ages 13 – 24) also supports the notion that the presence of a natural mentor can positively impact young people (Van Dam et al., 2018). This meta-analysis included all young people (not just 'at-risk' youth) and found that, similar to the aforementioned systematic review, positive youth outcomes were particularly significant in the domains of social and emotional development, and academic and vocational functioning (Van Dam et al.). Moreover, the authors found that risk status (e.g., young people who were homeless or living in foster care) did *not* moderate these positive outcomes.

While almost all of the reviewed studies of at-risk youth in the meta-analysis and the systematic review were limited by their cross-sectional design, the results do hold promise for mentoring interventions that incorporate the positive characteristics of natural mentors (i.e., more of a friendship-like, 'coach', or 'cheerleader' role) for young people who have experienced homelessness. These findings are supported by a small (n = 23) qualitative study of natural mentoring relationships among homeless youth (ages 14 – 21) that

suggests “natural mentors could feasibly serve as a bridge in a coordinated effort to assist youth out of homelessness” (Dang & Miller, 2013, p. 7).

From the limited research that has been done with young people transitioning away from homelessness, the emerging evidence seems to indicate that, while structural supports such as subsidized housing and social service providers are important, these things alone are insufficient to help young people integrate into mainstream society. As it currently stands, it appears as if the burden for achieving meaningful social integration is on the formerly homeless young people, who continue to be marginalized despite achieving stable or semi-stable housing (Quilgars & Pleace, 2016; Thulien et al., 2018). Connecting these young people with an adult who exhibits the relationship-based components of natural mentoring that young people value most (e.g., genuine interest in their well-being and belief in their ability to succeed, a non-judgmental attitude and a willingness to listen, the provision of advice, guidance, affirmation and encouragement) (Dang & Miller, 2013; Thompson et al., 2016; Van Dam et al., 2018) may be key to helping them move forward and integrate into the mainstream.

This intervention will provide 24 young people (ages 16 – 26) who have transitioned out of homelessness and into market rent housing within the past year with rent subsidies for 24 months. Half of the young people will also receive mentorship. We chose to focus on young people living in market rent housing because, due to limited subsidized housing options, this is the reality for most young people exiting homelessness.

Initially, we proposed to our community partners a study design where only half the young people would receive rent subsidies, with the other half receiving ‘treatment as usual’; however, we abandoned this idea after our community partners challenged the ethics of not providing or delaying rent subsidies for young people living a precarious existence and desperate for immediate, tangible support to help them remain in market rent housing. Consequently, we adopted the stance that, given housing is a basic human right[2], we would offer rent subsidies to *all* of the study participants. While this does move us away from the ‘gold standard’ in terms of measuring the impact of rent subsidies on social integration, we believe the mixed methods longitudinal design will still yield important insights in this regard. Moreover, this design adaptation reflects our deep commitment to engage in community-based participatory action research (CBPAR) – a

[2] “Adequate housing is essential to one’s sense of dignity, safety, inclusion and ability to contribute to the fabric of our neighbourhoods and societies…without appropriate housing it is often not possible to get and keep employment, to recover from mental illness or other disabilities, to integrate into the community, to escape physical or emotional violence or to keep custody of children” (Ontario Human Rights Commission, n.d.).

methodology that challenges traditional epistemological assumptions of what constitutes 'good' evidence, demands researcher humility, stresses genuine and equitable academic-community partnerships, and facilitates the undertaking of research in a way that the *community* feels is most beneficial to their members (Goodkind et al., 2017; Wallerstein & Duran, 2006; Wallerstein, Duran, Oetzel, & Minkler, 2018).

Half of the young people will be randomized to receive regular mentorship from an adult mentor, tasked with helping their mentee bridge the gap between homelessness and mainstream living. While some of these mentors will not be 'natural' in the sense that these may not be pre-existing, organically-formed relationships (see 3a. Mentorship), the mentors will incorporate the key relationship-based components of natural mentoring mentioned previously, with a strong emphasis on a strengths-based approach (i.e., focus on the young person's strengths as opposed to their limitations) and the connection of participants to larger social networks (including education and employment).

Findings from this longitudinal pilot randomized controlled trial will help address the gap in our knowledge about the impact of financial support and mentorship on meaningful social integration for young people who have experienced homelessness and are living in market rent housing.

2. Study Aim and Objectives

The overarching aim of this mixed methods study is to assess whether and how rent subsidies and mentorship influence social integration outcomes for formerly homeless young people living in market rent housing in three urban settings.

Specifically, the objectives of this study are to:

1. Determine whether rent subsidies plus mentorship results in better social integration outcomes than only receiving rent subsidies with respect to: a) community integration (psychological and physical); and b) self-esteem at our primary endpoint of 18 months.
2. Determine whether rent subsidies plus mentorship results in better social integration outcomes than only receiving rent subsidies with respect to: a) social connectedness; b) hope; and c) sustained academic and vocational participation at our secondary endpoint of 18 months.
3. Explore whether rent subsidies plus mentorship results in better social integration outcomes than only receiving rent subsidies with respect to: a) income; b) perceived housing quality; c) psychiatric symptoms; and d) sense of engulfment at our exploratory endpoint of 18 months.

4. Integrate qualitative data to facilitate a fuller understanding of the quantitative data and deepen our understanding of what the study participants (young people and mentors) found most beneficial about the intervention and how it could be improved.

3. Study Design

This study will employ a convergent mixed methods design (i.e., quantitative and qualitative data are collected concurrently, and the findings combined) embedded within a RCT and a CBPAR framework (Creswell, 2014; Creswell & Plano Clark, 2018). We believe a mixed methods RCT is appropriate given the complex explanatory pathways (i.e., social and behavioral processes that may act independently and interdependently) of this intervention (Lewin, Glenton, & Oxman, 2009). In addition, the qualitative data will provide insights on contextual factors that may impact the external validity of our findings (Goodkind et al., 2017). Most importantly, this design provides a crucial (and underutilized) youth-informed perspective on social integration.

The study will be conducted in three Canadian cities: Toronto, Ontario (pop. 2.8 million); Hamilton, Ontario (pop. 552,000); and St. Catharines, Ontario (pop. 133,000). The design and implementation of this study is very much collaborative effort between our research team and the following community partners: a) Covenant House Toronto; b) Living Rock Ministries; and c) The RAFT (St. Catharines).

All of the study participants (n = 24) will receive rent subsidies (ranging from \$400 – \$500/month)[3] for 24 months. This study includes funding for the rent subsidies and will be paid directly to the landlords by our community partners. St. Michael's Hospital will establish a service provider agreement with each of our community partners for this purpose.

3a. Mentorship

Participants in the intervention group (n = 12) will be matched with an adult mentor recruited by one of our community partners. Each of our community partners expressed a strong desire to take the lead in the screening and recruitment of mentors as they feel they are in the best position to work with the study participants to ensure the best mentor 'fit'. Drawing on the expertise of our community partners and sharing decision-making power is aligned with CBPAR principles and highlights our commitment to collaborative, equitable partnerships in all phases of the research process (Israel et al., 2018). Moreover, working with established community resources makes practical sense; not only will this facilitate co-learning and capacity

[3] Given the higher cost of rent in Toronto, youth living in Toronto will receive \$500/month, while youth living in Hamilton and St. Cathatrines will receive \$400/month.

building between the research team and our community partners (Israel et al.), but delivering the mentorship intervention under 'real world' conditions will provide important insights into scalability and sustainability (Wallerstein & Duran, 2010).

To build capacity between community partners, Covenant House Toronto will share their comprehensive *Mentor Program Guidelines* and *Mentor Orientation Handbook* (attached to this study protocol), which will act as a guide for all sites. These booklets cover information ranging from ideal mentor characteristics to mentor code of conduct. Each of our partners will designate one person currently serving in a leadership role within the organization to conduct one-on-one interviews with potential mentors and make the final decision (in conjunction with study participants) about mentor-mentee matches. In Hamilton and St. Catharines, this process will be undertaken by the executive director of each organization and, in Toronto, it will be done by the co-ordinator of their mentorship program.

As mentioned previously, our community partners are firm in their desire to control the mentorship screening and recruitment process, and will do so in a way that works best for each organization. That being said, all three organizations have agreed to the following preliminary screening process prior to meeting potential mentors:

- The mentor must show original documentation of passing a Vulnerable Sector police check within the past three months
- The mentor should ideally be at least five years older than the mentee
- The mentor must provide three references; one must be from a current employer

The mentors will be encouraged to incorporate the key relationship-based components of natural mentors previously described (e.g., a 'coach' or 'cheerleader' role) to assist with mainstream integration. To facilitate more of an organic, natural mentor-mentee relationship, the mentors will have more flexibility than a typical formal mentorship program in the types of activities they pursue with their mentees. For example, they will not be mandated to attend shelter-based social events. Instead, mentors will be encouraged to initiate activities that direct their mentees *away* from the shelter system (and their old identities as homeless youth) and toward the mainstream (e.g., meeting for coffee at a local university campus, touring a local library, or visiting the mentor's place of employment during business hours). All of the mentors will meet monthly with their mentees for two years. In addition, the mentor will be encouraged to touch base with their mentee via phone or text message every week. If a mentor is unable to continue their role and there are at least six months left in the study, the study participant will be matched with a new mentor.

3b. Outreach Worker

Our community partners will match all participants with an outreach worker (already employed by each agency and considered 'standard of care') who will communicate regularly with the research team, help ensure the rent subsidies are being distributed appropriately, maintain an ongoing relationship with the study participants, and monitor for 'red flags' in participants matched in mentor-mentee relationships (e.g., mentee reluctant to meet with their mentor). Matching all of the study participants with a worker will also help ensure that everyone is receiving a fairly equal level of social support from our community partners, making it easier for the research team to discern whether the outcomes of interest are more likely attributable to mentorship rather than to varied levels of agency-based support. Moreover, a review of services and interventions designed to reduce "problem behaviors" (p. 733) (e.g., substance use and risky sexual practices) among street-involved and homeless young people (ages 12 – 24) found that researchers who had strong relationships with outreach workers and the community had more effective interventions and lower attrition rates than those who did not (Slesnick, Dashora, Letcher, Erdem, & Serovich, 2009).

3c. Methodology

As previously noted, this mixed methods RCT is embedded within a CBPAR framework. With the goal of reducing health inequities through knowledge and action, CBPAR can be a powerful tool for those working with marginalized populations (Chenail, St. George, Wulff, & Cooper, 2012; Rutman, Hubberstey, Barlow, & Brown, 2005; Wallerstein & Duran, 2006; Wallerstein et al., 2018). The ontological and epistemological assumptions underpinning CBPAR methodology are closely aligned with Critical Social Theory – that is, the belief that social conditions (e.g., socioeconomic contexts) perpetuate societal power imbalances and shape our version of 'truth' (Denzin & Lincoln, 2011; Prasad, 2005). For example, some may underestimate the social integration challenges faced by formerly homeless young people because they believe that everyone is afforded the same life chances. Thus, researchers operating within this paradigm have a goal of exposing and critiquing the inequitable (and often invisible) conditions that make it challenging for the marginalized to move forward (Strega, 2005).

We will draw on the following key principles of CBPAR as we generate and analyse data (Chenail et al., 2012; Rutman et al., 2005; Wallerstein & Duran, 2006; Wallerstein et al., 2018):

- Research participants are viewed as experts in their own lives
- Concerted effort to reduce/eliminate power imbalances between the researchers and the community
- Equal value placed on academic (researcher) knowledge and experiential (community agency/youth) knowledge
- Commitment to producing practical, 'actionable' data to build community capacity and improve/transform the lives of the research participants

- Duty to remain invested with the community beyond the life of the research project

3d. Participant Eligibility and Recruitment

Twenty-four young people ages 16 – 26 who have left homelessness within the past year and are living in market rent housing will be collaboratively recruited by our research team and our community partners Covenant House Toronto, Living Rock Ministries, and The RAFT (St. Catharines). We will aim to have a roughly even balance of gender and ethno-racial representation at each site.

In addition to the above age and housing **inclusion** criteria, study participants must:

- Be able to provide free and informed consent
- Be fluent in English
- Plan on staying in or nearby the community in which they were recruited (Toronto, Hamilton, or St. Catharines) for the duration of the 24-month study
- Be willing to be matched with an adult mentor who has been screened and recommended by one of our three community partners (Covenant House Toronto, Living Rock Ministries, or The RAFT) **Note:** Each study participate will be able to select their own mentor once the potential mentors have been carefully screened by our community partners (see attached *Covenant House Toronto Mentor Program: Initial Screening Application; Covenant House Toronto Mentor Program: Screening Interview;* and *Covenant House Toronto Mentor Program: Confidential Volunteer Reference Form,* which our partners in Hamilton and St. Catharines will adopt as well*).*

Young people will be **excluded** from the study if they are:

- In imminent danger of losing their housing (e.g., facing jail time or impending eviction)
- Enrolled in another study with enhanced financial and social supports

Initial introduction to the study will be done by our community partners by someone within the youths' circle of care (e.g., an outreach worker or mental health counselor). This initial introduction will be done in-person (e.g., if the young person is visiting the agency) or over-the-phone. Agency staff will be instructed to utilize the *Transitioning Youth Out of Homelessness Information and Recruitment Poster* (see attached) and *Telephone Script for Contacting Potential Participants: Community Partners* (see attached) to guide their conversation. If a young person expresses interest in participating in the study, agency staff will obtain verbal consent to provide the young person's name, e-mail address and/or cell phone number to Dr. Naomi Thulien. Dr. Thulien will then forward this information on to the appropriate research team member (e.g., research assistant or research coordinator), who will connect with the youth over the phone (see attached *Telephone Script for Contacting Potential Participants: Research Team Member*).

3e. Consent Process

Free and informed consent will be obtained verbally and in writing from all study participants. A concerted effort has been made to ensure the consent form is in plain language. Highlighted throughout the document is the fact that informed consent is an ongoing process and can be negotiated at any time.

All of the study participants will be screened for eligibility (see criteria above) and recommended for the study by one of our community partners (Covenant House Toronto, Living Rock Ministries, and The RAFT). A member of the research team will call each recruited youth on the telephone and arrange to meet them at a location most convenient for the youth. Potential participants will be given a copy of the participant information and consent form to read. This document will also be reviewed verbally to ensure that those who have low literacy levels have been given the information required to give informed consent. During this process, the research team member will assess the capacity of the potential participant to provide free and informed consent. If it is unclear whether a youth is able to provide consent, the study co-investigator (Dr. Naomi Thulien) will be contacted immediately. Dr. Thulien will arrange for a qualified member of the research team to conduct a capacity assessment. If it is determined that a youth is not able to consent, they will be informed of this and they will be excluded from the study. The appropriate community partner will be notified as well.

3f. Allocation Procedure

If the participant meets the eligibility criteria, informed consent will be obtained, and the participant will be enrolled in the study. During this initial meeting, enrolled participants will participate in a baseline interview. There will be no unmasking of assignment prior to randomization. Following the baseline interview, participants at each of the three study sites (Toronto (n=12), Hamilton (n=6), and St. Catharine's (n=6)) will be randomized using block randomization to either the intervention (rent subsidies plus mentoring) or control (rent subsidies only) group. Randomization will be balanced by site based on random block sizes of two and four. The advantage of using block randomization is to uniformly distribute participants into treatment groups within each site (Efird, 2010). Because small block sizes may increase the risk of guessing the allocation procedure and subsequently introducing bias into the enrolment procedure, random block sizes will be used to avoid this potential selection bias (Suresh, 2011).

A unique randomization schedule will be produced for each site using SAS (SAS Institute Inc., Cary, NC, USA), with the algorithm described in Efird (2010) and will be generated by a statistician based at St. Michael's Hospital. A research coordinator based at St. Michael's Hospital and not affiliated with the study will be the only person with access to the randomization schedule. The research coordinator will prepare sealed, opaque and sequentially numbered envelopes with the randomization results of participants. After assessing for eligibility and obtaining consent of each participant, research personnel responsible for enrolling participants will open the next randomization envelope from the sequentially ordered randomization envelope file to obtain the participant's randomized group assignment. Randomization envelopes for all sites will be held in a locked cabinet at Centre for Urban Health Solutions, St. Michael's Hospital. The research coordinator enrolling participants will record the participant's group allocation into the Participant Linking Log, record their Participant ID number onto the randomization envelope, and return all opened randomization envelopes to the independent research coordinator at St. Michael's Hospital. Subsequently, the independent St. Michael's research coordinator will complete the Master Randomization Assignment List with Participant ID numbers corresponding to each envelope number to check for consistency in participant allocation. Both the Participant Linking Log and Master Randomization Assignment List will be securely kept on St. Michael's Hospital servers.

Participants will be informed immediately if they have been allocated to the intervention or control group (Figure 1). In keeping with typical community-based RCTs with psychosocial interventions, 'blinding' in this study would not be pragmatic (e.g., social service providers and mentors will know if participants are in the 'treatment' group) after the baseline interviews and random assignments have been conducted (Solomon, Cavanaugh, & Draine, 2009).

Figure 1.
CONSORT (**CON**solidation of the **S**tandards **O**f **R**eporting **T**rials) Diagram of Ideal Flow of Participants Through the Study.

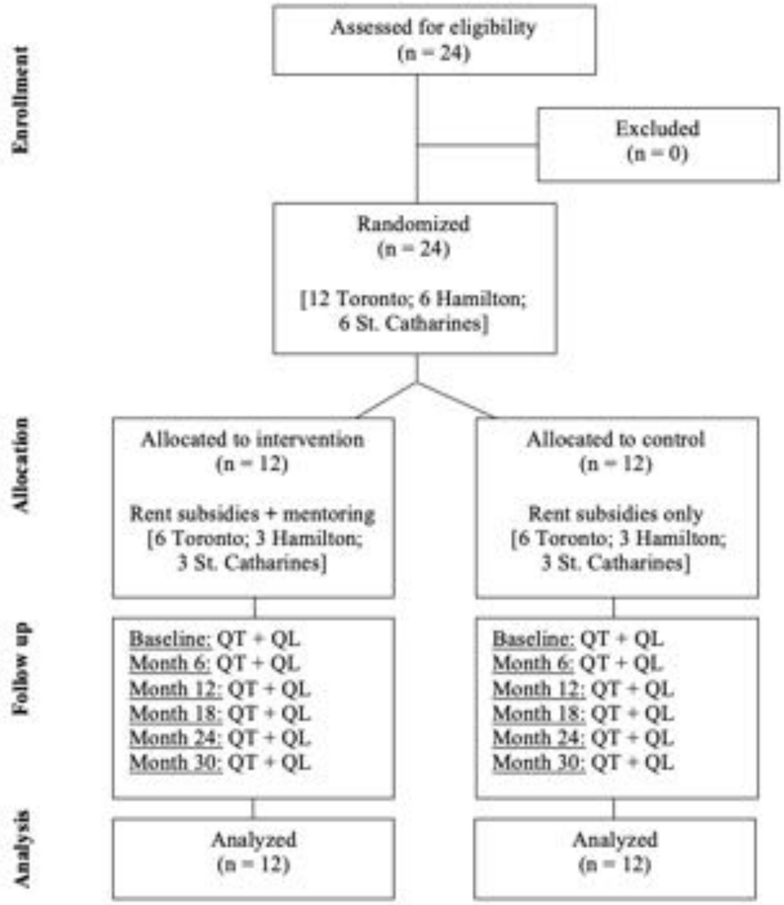

*__QT__ = quantitative measures (all participants). These will consist of six standardized measures to assess: community integration, social connectedness, engulfment, hope, self-esteem, and psychiatric symptoms. As well, participants will complete two brief questionnaires pertaining to: 1) education (includes skills training), employment, and income; and 2) perceived housing quality. **QL** = qualitative measures (12 participants). These will consist of one-on-one semi-structured interviews with the same 12 participants (six from each arm). The interview questions will explore issues related to feasibility and acceptability, and provide context to the quantitative responses.

4. Data Generation

Undertaking a mixed methods study where data is truly 'mixed' at the level of collection (not just at analysis) is challenging because it requires a solid understanding of the data generation requirements of each research paradigm, and the interviews can take longer to conduct than those focusing on qualitative or quantitative

methods alone (Farquhar, Ewing, & Booth, 2011). To minimize respondent burden, we have given careful consideration to the type of quantitative instruments chosen (e.g., number and length of time to complete) and will ensure that appropriate components of the qualitative interviews are prioritized (e.g., follow-up on changes in instrument scores) at each data generation session (Farquhar et al.). Additionally, all of the mixed method interviews will be conducted by the co-investigator, Dr. Naomi Thulien, who has expertise in conducting mixed method interviews with young people who have experienced homelessness.

4a. Study Outcomes

As mentioned previously, the mixed methods design of this pilot RCT reflects our desire to capture the complex independent and interdependent explanatory pathways of the intervention. This is especially crucial during this pilot stage, where we will be paying particular attention to feasibility, context, and unexpected mechanisms that produce change – factors that will influence study outcomes and provide important information regarding scalability and sustainability (Craig et al., 2008; Moore et al., 2015). To fully apprehend these complex explanatory pathways, "represent the best use of the data," and "provide an adequate assessment of the success or otherwise of an intervention that has effects across a range of domains" (Craig et al., p.3), we have aligned our key outcome variables (Table 1) with the Medical Research Council guidance on evaluating complex interventions and identified more than one primary outcome measure.

Table 1. Key Outcome Variables

**Variables**	**Instruments***
Community integration (psychological and physical)<br>Social connectedness<br>Engulfment	Community Integration Scale<br>Social Connectedness Scale – Revised<br>Modified Engulfment Scale
Self-esteem<br>Hope<br>Psychiatric symptoms	Rosenberg Self-Esteem Scale<br>Beck Hopelessness Scale<br>Modified Colorado Symptom Index
Enrollment in education (includes skills training)<br>Employment	Composite checklist
Income	Composite checklist
Perceived housing quality	Perceived Housing Quality Scale

Participant perspectives of barriers and facilitators	Individual semi-structured interviews (youth) and focus groups (mentors)<br>Composite checklist (Mentor Evaluation)

*See Table 2 for references and psychometric properties. All will be administered every six months for 30 months (except for the mentor evaluation – see Table 2).

The **primary outcome measures** for this study are: community integration (psychological and physical) and self-esteem. **Secondary outcomes** include: social connectedness, hope, and academic and vocational participation. **Exploratory outcomes** include: engulfment, psychiatric symptoms, income, perceived housing quality, and participant perspectives of intervention barriers and facilitators.

4b. Study Hypothesis

We hypothesize that, for the **primary outcome** measures of community integration and self-esteem:

1. We will observe better mean scores (community integration and self-esteem) in the participants who receive rent subsidies plus mentorship (intervention group) compared to the participants who receive rent subsidies only (control group) by our **primary endpoint of 18 months** of study participation.

We hypothesise that, for the **secondary outcome** measures of social connectedness, hope, and academic and vocational participation:

1. We will observe better mean scores (social connectedness and hope) in the intervention group relative to participants in the control group by our **secondary endpoint of 18 months** of study participation.
2. Participants in the intervention group will be more likely than the control group to demonstrate sustained engagement in academic and vocational activities (education, employment, and/or skills training) by our **secondary endpoint of 18 months** of study participation.

4c. Quantitative Measures

Quantitative data (Appendix A – Quantitative Data Collection) will be collected at **six points** in time over the course of 30 months: baseline, month six, month 12, month 18, month 24, and month 30. **Nine instruments** (Table 2) will be employed to assess the outcome variables. We have purposely chosen instruments utilized in previous research with young people who have experienced homelessness (e.g., Kidd et al., 2016; Kozloff et al., 2016; McCay et al., 2015) so that meaningful comparisons can be made across studies (Moore et al., 2015) in this nascent area of research. One instrument we do not believe has been used with this population is the Modified Engulfment Scale (McCay & Seeman, 1998). We have included engulfment as an exploratory outcome given the emerging qualitative evidence on the crucial role of identity (self-concept) in a young

person's transition away from homelessness (Brueckner et al., 2011; Karabanow et al., 2016; Thulien et al., 2018).

Table 2. Quantitative Instruments

Instrument	Psychometric Information
Beck Hopelessness Scale (Beck, Weissman, Lester, & Trexler, 1974).	This 20-item scale measures motivation, expectations, and feelings about the future (internal consistency α = .93).
Community Integration Scale (Stergiopoulos et al., 2014).	This 11-item scale measures behavioral (e.g., participation in activities) and psychological (e.g., sense of belonging) aspects of community integration. This scale was used extensively in the Chez Soi/At Home study, but psychometric properties have yet to be reported.
Education, Employment, and Income Questionnaire	This 13-item questionnaire assesses education, employment, and income. We developed this questionnaire for the study.
Mentor Evaluation Questionnaire	This 10-item questionnaire assesses mentor effectiveness. It will be completed at month 24 by those in the intervention group. We developed this questionnaire for the study in collaboration with our community partners.
Modified Colorado Symptom Index (Ciarolo et al., 1981).	This 14-item scale measures the presence and frequency of psychiatric symptoms experienced in the past month. (internal consistency α = .90 – .92).
Modified Engulfment Scale (McCay & Seeman, 1998).	This 30-item scale measures the degree to which an individual's self-concept is defined by their experience of homelessness (internal consistency α = .91). We have adapted the scale for this study, substituting "experience of homelessness" for "illness".
Perceived Housing Quality (Toro et al., 1997).	This seven-item scale measures participant perception of housing choice and quality. This scale was used extensively in the Chez Soi/At Home study, but psychometric properties have yet to be reported. We have shortened it from 10 items (Chez Soi/At Home) to seven relevant items.
Rosenberg Self-Esteem Scale (Rosenberg, 1965).	This 10-item scale measures global self-worth (internal consistency α = .77 – .88).
Social Connectedness Scale	This 20-item scale measures belongingness – the degree to which

– Revised (Lee & Robbins, 1995).	an individual feels connected to others (internal consistency α = .92).

4d. Qualitative Measures

Qualitative measures (see Appendix B – Qualitative Data Generation) are an important feature of this study and will consist of: **1) semi-structured individual interviews** (study participants) and **2) focus groups** (mentors).

At baseline, twelve participants (six from each arm of the study) will be invited to participate in **six semi-structured individual interviews**, which will take place at the same time as the quantitative data collection: **baseline, month six, month 12, month 18, month 24, and month 30**. Participants will be purposively selected with a goal of having input from each of the three communities and a fairly equal gender and ethno-racial representation.

All of the mentors (n = 12) will be invited to participate in **two focus groups**, which will take place at **month 12 and month 24**.

The questions posed during the semi-structured interviews and focus groups will be guided by the study objectives, but will be conversational and exploratory in nature with particular attention to understanding *how* mentoring and/or rent subsidies influence social integration outcomes for formerly homeless young people living in market rent housing. Given the emergent nature of qualitative inquiry (Denzin & Lincoln, 2011; Eakin & Mykhalovskiy, 2003), we expect the interview and focus group questions to evolve over time as key preliminary themes begin to surface. It is anticipated that the individual interviews (including quantitative data collection, which will consistently take place first) will last approximately 60 – 75 minutes, and the focus groups approximately 60 – 90 minutes. The individual interviews and the focus groups will be conducted by Dr. Naomi Thulien at locations most convenient for those participating. To get a better sense of each young person's living situation and to minimize researcher – participant power imbalance (Israel et al., 2018), Dr. Thulien will suggest that the individual interviews take place in or nearby the young people's homes. The individual interviews and focus groups will be audio recorded and transcribed verbatim.

4e. Honoraria

All of the study participants will be paid an honorarium of $20 at each of the six quantitative data collection points. Those participating in semi-structured interviews will be paid an additional $30 at each interview. This

amount was based on the co-investigator's previous experience with this population and after consulting with our community partners.

5. Data Analysis

One major critique of mixed methods RCTs is that, typically, there is no true integration (i.e., 'mixing') of quantitative and qualitative findings at the level of analysis or interpretation (Lewin, Glenton, & Oxman, 2009). Moreover, it is often unclear whether or how the quantitative and qualitative researchers have worked together to maximize the potential synergies between these different approaches (Lewin et al.). With this in mind, our study team, consisting of researchers with quantitative and qualitative expertise, worked together to develop this study protocol and anticipate meeting quarterly to discuss the emerging analysis and to explore (and follow up on) similarities or discrepancies between the quantitative and qualitative data.

5a. Quantitative Data

All analyses will be performed using the intention-to-treat principle; that is, all participants will be included and analyzed in the groups they were originally randomized. Baseline characteristics of the intervention and control groups will be summarized using descriptive statistics (i.e., mean, standard deviation, median and interquartile range for continuous variables, and frequencies and proportions for categorical variables). We will also calculate descriptive statistics for outcomes at each study time point, and will explore differences in trajectories from baseline to 30 months follow-up between intervention and control groups using scatterplots and box-plots. Differences with 95% confidence intervals in continuous outcomes at 18 months (psychological community integration, self-esteem, social connectedness, hope, perceived housing quality, psychiatric symptoms, and sense of engulfment) between participants who received rent subsidies plus mentorship and participants who only received rent subsidies will be estimated using Analysis of Covariance (i.e., linear regression models), including an indicator of intervention group and the baseline value of the outcome. We will perform regression diagnostics and will repeat analyses using the non-parametric Wilcoxon rank-sum test if there are extreme outliers or influential observations. Groups will be compared with respect to count outcomes at 18 months (physical community integration) using graphical tools and the non-parametric Wilcoxon rank-sum test. For binary outcomes at 18 months (sustained academic and vocational participation, and income above low income cut-off[4]), differences in proportions with 95% confidence intervals will be estimated and tested using the chi-square or Fisher's exact test. Given the small sample size of this pilot randomized trial, all results will be interpreted with caution and with the intention of generating

---

[4] Based on family and community size.

data and hypotheses for conducting a larger trial.

All efforts will be made to reduce participants' attrition and drop-out. As mentioned previously, we believe our strong relationship with the outreach workers and community agencies will help minimize loss to follow-up (Slesnick et al., 2009). In addition, we have made it very clear in the participant information and consent form that participants in the rent subsidies plus mentorship arm may continue in the study (receiving only rent subsidies) if they are unable to continue in a mentor-mentee relationship.

5b. Qualitative Data

In keeping with the emergent, iterative nature of research using a qualitative design (Denzin & Lincoln, 2011; Eakin & Mykhalovskiy, 2003), data analysis and interpretation will begin immediately after the first qualitative data generation session (at baseline). The semi-structured individual interviews and focus groups will be audio recorded and transcribed verbatim. In order to conduct a more nuanced analysis of the data, the transcriptionist will be instructed to note short responses, uncooperative tones, and literal silence (Eakin & Mykhalovskiy; Kawabata & Gastaldo, 2015). Prior to each subsequent qualitative data generation session, members of the research team will conduct a preliminary data analysis, reading the interview transcripts multiple times, separating the data into coded segments, making analytic memos beside sections of the transcripts, identifying emerging themes (and comparing/contrasting these between respondents), and compiling new questions (Creswell, 2014; Denzin & Lincoln). Those participating in the individual interviews and the focus groups will be asked for their perspectives on the emerging interpretations at each visit and these perspectives will play a key role in helping shape the data analysis and help ensure the trustworthiness of the data (Creswell; Loiselle, Profetto-McGrath, Polit, & Tatano Beck, 2004). The web-based application Dedoose (SocioCultural Research Consultants, LLC, 2018) will be utilized to assist with sorting and coding the qualitative data.

6. Ethical Considerations

There are important ethical considerations that must be considered with any type of research. This is especially true of RCTs conducted with marginalized populations (Solomon et al., 2009). Accordingly, we have endeavored to weave ethical considerations into all aspects of the study design (Solomon et al.), including our decision to utilize a CBPAR methodology and to modify the study design so that all of the participants will receive rent subsidies. Ethical approval for this study will be obtained from the Providence St. Joseph's and St. Michael's Healthcare Research Ethics Board (REB).

6a. Benefits and Risks to Participants

All of the study participants will likely find it beneficial to receive rent subsidies. Those randomized to the intervention group may also benefit from receiving regular interactions with a mentor. Participants selected for qualitative interviews might benefit from the opportunity to share their integration-related experiences with Dr. Thulien on a regular basis. Additionally, participants may derive satisfaction from knowing that their contributions will help advance our understanding about how best to design interventions that assist formerly homeless young people to achieve meaningful social integration.

We believe theoretical justification exists for expecting that the proposed mentoring intervention is likely to produce effective outcomes; however, research ethics demands that we mitigate any potential risk to the research participants (Solomon et al., 2009). As previously mentioned, we will rely on the expertise of our community partners to screen and train the mentors. In addition, as highlighted previously, the outreach workers will work closely with the study participants and with our research team, and will alert our team if there are any concerns about a mentor-mentee relationship. These concerns will be relayed to our community partners so they can take appropriate action. The Providence St. Joseph's and St. Michael's Healthcare REB will be notified if a mentor-mentee relationship is terminated due to actions that violate the mentor-mentee code of conduct.

Participants will be assured that their participation or lack of participation in the study will not negatively impact their relationship with our community partners or their ability to access services at St. Michael's Hospital or support from other social services agencies (e.g., OW/ODSP).

6b. Privacy and Confidentiality

This mixed methods RCT will use multiple and varied data sources. This comprehensiveness is critical to the objectives of the study, yet may increase invasion of participant privacy. This privacy concern will be clearly communicated to potential participants, as will the measures for protecting security and confidentiality, prior to consent.

All of the data collected will be kept in strict confidence. While participants' names will appear on the consent forms, pseudonyms (created by the participants) will be used in place of their real names on all documents related to data generation, including the audio recordings and interview transcripts. A key that links each participant name with a pseudonym will be created and stored as a separate electronic file. All electronic data will be stored on secure servers at the Centre for Urban Health Solutions or the McMaster University School of Nursing, and only be accessible by select members of the research team.

The individual interviews and focus groups will be audio recorded using a password protected application on a password protected electronic device. The audio recordings from the individual interviews and focus groups will be deleted once the transcripts have been stored on the secure server and entered into Dedoose (encrypted and password-protected) (SocioCultural Research Consultants, LLC, 2018). Paper copies of the data (e.g., consent forms and standardized quantitative measures) will be stored in a locked filing cabinet at the Centre for Urban Health Solutions or at the McMaster University School of Nursing – areas only accessible to those with electronic and key access. All paper and electronic files will be retained for a period of up to five years from study closure.

The consent form indicates that limits to confidentiality apply if a participant discloses that they intended to hurt themselves or others, or if they inform a member of the research team that someone under the age of 16 years is suffering abuse and/or neglect.

7. Dissemination

In keeping with our CBPAR methodology, we are committed to disseminating evidence *with* our community partners to build community capacity and improve the lives of the young people participating in this study (Chenail et al., 2012; Wallerstein et al., 2018). Moreover, given our use of Critical Social Theory, we are obliged to not only present our findings, but to expose and explicate the relational processes (e.g., subjective experience of low socioeconomic position and low social class) that may be preventing formerly homeless young people from achieving meaningful social integration (Madison, 2012; Strega, 2005). With an emphasis on 'actionable' data (Chenail et al.), we anticipate disseminating our findings broadly to both academic and community-based audiences in a variety of formats ranging from scientific journal papers to oral presentations.

8. Limitations

This study has a number of limitations. First, the young people recruited for this pilot study will be a small sample of youth connected to urban-based social service providers in the province of Ontario. Thus, the findings may not be generalizable to formerly homeless young people living in other contexts and/or not connected to social service agencies. Second, the quantitative instruments are based on self-report and may involve a degree of response bias. Finally, the quantitative measures we have chosen are what we believe to be surrogate markers of meaningful social integration. Future work will likely be needed to more accurately capture this complex concept.

9. Significance

This pilot RCT study will be the first to test the impact of economic and social supports on meaningful social integration for formerly homeless young people living in market rent housing. We believe the mixed methods design will illuminate important contextual factors that must be considered if the intervention is to be scaled up and replicated elsewhere. Importantly, the CBPAR framework will incorporate the perspectives of the community, including formerly homeless young people, who are in the best position to determine what might work best in the context of their lives.

References

Altena, A. M., Brilleslijper-Kater, S. N., & Wolf, J. R. (2010). Effective interventions for homeless youth: A systematic review. *American Journal of Preventive Medicine*, *38*(6), 637-645. doi: 10.1016/j.amepre.2010.02.017.

Beck, A. T., Weissman, A., Lester, D., & Trexler, L. (1974). The measurement of pessimism: The hopelessness scale. *Journal of Consulting and Clinical Psychology, 41*(6), 639–660. doi: 10.1037/h0037562.

Brueckner, M., Green, M., & Saggers, S. (2011). The trappings of home: Young homeless people's transitions towards independent living. *Housing Studies*, *26*(1), 1-16. doi:10.1080/02673037.2010.512751.

Chenail. R. J., St. Goerge, S., Wulff, D., & Cooper, R. (2012). Action research: The methodologies. In P. L. Munhall (Ed.), *Nursing research: A qualitative perspective* (5th ed.) (pp. 455-470). Sudbury, MS: Jones & Bartlett.

Ciarolo, J. A., Edwards, D. W., Kiresuk, T. J., Newman, F. L., & Brown, T. R. (1981). *Colorado symptom index.* Washington, DC: National Institute of Mental Health.

Coren, E., Hossain, R., Pardo Pardo, J., & Bakker, B. (2016). Interventions for promoting reintegration and reducing harmful behaviour and lifestyles in street-connected children and young people. *Cochrane database of systematic reviews*, *2016*(1), 1-152. doi: 10.1002/14651858.CD009823.pub3.

Craig, P., Dieppe, P., Macintyre, S., Michie, S., Nazareth, I., & Petticrew, M. (2008). Developing and evaluating complex interventions: The new Medical Research Council guidance. *BMJ*, *337*(a1655), 1-6. doi: 10.1136/bmj.a1655.

Creswell, J.W. (2014). *Research design: Qualitative, quantitative, and mixed method approaches* (4th ed.). Thousand Oaks, CA: Sage.

Creswell, J.W., & Plano Clark, V.L. (2018). Designing and conducting mixed methods research (3rd ed.). Thousand Oaks, CA: Sage.

Dang, M. T., & Miller, E. (2013). Characteristics of natural mentoring relationships from the perspectives of homeless youth. *Journal of Child and Adolescent Psychiatric Nursing*, *26*(4), 246-253. doi: 10.1111/jcap.12038.

Denzin, N.K., & Lincoln, Y.S. (2011). *The SAGE handbook of qualitative research*. Thousand Oaks, CA: SAGE Publications Inc.

Eakin, J.M., & Mykhalovskiy, E. (2003). Reframing the evaluation of qualitative health research: Reflections on a review of appraisal guidelines in the health sciences. *Journal of Evaluation in Clinical Practice*, *9*(2), 187-194. doi: 10.1046/j.1365-2753.2003.00392.x.

Efird, J. (2011). Block randomization with randomly selected block sizes. *International Journal of Environmental Research and Public Health, 8*(1), 15-20. doi: 10.3390/ijerph8010015.

Farquhar, M. C., Ewing, G., & Booth, S. (2011). Using mixed methods to develop and evaluate complex interventions in palliative care research. *Palliative Medicine*, *25*(8), 748-757. doi: 10.1177/0269216311417919.

Fletcher, R.H., Fletcher, S.W., & Fletcher, G.S. (2014). *Clinical epidemiology: The essentials* (5th ed.). Philadelphia, PA: Lippincott Williams & Wilkins.

Frederick, T., Chwalek, M., Hughes, J., Karabanow, J., & Kidd, S. (2014). How stable is stable? Defining and measuring housing stability. *Journal of Community Psychology, 42*(8), 964- 979. doi:10.1002/jcop.21665.

Gaetz, S. (2014). *Coming of age: Reimagining the response to youth homelessness in Canada.* Toronto, ON: The Canadian Homelessness Research Network Press. Retrieved from http://www.homelesshub.ca/comingofage

Gaetz, S., Dej, E., Richter, T., & Redman, M. (2016) *The state of homelessness in Canada 2016.* Toronto, ON: Canadian Observatory on Homelessness Press. Retrieved from http://www.homelesshub.ca/SOHC2016

Gaetz, S., O'Grady, B., Kidd, S., & Schwan, K. (2016). *Without a home: The national youth homelessness survey.* Toronto: Canadian Observatory on Homelessness Press. Retrieved from http://homelesshub.ca/sites/default/files/WithoutAHome-final.pdf

Gaetz, S., & Redman, M. (2016). *Federal investment in youth homelessness: Comparing Canada and the United States and a proposal for reinvestment. Canadian observatory on homelessness policy brief.* Toronto, ON: The Homeless Hub Press. Retrieved from http://homelesshub.ca/sites/default/files/Policy_Brief.pdf

Goodkind, J. R., Amer, S., Christian, C., Hess, J. M., Bybee, D., Isakson, B. L., ...

Shantzek, C. (2017). Challenges and innovations in a community-based participatory randomized controlled trial. *Health Education & Behavior*, *44*(1), 123-130. doi: 10.1177/1090198116639243

Hammersley, M., & Atkinson, P. (2007). *Ethnography: Principles in Practice* (3rd ed.). London, UK: Routledge.

Hwang, S.W., & Burns, T. (2014). Health interventions for people who are homeless. *The Lancet, 384*(9953), 1541-1547. doi: 10.1016/S0140-6736(14)61133-8.

Israel, B.A., Schulz, A.J., Parker, E.A., Becker, A.B., Allen, III, Guzman, R.J., & Lichtenstein, R. (2018). Critical issues in developing and following CBPR principles. In N. Wallerstein, B. Duran, J. Oetzel, & M. Minkler (Eds.), *Community-based participatory research for health: Advancing social and health equity* (3rd ed.) (pp. 31-44). San Francisco, CA: Jossey-Bass.

Karabanow, J. (2008). Getting off the street: Exploring the process of young people's street exits. *American Behavioral Scientist, 51*(6)*,* 772-788. doi:10.1177/0002764207311987. Karabanow, J., Carson, A., & Clement, P. (2010). *Leaving the streets: Stories of Canadian youth*. Halifax, NS: Fernwood Publishing.

Karabanow, J., Kidd, S., Frederick, T., & Hughes, J. (2016). Toward housing stability: Exiting homelessness as an emerging adult. *Journal of Sociology & Social Welfare, 43*(1), 121- 148. Retrieved from https://wmich.edu/socialworkjournal

Kawabata, M., & Gastaldo, D. (2015). The less said, the better: Interpreting silence in qualitative research. *International Journal of Qualitative Research Methods, 14*(4), 1-9. doi:10.1177/1609406915618123.

Kidd, S.A., Frederick, T., Karabanow, J., Hughes, J., Naylor, T., & Barbic, S. (2016). A mixed methods study of recently homeless youth efforts to sustain housing and stability. *Child and Adolescent Social Work Journal*, *33*(3), 207-218. doi:10.1007/s10560-015-0424.

Kozloff, N., Adair, C. E., Lazgare, L. I. P., Poremski, D., Cheung, A. H., Sandu, R., & Stergiopoulos, V. (2016). "Housing first" for homeless youth with mental illness. *Pediatrics*, *138*(4), e20161514. doi: 10.1542/peds.2016-1514

Kulik, D.M., Gaetz, S., Crowe, C., & Ford-Jones, E.L. (2011). Homeless youth's overwhelming health burden: A review of the literature. *Paediatric Child Health*, *16*(6), e43-e47. doi: 10.1093/pch/16.6e43.

Kusenbach, M. (2003). Street phenomenology: The go-along as ethnographic research tool. *Ethnography, 4*(3), 455-485. doi: 10.1177/146613810343007.

Lee, R. M., & Robbins S. B. (1995). Measuring belongingness: The social connectedness and the social assurance scales. *Journal of Counseling Psychology*, *42*(2), 232-241. doi: 10.1037/0022-0167.42.2.232.

Lewin, S., Glenton, C., & Oxman, A. D. (2009). Use of qualitative methods alongside randomised controlled trials of complex healthcare interventions: methodological study. *BMJ*, *339*(b3496), 1-7. doi: 10.1136/bmj.b3496.

Loiselle, C.G., Profetto-McGrath, J., Polit, D.F., & Tatano Beck, C.T. (2004). *Canadian essentials of nursing research*. Philadelphia, PA: Lippincott Williams & Wilkins.

Luchenski, S., Maguire, N., Aldridge, R. W., Hayward, A., Story, A., Perri, P., ... Hewett, N. (2017). What works in inclusion health: overview of effective interventions for marginalised and excluded populations. *The Lancet*, *391*(10117), 266-280. doi:10.1016/ S0140-6736(17)31959-1.

Madison, D.S. (2012). *Critical ethnography: Method, ethics, and performance* (2nd ed.). Thousand Oaks, CA: Sage Publication, Inc.

Mayock, P., O'Sullivan, E., & Corr, M.L. (2011). Young people exiting homelessness: An exploration of process, meaning and definition. *Housing Studies, 26*(6), 803-826. doi:10.1080/02673037.2011.593131.

McCay, E., Carter, C., Aiello, A., Quesnel, S., Langley, J., Hwang, S., ... Karabanow, J. (2015). Dialectical Behavior Therapy as a catalyst for change in street-involved youth: A mixed methods study. *Children and Youth Services Review*, *58*, 187-199. doi: 10.1016/j.childyouth.2015.09.021.

McCay, E. & Seeman, M. (1998). A scale to measure the impact of a schizophrenic illness on an individual's self-concept. *Archives of Psychiatric Nursing, 12(1),* 41-49. doi: 10.1016/S0883-9417(98)80007-1.

Milburn, N.G., Rice, E., Rotheram-Borus, M.J., Mallett, S., Rosenthal, D., Batterham, P., ... Duan, N. (2009). Adolescents exiting homelessness over two years: The risk amplification and abatement model. *Journal of Research on Adolescence, 19*(4), 762- 785. doi:10.1111/j.1532-7795.2009.00610.x.

Moore, G. F., Audrey, S., Barker, M., Bond, L., Bonell, C., Hardeman, W., ... Baird, J. (2015). Process evaluation of complex interventions: Medical Research Council guidance. *BMJ, 350*(h1258), 1-6. doi: 10.1136/bmj.h1258.

Ontario Human Rights Commission. (n.d.). *Housing as a human right.* Retrieved from http://www.ohrc.on.ca/en/right-home-report-consultation-human-rights-and-rental-housing-ontario/housing-human-right

Popay, J., Escorel, S., Hernandez, M., Johnston, H., Mathieson, J., & Rispel, L. (2008). *Understanding and tackling social exclusion: Final report to the WHO commission on social determinants of health from the social exclusion knowledge network*. Retrieved from http://www.who.int/social_determinants/themes/socialexclusion/en/

Prasad, P. (2005). *Crafting qualitative research: Working in the postpositivist traditions.* New York, NY: M.E. Sharpe.

Public Interest. (2009). *Changing patterns for street involved youth.* Toronto, ON: Author. Retrieved from http://www.worldvision.ca/Programs-and- Projects/Canadian Programs/Documents/ChangingPatternsForStreetInvolvedYouth.pdf

Quilgars, D., & Pleace, N. (2016). Housing First and Social Integration: A Realistic Aim? *Social Inclusion*, *4*(4), 5-15. doi:10.17645/si.v4i4.672.

Rosenberg, M. (1965). Society and the adolescent self-image. Princeton, NJ: Princeton University Press.

Rutman, A., Hubberstey, A., Barlow, A., & Brown, E. (2005). Supporting young people's transitions from care: Reflections on doing participatory action research with youth from care. In L. Brown & S. Strega (Eds.), *Research as resistance: Critical, Indigenous, & anti-oppressive approaches* (pp. 153-179). Toronto, ON: Canadian Scholars' Press/Women's Press.

Slesnick, N., Dashora, P., Letcher, A., Erdem, G., & Serovich, J. (2009). A review of services and interventions for runaway and homeless youth: Moving forward. *Children and Youth Services Review*, *31*(7), 732-742. doi: 10.1016/j.childyouth.2009.01.006.

SocioCultural Research Consultants, LLC. (2018). Dedoose (Version 8.0.35) [web application]. Retrieved from http://www.dedoose.com

Solar, O., & Irwin, A. (2010). *A conceptual framework for action on the social determinants of health: Social determinants of health discussion paper 2.* Geneva, Switzerland: World Health Organization Press. Retrieved from http://www.who.int/sdhconference/resources/ConceptualframeworkforactiononS DH_eng.pdf

Solomon, P., Cavanaugh, M.M., & Draine, J. (2009). *Randomized controlled trials: Design and implementation for community-based psychosocial interventions.* New York, NY: Oxford University Press.

Stergiopoulos, V., Gozdzik, A., O'Campo, P., Holtby, A., Jeyaratnam, J., & Tsemberis, S. (2014). Housing first: Exploring participants' early support needs. *BMC Health Services Research, 14*(167), 1-15. doi:10.1186/1472-6963-14-16.

Strega, B. (2005). The view from the poststructural margins: Epistemology and methodology reconsidered. In L. Brown & S. Strega (Eds.), *Research as resistance: Critical, Indigenous, & anti-oppressive approaches* (pp. 199-235). Toronto, ON: Canadian Scholars' Press/Women's Press.

Suresh, K. P. (2011). An overview of randomization techniques: an unbiased assessment of outcome in clinical research. *Journal of human reproductive sciences*, *4*(1), 8-11. doi: 10.4103/0974-1208.82352.

Thompson, A. E., Greeson, J. K., & Brunsink, A. M. (2016). Natural mentoring among older

youth in and aging out of foster care: A systematic review. *Children and Youth Services Review*, *61*, 40-50. doi: 10.1016/j.childyouth.2015.12.006.

Thulien, N. S., Gastaldo, D., Hwang, S. W., & McCay, E. (2018). The elusive goal of social integration: A critical examination of the socio-economic and psychosocial consequences experienced by homeless young people who obtain housing. *Canadian Journal of Public Health*, *109*(1), 89-98. doi: 10.17269/s41997-018-0029-6.

Toro, P. A., Rabideau, J. M. P., Bellavia, C. W., Daeschler, C. V., Wall, D. D., Thomas, D. M., & Smith, S. J. (1997). Evaluating an intervention for homeless persons: results of a field experiment. *Journal of consulting and clinical psychology*, *65*(3), 476-484. doi: 10.1037//0022-006X.65.3.476.

Van Dam, L., Smit, D., Wildschut, B., Branje, S. J. T., Rhodes, J. E., Assink, M., & Stams, G. J. J. M. (2018). Does natural mentoring matter? A multilevel meta-analysis on the association between natural mentoring and youth outcomes. *American Journal of Community Psychology, 0,* 1-18. doi: 10.1002/ajcp.12248.

Wallerstein, N. & Duran, B. (2006). Using community-based participatory research to address health disparities. *Health Promotion Practice, 7*(3), 312-323. doi: 10.1177/1524839906289376.

Wallerstein, N. & Duran, B. (2010). Community-based participatory research contributions to intervention research: The intersection of science and practice to improve health equity. *American Journal of Public Health, 100*(S1), S40-S46. doi: 10.2105/AJPH.2009.

Wallerstein, N., Duran, B., Oetzel, J.G., & Minkler, M. (2018). *Community-based participatory research for health: Advancing social and health equity* (3rd ed.). San Francisco, CA: Jossey-Bass.

Transitioning Youth Out of Homelessness: A Mixed Methods Community-Based Pilot Randomized Controlled Trial of a Rent Subsidy and Mentoring Intervention in Three Canadian Cities

1. Background and Rationale

Young people comprise almost 20% of the homeless population in Canada (Gaetz, Dej, Richter, & Redman, 2016). It is estimated between 35,000 and 40,000 Canadian youth (ages 13 – 25) are homeless at some point during the year and at least 6,000 on any given night (Gaetz, O'Grady, Kidd, & Schwan, 2016; Gaetz & Redman, 2016).

We know a great deal about the risk factors associated with young people entering and becoming entrenched in street life (e.g., intergenerational poverty, childhood abuse, inadequate education, and limited employment opportunities), but we know much less about how to facilitate and sustain transitions off the streets (Karabanow, 2008; Kidd et al., 2016; Kulik, Gaetz, Crowe, & Ford-Jones, 2011; Mayock, O'Sullivan, & Corr, 2011). In fact, in the peer-reviewed literature, the evidence is scarce to non-existent for rigorous interventions targeting housing outcomes, life trajectories, quality of life, and social integration[1] for young people experiencing homelessness (Altena, Brilleslijiper-Kater, & Wolf, 2010; Coren, Hossain, Pardo, & Bakker, 2016; Hwang & Burns, 2014; Luchenski et al., 2017). Understanding how to create and support successful pathways out of homelessness is crucial, because once youth become entrenched in street life, it becomes much harder for them to exit homelessness and escape a life of poverty (Gaetz, 2014; Karabanow, Carson, & Clement, 2010; Milburn et al., 2009; Public Interest, 2009).

Intuitively, it may seem that one important way to improve the life trajectories of young people experiencing homelessness is to provide them with a home. However, from the limited research that has been done in this area, we know that formerly homeless young people continue to experience significant challenges – particularly when it comes to mainstream social integration – even after they are 'successfully' housed (Thulien, Gastaldo, Hwang, & McCay, 2018). Moreover, these challenges seem to persist regardless of the type of housing (e.g., subsidized vs. market rent) provided (Brueckner, Green, & Saggers, 2011; Kidd et al., 2016; Kozloff et al., 2016).

[1] The concept of social integration is complex and often inconsistently defined and poorly measured (Quilgars & Pleace, 2016). For the purpose of this study, we drew from the literature on the social determinants of health and social exclusion, and adopted a holistic definition of social integration, incorporating both the tangible (e.g., access to education and a living wage) and intangible (e.g., sense of connection and belonging) aspects of meaningful and equitable societal participation (Luchenski et al., 2017; Popay et al., 2008; Solar & Irwin, 2010).

Evidence from one of the most rigorous longitudinal studies with formerly homeless youth (ages 16 – 25) to date highlights that the procurement of a home does not necessarily translate into a sense of belonging or connection to mainstream society (Kidd et al., 2016). This mixed methods study of 51 formerly homeless young people living in two major urban centers in Canada showed that, despite living in stable or semi-stable accommodations (53% lived in subsidized housing), participants continued to face substantial challenges such as poverty-level incomes and limited mainstream social networks which, over the course of one year, contributed to a significant decline in hope, no gains in community integration, and a sense of being 'stuck' (Frederick, Chwalek, Hughes, Karabanow, & Kidd, 2014; Karabanow, Kidd, Frederick, & Hughes, 2016; Kidd et al.). Moreover, community integration challenges were significantly worse for participants living in independent (market rent) housing.

A sub-group analysis of 156 young people (ages 18 – 24) with mental health challenges who participated in a 24-month randomized controlled trial (RCT) of 'Housing First' (access to subsidized housing and comprehensive social service supports [e.g., treatment for mental health challenges] at home or in the community) in five Canadian cities – the largest RCT of Housing First to date – indicate similar findings of ongoing hardship despite achieving housing stability (Kozloff et al., 2016). While the young people who received the Housing First intervention achieved significantly better housing stability compared to the treatment as usual group, they did not experience any additional improvements to other outcomes such as employment, generic quality of life, and community integration relative to treatment as usual (Kozloff et al.). Notably, the same community integration scale (Stergiopoulos et al., 2014) was used in this RCT and the aforementioned Kidd et al. study, and measures both physical integration (e.g., attending a movie or community event) and psychological integration (e.g., interactions with others and feeling like one belongs).

Findings from a ten-month ethnographic study with nine formerly homeless young people (ages 18 – 24) living in Canada's largest city also support the idea that transitioning young people out of homelessness and helping them integrate into the mainstream likely requires much more than simply providing them with a home (Thulien et al., 2018). This study is believed to be the first ethnographic study to exclusively focus on the integration experiences of formerly homeless young people living in market rent housing and showed that, despite the appearance of housing stability, the participants were living a precarious existence, attributed in part to the chronic stress and exhaustion of living in poverty and to their limited knowledge about how to move forward in life (Thulien et al.). In addition, the authors note that participants underutilized transition-related social supports (e.g., food banks and employment counseling) because these supports tended to be

deficit-focused (e.g., focused on what youth did not have, not on what they had achieved) and located in areas (e.g., homeless shelters) that reminded them of their old identities as homeless youth.

As previously mentioned, little evidence exists for effective interventions that target social integration for young people who have experienced homelessness. This includes evidence on the impact of mentorship. In fact, for formal mentorship programs in general, meta-analyses have only found small overall positive effect sizes (i.e., the impact of the average mentoring program in improving youth outcomes) on the psychological, emotional, behavioral, and educational functioning of participating young people (Thompson, Greeson, & Brunsink, 2016; Van Dam et al., 2018). However, there is some emerging evidence on the benefits of 'natural mentors' – generally defined as an important, encouraging, non-parental adult that exists in a youth's social network – that may be transferrable to youth who have experienced homelessness.

A systematic review of natural mentoring for youth (ages 13 – 25) transitioning out of foster care showed that the young people benefited from a supportive adult not "tasked with enforcing daily rules and addressing misbehavior" (p. 48) and that this intervention resulted in improved behavioral, psychosocial, and academic outcomes (Thompson et al.). The authors stress the importance of cultivating *inter*dependence (as opposed to independence) for young people leaving foster care and suggest that, while traditional natural mentoring relationships tend to emerge organically, they can be facilitated and supported programmatically as well (see https://vimeo.com/115837436).

A more recent meta-analysis of natural mentoring in youth (ages 13 – 24) also supports the notion that the presence of a natural mentor can positively impact young people (Van Dam et al., 2018). This meta-analysis included all young people (not just 'at-risk' youth) and found that, similar to the aforementioned systematic review, positive youth outcomes were particularly significant in the domains of social and emotional development, and academic and vocational functioning (Van Dam et al.). Moreover, the authors found that risk status (e.g., young people who were homeless or living in foster care) did *not* moderate these positive outcomes.

While almost all of the reviewed studies of at-risk youth in the meta-analysis and the systematic review were limited by their cross-sectional design, the results do hold promise for mentoring interventions that incorporate the positive characteristics of natural mentors (i.e., more of a friendship-like, 'coach', or 'cheerleader' role) for young people who have experienced homelessness. These findings are supported by a small (n = 23) qualitative study of natural mentoring relationships among homeless youth (ages 14 – 21) that

suggests “natural mentors could feasibly serve as a bridge in a coordinated effort to assist youth out of homelessness” (Dang & Miller, 2013, p. 7).

From the limited research that has been done with young people transitioning away from homelessness, the emerging evidence seems to indicate that, while structural supports such as subsidized housing and social service providers are important, these things alone are insufficient to help young people integrate into mainstream society. As it currently stands, it appears as if the burden for achieving meaningful social integration is on the formerly homeless young people, who continue to be marginalized despite achieving stable or semi-stable housing (Quilgars & Pleace, 2016; Thulien et al., 2018). Connecting these young people with an adult who exhibits the relationship-based components of natural mentoring that young people value most (e.g., genuine interest in their well-being and belief in their ability to succeed, a non-judgmental attitude and a willingness to listen, the provision of advice, guidance, affirmation and encouragement) (Dang & Miller, 2013; Thompson et al., 2016; Van Dam et al., 2018) may be key to helping them move forward and integrate into the mainstream.

This intervention will provide 24 young people (ages 16 – 26) who have transitioned out of homelessness and into market rent housing within the past year with rent subsidies for 24 months. Half of the young people will also receive mentorship. We chose to focus on young people living in market rent housing because, due to limited subsidized housing options, this is the reality for most young people exiting homelessness.

Initially, we proposed to our community partners a study design where only half the young people would receive rent subsidies, with the other half receiving ‘treatment as usual’; however, we abandoned this idea after our community partners challenged the ethics of not providing or delaying rent subsidies for young people living a precarious existence and desperate for immediate, tangible support to help them remain in market rent housing. Consequently, we adopted the stance that, given housing is a basic human right[2], we would offer rent subsidies to *all* of the study participants. While this does move us away from the ‘gold standard’ in terms of measuring the impact of rent subsidies on social integration, we believe the mixed methods longitudinal design will still yield important insights in this regard. Moreover, this design adaptation reflects our deep commitment to engage in community-based participatory action research (CBPAR) – a

[2] “Adequate housing is essential to one’s sense of dignity, safety, inclusion and ability to contribute to the fabric of our neighbourhoods and societies…without appropriate housing it is often not possible to get and keep employment, to recover from mental illness or other disabilities, to integrate into the community, to escape physical or emotional violence or to keep custody of children” (Ontario Human Rights Commission, n.d.).

methodology that challenges traditional epistemological assumptions of what constitutes 'good' evidence, demands researcher humility, stresses genuine and equitable academic-community partnerships, and facilitates the undertaking of research in a way that the *community* feels is most beneficial to their members (Goodkind et al., 2017; Wallerstein & Duran, 2006; Wallerstein, Duran, Oetzel, & Minkler, 2018).

Half of the young people will be randomized to receive regular mentorship from an adult mentor, tasked with helping their mentee bridge the gap between homelessness and mainstream living. While some of these mentors will not be 'natural' in the sense that these may not be pre-existing, organically-formed relationships (see 3a. Mentorship), the mentors will incorporate the key relationship-based components of natural mentoring mentioned previously, with a strong emphasis on a strengths-based approach (i.e., focus on the young person's strengths as opposed to their limitations) and the connection of participants to larger social networks (including education and employment).

Findings from this longitudinal pilot randomized controlled trial will help address the gap in our knowledge about the impact of financial support and mentorship on meaningful social integration for young people who have experienced homelessness and are living in market rent housing.

2. Study Aim and Objectives

The overarching aim of this mixed methods study is to assess whether and how rent subsidies and mentorship influence social integration outcomes for formerly homeless young people living in market rent housing in three urban settings.

Specifically, the objectives of this study are to:

1. Determine whether rent subsidies plus mentorship results in better social integration outcomes than only receiving rent subsidies with respect to: a) community integration (psychological and physical); and b) self-esteem at our primary endpoint of 18 months.
2. Determine whether rent subsidies plus mentorship results in better social integration outcomes than only receiving rent subsidies with respect to: a) social connectedness; b) hope; and c) sustained academic and vocational participation at our secondary endpoint of 18 months.
3. Explore whether rent subsidies plus mentorship results in better social integration outcomes than only receiving rent subsidies with respect to: a) income; b) perceived housing quality; c) psychiatric symptoms; and d) sense of engulfment at our exploratory endpoint of 18 months.

4. Integrate qualitative data to facilitate a fuller understanding of the quantitative data and deepen our understanding of what the study participants (young people and mentors) found most beneficial about the intervention and how it could be improved.

3. Study Design

This study will employ a convergent mixed methods design (i.e., quantitative and qualitative data are collected concurrently, and the findings combined) embedded within a RCT and a CBPAR framework (Creswell, 2014; Creswell & Plano Clark, 2018). We believe a mixed methods RCT is appropriate given the complex explanatory pathways (i.e., social and behavioral processes that may act independently and interdependently) of this intervention (Lewin, Glenton, & Oxman, 2009). In addition, the qualitative data will provide insights on contextual factors that may impact the external validity of our findings (Goodkind et al., 2017). Most importantly, this design provides a crucial (and underutilized) youth-informed perspective on social integration.

The study will be conducted in three Canadian cities: Toronto, Ontario (pop. 2.8 million); Hamilton, Ontario (pop. 552,000); and St. Catharines, Ontario (pop. 133,000). The design and implementation of this study is very much collaborative effort between our research team and the following community partners: a) Covenant House Toronto; b) Living Rock Ministries; and c) The RAFT (St. Catharines).

All of the study participants (n = 24) will receive rent subsidies (ranging from \$400 – \$500/month)[3] for 24 months. This study includes funding for the rent subsidies and will be paid directly to the landlords by our community partners. St. Michael's Hospital will establish a service provider agreement with each of our community partners for this purpose.

3a. Mentorship

Participants in the intervention group (n = 12) will be matched with an adult mentor recruited by one of our community partners. Each of our community partners expressed a strong desire to take the lead in the screening and recruitment of mentors as they feel they are in the best position to work with the study participants to ensure the best mentor 'fit'. Drawing on the expertise of our community partners and sharing decision-making power is aligned with CBPAR principles and highlights our commitment to collaborative, equitable partnerships in all phases of the research process (Israel et al., 2018). Moreover, working with established community resources makes practical sense; not only will this facilitate co-learning and capacity

[3] Given the higher cost of rent in Toronto, youth living in Toronto will receive \$500/month, while youth living in Hamilton and St. Cathatrines will receive \$400/month.

building between the research team and our community partners (Israel et al.), but delivering the mentorship intervention under 'real world' conditions will provide important insights into scalability and sustainability (Wallerstein & Duran, 2010).

To build capacity between community partners, Covenant House Toronto will share their comprehensive *Mentor Program Guidelines* and *Mentor Orientation Handbook* (attached to this study protocol), which will act as a guide for all sites. These booklets cover information ranging from ideal mentor characteristics to mentor code of conduct. Each of our partners will designate one person currently serving in a leadership role within the organization to conduct one-on-one interviews with potential mentors and make the final decision (in conjunction with study participants) about mentor-mentee matches. In Hamilton and St. Catharines, this process will be undertaken by the executive director of each organization and, in Toronto, it will be done by the co-ordinator of their mentorship program.

As mentioned previously, our community partners are firm in their desire to control the mentorship screening and recruitment process, and will do so in a way that works best for each organization. That being said, all three organizations have agreed to the following preliminary screening process prior to meeting potential mentors:

- The mentor must show original documentation of passing a Vulnerable Sector police check within the past three months
- The mentor should ideally be at least five years older than the mentee
- The mentor must provide three references; one must be from a current employer

The mentors will be encouraged to incorporate the key relationship-based components of natural mentors previously described (e.g., a 'coach' or 'cheerleader' role) to assist with mainstream integration. To facilitate more of an organic, natural mentor-mentee relationship, the mentors will have more flexibility than a typical formal mentorship program in the types of activities they pursue with their mentees. For example, they will not be mandated to attend shelter-based social events. Instead, mentors will be encouraged to initiate activities that direct their mentees *away* from the shelter system (and their old identities as homeless youth) and toward the mainstream (e.g., meeting for coffee at a local university campus, touring a local library, or visiting the mentor's place of employment during business hours). All of the mentors will meet monthly with their mentees for two years. In addition, the mentor will be encouraged to touch base with their mentee via phone or text message every week. If a mentor is unable to continue their role and there are at least six months left in the study, the study participant will be matched with a new mentor.

3b. Outreach Worker

Our community partners will match all participants with an outreach worker (already employed by each agency and considered 'standard of care') who will communicate regularly with the research team, help ensure the rent subsidies are being distributed appropriately, maintain an ongoing relationship with the study participants, and monitor for 'red flags' in participants matched in mentor-mentee relationships (e.g., mentee reluctant to meet with their mentor). Matching all of the study participants with a worker will also help ensure that everyone is receiving a fairly equal level of social support from our community partners, making it easier for the research team to discern whether the outcomes of interest are more likely attributable to mentorship rather than to varied levels of agency-based support. Moreover, a review of services and interventions designed to reduce "problem behaviors" (p. 733) (e.g., substance use and risky sexual practices) among street-involved and homeless young people (ages 12 – 24) found that researchers who had strong relationships with outreach workers and the community had more effective interventions and lower attrition rates than those who did not (Slesnick, Dashora, Letcher, Erdem, & Serovich, 2009).

3c. Methodology

As previously noted, this mixed methods RCT is embedded within a CBPAR framework. With the goal of reducing health inequities through knowledge and action, CBPAR can be a powerful tool for those working with marginalized populations (Chenail, St. George, Wulff, & Cooper, 2012; Rutman, Hubberstey, Barlow, & Brown, 2005; Wallerstein & Duran, 2006; Wallerstein et al., 2018). The ontological and epistemological assumptions underpinning CBPAR methodology are closely aligned with Critical Social Theory – that is, the belief that social conditions (e.g., socioeconomic contexts) perpetuate societal power imbalances and shape our version of 'truth' (Denzin & Lincoln, 2011; Prasad, 2005). For example, some may underestimate the social integration challenges faced by formerly homeless young people because they believe that everyone is afforded the same life chances. Thus, researchers operating within this paradigm have a goal of exposing and critiquing the inequitable (and often invisible) conditions that make it challenging for the marginalized to move forward (Strega, 2005).

We will draw on the following key principles of CBPAR as we generate and analyse data (Chenail et al., 2012; Rutman et al., 2005; Wallerstein & Duran, 2006; Wallerstein et al., 2018):

- Research participants are viewed as experts in their own lives
- Concerted effort to reduce/eliminate power imbalances between the researchers and the community
- Equal value placed on academic (researcher) knowledge and experiential (community agency/youth) knowledge
- Commitment to producing practical, 'actionable' data to build community capacity and improve/transform the lives of the research participants

- Duty to remain invested with the community beyond the life of the research project

3d. Participant Eligibility and Recruitment

Twenty-four young people ages 16 – 26 who have left homelessness within the past year and are living in market rent housing will be collaboratively recruited by our research team and our community partners Covenant House Toronto, Living Rock Ministries, and The RAFT (St. Catharines). We will aim to have a roughly even balance of gender and ethno-racial representation at each site.

In addition to the above age and housing **inclusion** criteria, study participants must:

- Be able to provide free and informed consent
- Be fluent in English
- Plan on staying in or nearby the community in which they were recruited (Toronto, Hamilton, or St. Catharines) for the duration of the 24-month study
- Be willing to be matched with an adult mentor who has been screened and recommended by one of our three community partners (Covenant House Toronto, Living Rock Ministries, or The RAFT) **Note:** Each study participate will be able to select their own mentor once the potential mentors have been carefully screened by our community partners (see attached *Covenant House Toronto Mentor Program: Initial Screening Application; Covenant House Toronto Mentor Program: Screening Interview;* and *Covenant House Toronto Mentor Program: Confidential Volunteer Reference Form,* which our partners in Hamilton and St. Catharines will adopt as well*).*

Young people will be **excluded** from the study if they are:

- In imminent danger of losing their housing (e.g., facing jail time or impending eviction)
- Enrolled in another study with enhanced financial and social supports

Initial introduction to the study will be done by our community partners by someone within the youths' circle of care (e.g., an outreach worker or mental health counselor). This initial introduction will be done in-person (e.g., if the young person is visiting the agency) or over-the-phone. Agency staff will be instructed to utilize the *Transitioning Youth Out of Homelessness Information and Recruitment Poster* (see attached) and *Telephone Script for Contacting Potential Participants: Community Partners* (see attached) to guide their conversation. If a young person expresses interest in participating in the study, agency staff will obtain verbal consent to provide the young person's name, e-mail address and/or cell phone number to Dr. Naomi Thulien. Dr. Thulien will then forward this information on to the appropriate research team member (e.g., research assistant or research coordinator), who will connect with the youth over the phone (see attached *Telephone Script for Contacting Potential Participants: Research Team Member*).

3e. Consent Process

Free and informed consent will be obtained verbally and in writing from all study participants. A concerted effort has been made to ensure the consent form is in plain language. Highlighted throughout the document is the fact that informed consent is an ongoing process and can be negotiated at any time.

All of the study participants will be screened for eligibility (see criteria above) and recommended for the study by one of our community partners (Covenant House Toronto, Living Rock Ministries, and The RAFT). A member of the research team will call each recruited youth on the telephone and arrange to meet them at a location most convenient for the youth. Potential participants will be given a copy of the participant information and consent form to read. This document will also be reviewed verbally to ensure that those who have low literacy levels have been given the information required to give informed consent. During this process, the research team member will assess the capacity of the potential participant to provide free and informed consent. If it is unclear whether a youth is able to provide consent, the study co-investigator (Dr. Naomi Thulien) will be contacted immediately. Dr. Thulien will arrange for a qualified member of the research team to conduct a capacity assessment. If it is determined that a youth is not able to consent, they will be informed of this and they will be excluded from the study. The appropriate community partner will be notified as well.

3f. Allocation Procedure

If the participant meets the eligibility criteria, informed consent will be obtained, and the participant will be enrolled in the study. During this initial meeting, enrolled participants will participate in a baseline interview. There will be no unmasking of assignment prior to randomization. Following the baseline interview, participants at each of the three study sites (Toronto (n=12), Hamilton (n=6), and St. Catharine's (n=6)) will be randomized using block randomization to either the intervention (rent subsidies plus mentoring) or control (rent subsidies only) group. Randomization will be balanced by site based on random block sizes of two and four. The advantage of using block randomization is to uniformly distribute participants into treatment groups within each site (Efird, 2010). Because small block sizes may increase the risk of guessing the allocation procedure and subsequently introducing bias into the enrolment procedure, random block sizes will be used to avoid this potential selection bias (Suresh, 2011).

A unique randomization schedule will be produced for each site using SAS (SAS Institute Inc., Cary, NC, USA), with the algorithm described in Efird (2010) and will be generated by a statistician based at St. Michael's Hospital. A research coordinator based at St. Michael's Hospital and not affiliated with the study will be the only person with access to the randomization schedule. The research coordinator will prepare sealed, opaque and sequentially numbered envelopes with the randomization results of participants. After assessing for eligibility and obtaining consent of each participant, research personnel responsible for enrolling participants will open the next randomization envelope from the sequentially ordered randomization envelope file to obtain the participant's randomized group assignment. Randomization envelopes for all sites will be held in a locked cabinet at Centre for Urban Health Solutions, St. Michael's Hospital. The research coordinator enrolling participants will record the participant's group allocation into the Participant Linking Log, record their Participant ID number onto the randomization envelope, and return all opened randomization envelopes to the independent research coordinator at St. Michael's Hospital. Subsequently, the independent St. Michael's research coordinator will complete the Master Randomization Assignment List with Participant ID numbers corresponding to each envelope number to check for consistency in participant allocation. Both the Participant Linking Log and Master Randomization Assignment List will be securely kept on St. Michael's Hospital servers.

Participants will be informed immediately if they have been allocated to the intervention or control group (Figure 1). In keeping with typical community-based RCTs with psychosocial interventions, 'blinding' in this study would not be pragmatic (e.g., social service providers and mentors will know if participants are in the 'treatment' group) after the baseline interviews and random assignments have been conducted (Solomon, Cavanaugh, & Draine, 2009).

Figure 1.
CONSORT (**CON**solidation of the **S**tandards **O**f **R**eporting **T**rials) Diagram of Ideal Flow of Participants Through the Study.

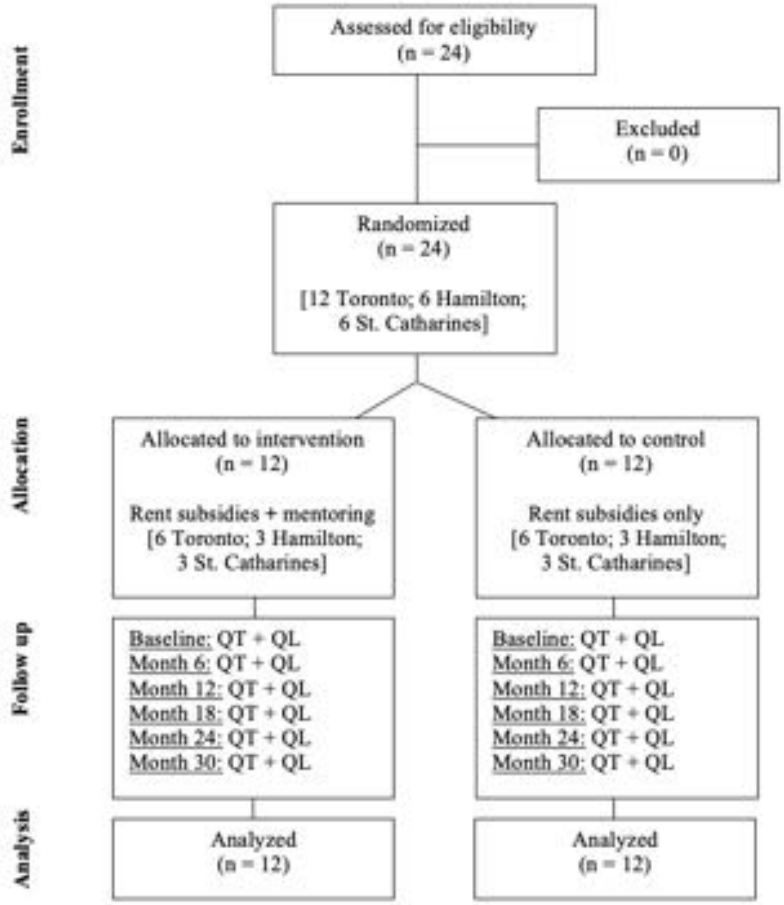

***QT** = quantitative measures (all participants). These will consist of six standardized measures to assess: community integration, social connectedness, engulfment, hope, self-esteem, and psychiatric symptoms. As well, participants will complete two brief questionnaires pertaining to: 1) education (includes skills training), employment, and income; and 2) perceived housing quality. **QL** = qualitative measures (12 participants). These will consist of one-on-one semi-structured interviews with the same 12 participants (six from each arm). The interview questions will explore issues related to feasibility and acceptability, and provide context to the quantitative responses.

4. Data Generation

Undertaking a mixed methods study where data is truly 'mixed' at the level of collection (not just at analysis) is challenging because it requires a solid understanding of the data generation requirements of each research paradigm, and the interviews can take longer to conduct than those focusing on qualitative or quantitative

methods alone (Farquhar, Ewing, & Booth, 2011). To minimize respondent burden, we have given careful consideration to the type of quantitative instruments chosen (e.g., number and length of time to complete) and will ensure that appropriate components of the qualitative interviews are prioritized (e.g., follow-up on changes in instrument scores) at each data generation session (Farquhar et al.). Additionally, all of the mixed method interviews will be conducted by the co-investigator, Dr. Naomi Thulien, who has expertise in conducting mixed method interviews with young people who have experienced homelessness.

4a. Study Outcomes

As mentioned previously, the mixed methods design of this pilot RCT reflects our desire to capture the complex independent and interdependent explanatory pathways of the intervention. This is especially crucial during this pilot stage, where we will be paying particular attention to feasibility, context, and unexpected mechanisms that produce change – factors that will influence study outcomes and provide important information regarding scalability and sustainability (Craig et al., 2008; Moore et al., 2015). To fully apprehend these complex explanatory pathways, “represent the best use of the data,” and “provide an adequate assessment of the success or otherwise of an intervention that has effects across a range of domains” (Craig et al., p.3), we have aligned our key outcome variables (Table 1) with the Medical Research Council guidance on evaluating complex interventions and identified more than one primary outcome measure.

Table 1. Key Outcome Variables

**Variables**	**Instruments***
Community integration (psychological and physical)<br><br>Social connectedness<br>Engulfment	Community Integration Scale<br><br><br>Social Connectedness Scale – Revised<br>Modified Engulfment Scale
Self-esteem<br><br>Hope<br>Psychiatric symptoms	Rosenberg Self-Esteem Scale<br><br>Beck Hopelessness Scale<br>Modified Colorado Symptom Index
Enrollment in education (includes skills training)<br>Employment	Composite checklist
Income	Composite checklist
Perceived housing quality	Perceived Housing Quality Scale

Participant perspectives of barriers and facilitators	Individual semi-structured interviews (youth) and focus groups (mentors)<br>Composite checklist (Mentor Evaluation)

*See Table 2 for references and psychometric properties. All will be administered every six months for 30 months (except for the mentor evaluation – see Table 2).

The **primary outcome measures** for this study are: community integration (psychological and physical) and self-esteem. **Secondary outcomes** include: social connectedness, hope, and academic and vocational participation. **Exploratory outcomes** include: engulfment, psychiatric symptoms, income, perceived housing quality, and participant perspectives of intervention barriers and facilitators.

4b. Study Hypothesis

We hypothesize that, for the **primary outcome** measures of community integration and self-esteem:

1. We will observe better mean scores (community integration and self-esteem) in the participants who receive rent subsidies plus mentorship (intervention group) compared to the participants who receive rent subsidies only (control group) by our **primary endpoint of 18 months** of study participation.

We hypothesise that, for the **secondary outcome** measures of social connectedness, hope, and academic and vocational participation:

1. We will observe better mean scores (social connectedness and hope) in the intervention group relative to participants in the control group by our **secondary endpoint of 18 months** of study participation.
2. Participants in the intervention group will be more likely than the control group to demonstrate sustained engagement in academic and vocational activities (education, employment, and/or skills training) by our **secondary endpoint of 18 months** of study participation.

4c. Quantitative Measures

Quantitative data (Appendix A – Quantitative Data Collection) will be collected at **six points** in time over the course of 30 months: baseline, month six, month 12, month 18, month 24, and month 30. **Nine instruments** (Table 2) will be employed to assess the outcome variables. We have purposely chosen instruments utilized in previous research with young people who have experienced homelessness (e.g., Kidd et al., 2016; Kozloff et al., 2016; McCay et al., 2015) so that meaningful comparisons can be made across studies (Moore et al., 2015) in this nascent area of research. One instrument we do not believe has been used with this population is the Modified Engulfment Scale (McCay & Seeman, 1998). We have included engulfment as an exploratory outcome given the emerging qualitative evidence on the crucial role of identity (self-concept) in a young

person's transition away from homelessness (Brueckner et al., 2011; Karabanow et al., 2016; Thulien et al., 2018).

Table 2. Quantitative Instruments

Instrument	Psychometric Information
Beck Hopelessness Scale (Beck, Weissman, Lester, & Trexler, 1974).	This 20-item scale measures motivation, expectations, and feelings about the future (internal consistency α = .93).
Community Integration Scale (Stergiopoulos et al., 2014).	This 11-item scale measures behavioral (e.g., participation in activities) and psychological (e.g., sense of belonging) aspects of community integration. This scale was used extensively in the Chez Soi/At Home study, but psychometric properties have yet to be reported.
Education, Employment, and Income Questionnaire	This 13-item questionnaire assesses education, employment, and income. We developed this questionnaire for the study.
Mentor Evaluation Questionnaire	This 10-item questionnaire assesses mentor effectiveness. It will be completed at month 24 by those in the intervention group. We developed this questionnaire for the study in collaboration with our community partners.
Modified Colorado Symptom Index (Ciarolo et al., 1981).	This 14-item scale measures the presence and frequency of psychiatric symptoms experienced in the past month. (internal consistency α = .90 – .92).
Modified Engulfment Scale (McCay & Seeman, 1998).	This 30-item scale measures the degree to which an individual's self-concept is defined by their experience of homelessness (internal consistency α = .91). We have adapted the scale for this study, substituting "experience of homelessness" for "illness".
Perceived Housing Quality (Toro et al., 1997).	This seven-item scale measures participant perception of housing choice and quality. This scale was used extensively in the Chez Soi/At Home study, but psychometric properties have yet to be reported. We have shortened it from 10 items (Chez Soi/At Home) to seven relevant items.
Rosenberg Self-Esteem Scale (Rosenberg, 1965).	This 10-item scale measures global self-worth (internal consistency α = .77 – .88).
Social Connectedness Scale	This 20-item scale measures belongingness – the degree to which

– Revised (Lee & Robbins, 1995).	an individual feels connected to others (internal consistency α = .92).

4d. Qualitative Measures

Qualitative measures (see Appendix B – Qualitative Data Generation) are an important feature of this study and will consist of: **1) semi-structured individual interviews** (study participants) and **2) focus groups** (mentors).

At baseline, twelve participants (six from each arm of the study) will be invited to participate in **six semi-structured individual interviews**, which will take place at the same time as the quantitative data collection: **baseline, month six, month 12, month 18, month 24, and month 30**. Participants will be purposively selected with a goal of having input from each of the three communities and a fairly equal gender and ethno-racial representation.

All of the mentors (n = 12) will be invited to participate in **two focus groups**, which will take place at **month 12 and month 24**.

The questions posed during the semi-structured interviews and focus groups will be guided by the study objectives, but will be conversational and exploratory in nature with particular attention to understanding *how* mentoring and/or rent subsidies influence social integration outcomes for formerly homeless young people living in market rent housing. Given the emergent nature of qualitative inquiry (Denzin & Lincoln, 2011; Eakin & Mykhalovskiy, 2003), we expect the interview and focus group questions to evolve over time as key preliminary themes begin to surface. It is anticipated that the individual interviews (including quantitative data collection, which will consistently take place first) will last approximately 60 – 75 minutes, and the focus groups approximately 60 – 90 minutes. The individual interviews and the focus groups will be conducted by Dr. Naomi Thulien at locations most convenient for those participating. To get a better sense of each young person's living situation and to minimize researcher – participant power imbalance (Israel et al., 2018), Dr. Thulien will suggest that the individual interviews take place in or nearby the young people's homes. The individual interviews and focus groups will be audio recorded and transcribed verbatim.

4e. Honoraria

All of the study participants will be paid an honorarium of $20 at each of the six quantitative data collection points. Those participating in semi-structured interviews will be paid an additional $30 at each interview. This

amount was based on the co-investigator's previous experience with this population and after consulting with our community partners.

5. Data Analysis

One major critique of mixed methods RCTs is that, typically, there is no true integration (i.e., 'mixing') of quantitative and qualitative findings at the level of analysis or interpretation (Lewin, Glenton, & Oxman, 2009). Moreover, it is often unclear whether or how the quantitative and qualitative researchers have worked together to maximize the potential synergies between these different approaches (Lewin et al.). With this in mind, our study team, consisting of researchers with quantitative and qualitative expertise, worked together to develop this study protocol and anticipate meeting quarterly to discuss the emerging analysis and to explore (and follow up on) similarities or discrepancies between the quantitative and qualitative data.

5a. Quantitative Data

All analyses will be performed using the intention-to-treat principle; that is, all participants will be included and analyzed in the groups they were originally randomized. Baseline characteristics of the intervention and control groups will be summarized using descriptive statistics (i.e., mean, standard deviation, median and interquartile range for continuous variables, and frequencies and proportions for categorical variables). We will also calculate descriptive statistics for outcomes at each study time point, and will explore differences in trajectories from baseline to 30 months follow-up between intervention and control groups using scatterplots and box-plots. Differences with 95% confidence intervals in continuous outcomes at 18 months (psychological community integration, self-esteem, social connectedness, hope, perceived housing quality, psychiatric symptoms, and sense of engulfment) between participants who received rent subsidies plus mentorship and participants who only received rent subsidies will be estimated using Analysis of Covariance (i.e., linear regression models), including an indicator of intervention group and the baseline value of the outcome. We will perform regression diagnostics and will repeat analyses using the non-parametric Wilcoxon rank-sum test if there are extreme outliers or influential observations. Groups will be compared with respect to count outcomes at 18 months (physical community integration) using graphical tools and the non-parametric Wilcoxon rank-sum test. For binary outcomes at 18 months (sustained academic and vocational participation, and income above low income cut-off[4]), differences in proportions with 95% confidence intervals will be estimated and tested using the chi-square or Fisher's exact test. Given the small sample size of this pilot randomized trial, all results will be interpreted with caution and with the intention of generating

---

[4] Based on family and community size.

data and hypotheses for conducting a larger trial.

All efforts will be made to reduce participants' attrition and drop-out. As mentioned previously, we believe our strong relationship with the outreach workers and community agencies will help minimize loss to follow-up (Slesnick et al., 2009). In addition, we have made it very clear in the participant information and consent form that participants in the rent subsidies plus mentorship arm may continue in the study (receiving only rent subsidies) if they are unable to continue in a mentor-mentee relationship.

5b. Qualitative Data

In keeping with the emergent, iterative nature of research using a qualitative design (Denzin & Lincoln, 2011; Eakin & Mykhalovskiy, 2003), data analysis and interpretation will begin immediately after the first qualitative data generation session (at baseline). The semi-structured individual interviews and focus groups will be audio recorded and transcribed verbatim. In order to conduct a more nuanced analysis of the data, the transcriptionist will be instructed to note short responses, uncooperative tones, and literal silence (Eakin & Mykhalovskiy; Kawabata & Gastaldo, 2015). Prior to each subsequent qualitative data generation session, members of the research team will conduct a preliminary data analysis, reading the interview transcripts multiple times, separating the data into coded segments, making analytic memos beside sections of the transcripts, identifying emerging themes (and comparing/contrasting these between respondents), and compiling new questions (Creswell, 2014; Denzin & Lincoln). Those participating in the individual interviews and the focus groups will be asked for their perspectives on the emerging interpretations at each visit and these perspectives will play a key role in helping shape the data analysis and help ensure the trustworthiness of the data (Creswell; Loiselle, Profetto-McGrath, Polit, & Tatano Beck, 2004). The web-based application Dedoose (SocioCultural Research Consultants, LLC, 2018) will be utilized to assist with sorting and coding the qualitative data.

6. Ethical Considerations

There are important ethical considerations that must be considered with any type of research. This is especially true of RCTs conducted with marginalized populations (Solomon et al., 2009). Accordingly, we have endeavored to weave ethical considerations into all aspects of the study design (Solomon et al.), including our decision to utilize a CBPAR methodology and to modify the study design so that all of the participants will receive rent subsidies. Ethical approval for this study will be obtained from the Providence St. Joseph's and St. Michael's Healthcare Research Ethics Board (REB).

6a. Benefits and Risks to Participants

All of the study participants will likely find it beneficial to receive rent subsidies. Those randomized to the intervention group may also benefit from receiving regular interactions with a mentor. Participants selected for qualitative interviews might benefit from the opportunity to share their integration-related experiences with Dr. Thulien on a regular basis. Additionally, participants may derive satisfaction from knowing that their contributions will help advance our understanding about how best to design interventions that assist formerly homeless young people to achieve meaningful social integration.

We believe theoretical justification exists for expecting that the proposed mentoring intervention is likely to produce effective outcomes; however, research ethics demands that we mitigate any potential risk to the research participants (Solomon et al., 2009). As previously mentioned, we will rely on the expertise of our community partners to screen and train the mentors. In addition, as highlighted previously, the outreach workers will work closely with the study participants and with our research team, and will alert our team if there are any concerns about a mentor-mentee relationship. These concerns will be relayed to our community partners so they can take appropriate action. The Providence St. Joseph's and St. Michael's Healthcare REB will be notified if a mentor-mentee relationship is terminated due to actions that violate the mentor-mentee code of conduct.

Participants will be assured that their participation or lack of participation in the study will not negatively impact their relationship with our community partners or their ability to access services at St. Michael's Hospital or support from other social services agencies (e.g., OW/ODSP).

6b. Privacy and Confidentiality

This mixed methods RCT will use multiple and varied data sources. This comprehensiveness is critical to the objectives of the study, yet may increase invasion of participant privacy. This privacy concern will be clearly communicated to potential participants, as will the measures for protecting security and confidentiality, prior to consent.

All of the data collected will be kept in strict confidence. While participants' names will appear on the consent forms, pseudonyms (created by the participants) will be used in place of their real names on all documents related to data generation, including the audio recordings and interview transcripts. A key that links each participant name with a pseudonym will be created and stored as a separate electronic file. All electronic data will be stored on secure servers at the Centre for Urban Health Solutions or the McMaster University School of Nursing, and only be accessible by select members of the research team.

The individual interviews and focus groups will be audio recorded using a password protected application on a password protected electronic device. The audio recordings from the individual interviews and focus groups will be deleted once the transcripts have been stored on the secure server and entered into Dedoose (encrypted and password-protected) (SocioCultural Research Consultants, LLC, 2018). Paper copies of the data (e.g., consent forms and standardized quantitative measures) will be stored in a locked filing cabinet at the Centre for Urban Health Solutions or at the McMaster University School of Nursing – areas only accessible to those with electronic and key access. All paper and electronic files will be retained for a period of up to five years from study closure.

The consent form indicates that limits to confidentiality apply if a participant discloses that they intended to hurt themselves or others, or if they inform a member of the research team that someone under the age of 16 years is suffering abuse and/or neglect.

7. Dissemination

In keeping with our CBPAR methodology, we are committed to disseminating evidence *with* our community partners to build community capacity and improve the lives of the young people participating in this study (Chenail et al., 2012; Wallerstein et al., 2018). Moreover, given our use of Critical Social Theory, we are obliged to not only present our findings, but to expose and explicate the relational processes (e.g., subjective experience of low socioeconomic position and low social class) that may be preventing formerly homeless young people from achieving meaningful social integration (Madison, 2012; Strega, 2005). With an emphasis on 'actionable' data (Chenail et al.), we anticipate disseminating our findings broadly to both academic and community-based audiences in a variety of formats ranging from scientific journal papers to oral presentations.

7a. Documentary Film

Three study participants – one from each city – will be invited to participate in a documentary film. The primary purpose of the film is two-fold: 1) to highlight the impact of rent subsidies and mentorship on meaningful socioeconomic integration and 2) to advocate for enhanced economic and social supports for young people transitioning out of homelessness. We believe this form of dissemination is very much in keeping with our commitment to CBPAR methodology and Critical Social Theory scaffolding.

Participant recruitment and consent will take place as follows:

- Dr. Thulien will reach out to three young people involved in qualitative interviews (all being conducted by Dr. Thulien) that she believes would be good candidates for the film

- Dr. Thulien will provide a high-level overview of the film and review the Participant Information and Consent: Documentary Film and St. Michael's Hospital Audio-Visual Consent for Non-Clinical Use forms with participants (will be e-mailed to participants prior to conversation)
- If participants are interested in learning more, Dr. Thulien will connect them with film director/producer Catie Lamer in a three-way Zoom or telephone call (Dr. Thulien, Catie Lamer, and the interested study participant – separate conversations with each potential film participant)
- During the three-way conversation, young people will be encouraged to ask questions about filming/screening
- After the three-way conversation, young people will be given 48 hours to consider participating (longer if needed) – after this period, Dr. Thulien will reach out to them again
- Participants interested in taking part in the film will sign the Participant Information and Consent: Documentary Film and St. Michael's Hospital Audio-Visual Consent for Non-Clinical Use after reviewing them (again) with Dr. Thulien
- After consent is received, Dr. Thulien will inform Catie Lamer, who will reach out to the participants and begin the process of filming

8. Limitations

This study has a number of limitations. First, the young people recruited for this pilot study will be a small sample of youth connected to urban-based social service providers in the province of Ontario. Thus, the findings may not be generalizable to formerly homeless young people living in other contexts and/or not connected to social service agencies. Second, the quantitative instruments are based on self-report and may involve a degree of response bias. Finally, the quantitative measures we have chosen are what we believe to be surrogate markers of meaningful social integration. Future work will likely be needed to more accurately capture this complex concept.

9. Significance

This pilot RCT study will be the first to test the impact of economic and social supports on meaningful social integration for formerly homeless young people living in market rent housing. We believe the mixed methods design will illuminate important contextual factors that must be considered if the intervention is to be scaled up and replicated elsewhere. Importantly, the CBPAR framework will incorporate the perspectives of the community, including formerly homeless young people, who are in the best position to determine what might work best in the context of their lives.

References

Altena, A. M., Brilleslijper-Kater, S. N., & Wolf, J. R. (2010). Effective interventions for homeless youth: A systematic review. *American Journal of Preventive Medicine*, *38*(6), 637-645. doi: 10.1016/j.amepre.2010.02.017.

Beck, A. T., Weissman, A., Lester, D., & Trexler, L. (1974). The measurement of pessimism: The hopelessness scale. *Journal of Consulting and Clinical Psychology, 41*(6), 639–660. doi: 10.1037/h0037562.

Brueckner, M., Green, M., & Saggers, S. (2011). The trappings of home: Young homeless people's transitions towards independent living. *Housing Studies*, *26*(1), 1-16. doi:10.1080/02673037.2010.512751.

Chenail. R. J., St. Goerge, S., Wulff, D., & Cooper, R. (2012). Action research: The methodologies. In P. L. Munhall (Ed.), *Nursing research: A qualitative perspective* (5th ed.) (pp. 455-470). Sudbury, MS: Jones & Bartlett.

Ciarolo, J. A., Edwards, D. W., Kiresuk, T. J., Newman, F. L., & Brown, T. R. (1981). *Colorado symptom index.* Washington, DC: National Institute of Mental Health.

Coren, E., Hossain, R., Pardo Pardo, J., & Bakker, B. (2016). Interventions for promoting reintegration and reducing harmful behaviour and lifestyles in street-connected children and young people. *Cochrane database of systematic reviews*, *2016*(1), 1-152. doi: 10.1002/14651858.CD009823.pub3.

Craig, P., Dieppe, P., Macintyre, S., Michie, S., Nazareth, I., & Petticrew, M. (2008). Developing and evaluating complex interventions: The new Medical Research Council guidance. *BMJ*, *337*(a1655), 1-6. doi: 10.1136/bmj.a1655.

Creswell, J.W. (2014). *Research design: Qualitative, quantitative, and mixed method approaches* (4th ed.). Thousand Oaks, CA: Sage.

Creswell, J.W., & Plano Clark, V.L. (2018). Designing and conducting mixed methods research (3rd ed.). Thousand Oaks, CA: Sage.

Dang, M. T., & Miller, E. (2013). Characteristics of natural mentoring relationships from the perspectives of homeless youth. *Journal of Child and Adolescent Psychiatric Nursing*, *26*(4), 246-253. doi: 10.1111/jcap.12038.

Denzin, N.K., & Lincoln, Y.S. (2011). *The SAGE handbook of qualitative research*. Thousand Oaks, CA: SAGE Publications Inc.

Eakin, J.M., & Mykhalovskiy, E. (2003). Reframing the evaluation of qualitative health research: Reflections on a review of appraisal guidelines in the health sciences. *Journal of Evaluation in Clinical Practice*, *9*(2), 187-194. doi: 10.1046/j.1365-2753.2003.00392.x.

Efird, J. (2011). Block randomization with randomly selected block sizes. *International Journal of Environmental Research and Public Health, 8*(1), 15-20. doi: 10.3390/ijerph8010015.

Farquhar, M. C., Ewing, G., & Booth, S. (2011). Using mixed methods to develop and evaluate complex interventions in palliative care research. *Palliative Medicine*, *25*(8), 748-757. doi: 10.1177/0269216311417919.

Fletcher, R.H., Fletcher, S.W., & Fletcher, G.S. (2014). *Clinical epidemiology: The essentials* (5th ed.). Philadelphia, PA: Lippincott Williams & Wilkins.

Frederick, T., Chwalek, M., Hughes, J., Karabanow, J., & Kidd, S. (2014). How stable is stable? Defining and measuring housing stability. *Journal of Community Psychology, 42*(8), 964- 979. doi:10.1002/jcop.21665.

Gaetz, S. (2014). *Coming of age: Reimagining the response to youth homelessness in Canada.* Toronto, ON: The Canadian Homelessness Research Network Press. Retrieved from http://www.homelesshub.ca/comingofage

Gaetz, S., Dej, E., Richter, T., & Redman, M. (2016) *The state of homelessness in Canada 2016.* Toronto, ON: Canadian Observatory on Homelessness Press. Retrieved from http://www.homelesshub.ca/SOHC2016

Gaetz, S., O'Grady, B., Kidd, S., & Schwan, K. (2016). *Without a home: The national youth homelessness survey.* Toronto: Canadian Observatory on Homelessness Press. Retrieved from http://homelesshub.ca/sites/default/files/WithoutAHome-final.pdf

Gaetz, S., & Redman, M. (2016). *Federal investment in youth homelessness: Comparing Canada and the United States and a proposal for reinvestment. Canadian observatory on homelessness policy brief.* Toronto, ON: The Homeless Hub Press. Retrieved from http://homelesshub.ca/sites/default/files/Policy_Brief.pdf

Goodkind, J. R., Amer, S., Christian, C., Hess, J. M., Bybee, D., Isakson, B. L., ... Shantzek, C. (2017). Challenges and innovations in a community-based participatory randomized controlled trial. *Health Education & Behavior*, *44*(1), 123-130. doi: 10.1177/1090198116639243

Hammersley, M., & Atkinson, P. (2007). *Ethnography: Principles in Practice* (3rd ed.). London, UK: Routledge.

Hwang, S.W., & Burns, T. (2014). Health interventions for people who are homeless. *The Lancet, 384*(9953), 1541-1547. doi: 10.1016/S0140-6736(14)61133-8.

Israel, B.A., Schulz, A.J., Parker, E.A., Becker, A.B., Allen, III, Guzman, R.J., & Lichtenstein, R. (2018). Critical issues in developing and following CBPR principles. In N. Wallerstein, B. Duran, J. Oetzel, & M. Minkler (Eds.), *Community-based participatory research for health: Advancing social and health equity* (3rd ed.) (pp. 31-44). San Francisco, CA: Jossey-Bass.

Karabanow, J. (2008). Getting off the street: Exploring the process of young people's street exits. *American Behavioral Scientist, 51*(6)*,* 772-788. doi:10.1177/0002764207311987. Karabanow, J., Carson, A., & Clement, P. (2010). *Leaving the streets: Stories of Canadian youth*. Halifax, NS: Fernwood Publishing.

Karabanow, J., Kidd, S., Frederick, T., & Hughes, J. (2016). Toward housing stability: Exiting homelessness as an emerging adult. *Journal of Sociology & Social Welfare, 43*(1), 121- 148. Retrieved from https://wmich.edu/socialworkjournal

Kawabata, M., & Gastaldo, D. (2015). The less said, the better: Interpreting silence in qualitative research. *International Journal of Qualitative Research Methods, 14*(4), 1-9. doi:10.1177/1609406915618123.

Kidd, S.A., Frederick, T., Karabanow, J., Hughes, J., Naylor, T., & Barbic, S. (2016). A mixed methods study of recently homeless youth efforts to sustain housing and stability. *Child and Adolescent Social Work Journal*, *33*(3), 207-218. doi:10.1007/s10560-015-0424.

Kozloff, N., Adair, C. E., Lazgare, L. I. P., Poremski, D., Cheung, A. H., Sandu, R., & Stergiopoulos, V. (2016). "Housing first" for homeless youth with mental illness. *Pediatrics*, *138*(4), e20161514. doi: 10.1542/peds.2016-1514

Kulik, D.M., Gaetz, S., Crowe, C., & Ford-Jones, E.L. (2011). Homeless youth's overwhelming health burden: A review of the literature. *Paediatric Child Health*, *16*(6), e43-e47. doi: 10.1093/pch/16.6e43.

Kusenbach, M. (2003). Street phenomenology: The go-along as ethnographic research tool. *Ethnography, 4*(3), 455-485. doi: 10.1177/146613810343007.

Lee, R. M., & Robbins S. B. (1995). Measuring belongingness: The social connectedness and the social assurance scales. *Journal of Counseling Psychology*, *42*(2), 232-241. doi: 10.1037/0022-0167.42.2.232.

Lewin, S., Glenton, C., & Oxman, A. D. (2009). Use of qualitative methods alongside randomised controlled trials of complex healthcare interventions: methodological study. *BMJ*, *339*(b3496), 1-7. doi: 10.1136/bmj.b3496.

Loiselle, C.G., Profetto-McGrath, J., Polit, D.F., & Tatano Beck, C.T. (2004). *Canadian essentials of nursing research*. Philadelphia, PA: Lippincott Williams & Wilkins.

Luchenski, S., Maguire, N., Aldridge, R. W., Hayward, A., Story, A., Perri, P., ... Hewett, N. (2017). What works in inclusion health: overview of effective interventions for marginalised and excluded populations. *The Lancet*, *391*(10117), 266-280. doi:10.1016/ S0140-6736(17)31959-1.

Madison, D.S. (2012). *Critical ethnography: Method, ethics, and performance* (2nd ed.). Thousand Oaks, CA: Sage Publication, Inc.

Mayock, P., O'Sullivan, E., & Corr, M.L. (2011). Young people exiting homelessness: An exploration of process, meaning and definition. *Housing Studies, 26*(6), 803-826. doi:10.1080/02673037.2011.593131.

McCay, E., Carter, C., Aiello, A., Quesnel, S., Langley, J., Hwang, S., ... Karabanow, J. (2015). Dialectical Behavior Therapy as a catalyst for change in street-involved youth: A mixed methods study. *Children and Youth Services Review*, *58*, 187-199. doi: 10.1016/j.childyouth.2015.09.021.

McCay, E. & Seeman, M. (1998). A scale to measure the impact of a schizophrenic illness on an individual's self-concept. *Archives of Psychiatric Nursing, 12(1),* 41-49. doi: 10.1016/S0883-9417(98)80007-1.

Milburn, N.G., Rice, E., Rotheram-Borus, M.J., Mallett, S., Rosenthal, D., Batterham, P., ... Duan, N. (2009). Adolescents exiting homelessness over two years: The risk amplification and abatement model. *Journal of Research on Adolescence, 19*(4), 762- 785. doi:10.1111/j.1532-7795.2009.00610.x.

Moore, G. F., Audrey, S., Barker, M., Bond, L., Bonell, C., Hardeman, W., ... Baird, J. (2015). Process evaluation of complex interventions: Medical Research Council guidance. *BMJ, 350*(h1258), 1-6. doi: 10.1136/bmj.h1258.

Ontario Human Rights Commission. (n.d.). *Housing as a human right.* Retrieved from http://www.ohrc.on.ca/en/right-home-report-consultation-human-rights-and-rental-housing-ontario/housing-human-right

Popay, J., Escorel, S., Hernandez, M., Johnston, H., Mathieson, J., & Rispel, L. (2008). *Understanding and tackling social exclusion: Final report to the WHO commission on social determinants of health from the social exclusion knowledge network*. Retrieved from http://www.who.int/social_determinants/themes/socialexclusion/en/

Prasad, P. (2005). *Crafting qualitative research: Working in the postpositivist traditions.* New York, NY: M.E. Sharpe.

Public Interest. (2009). *Changing patterns for street involved youth.* Toronto, ON: Author. Retrieved from http://www.worldvision.ca/Programs-and- Projects/Canadian Programs/Documents/ChangingPatternsForStreetInvolvedYouth.pdf

Quilgars, D., & Pleace, N. (2016). Housing First and Social Integration: A Realistic Aim? *Social Inclusion*, *4*(4), 5-15. doi:10.17645/si.v4i4.672.

Rosenberg, M. (1965). Society and the adolescent self-image. Princeton, NJ: Princeton University Press.

Rutman, A., Hubberstey, A., Barlow, A., & Brown, E. (2005). Supporting young people's

transitions from care: Reflections on doing participatory action research with youth from care. In L. Brown & S. Strega (Eds.), *Research as resistance: Critical, Indigenous, & anti-oppressive approaches* (pp. 153-179). Toronto, ON: Canadian Scholars' Press/Women's Press.

Slesnick, N., Dashora, P., Letcher, A., Erdem, G., & Serovich, J. (2009). A review of services and interventions for runaway and homeless youth: Moving forward. *Children and Youth Services Review*, *31*(7), 732-742. doi: 10.1016/j.childyouth.2009.01.006.

SocioCultural Research Consultants, LLC. (2018). Dedoose (Version 8.0.35) [web application]. Retrieved from http://www.dedoose.com

Solar, O., & Irwin, A. (2010). *A conceptual framework for action on the social determinants of health: Social determinants of health discussion paper 2.* Geneva, Switzerland: World Health Organization Press. Retrieved from http://www.who.int/sdhconference/resources/ConceptualframeworkforactiononS DH_eng.pdf

Solomon, P., Cavanaugh, M.M., & Draine, J. (2009). *Randomized controlled trials: Design and implementation for community-based psychosocial interventions.* New York, NY: Oxford University Press.

Stergiopoulos, V., Gozdzik, A., O'Campo, P., Holtby, A., Jeyaratnam, J., & Tsemberis, S. (2014). Housing first: Exploring participants' early support needs. *BMC Health Services Research, 14*(167), 1-15. doi:10.1186/1472-6963-14-16.

Strega, B. (2005). The view from the poststructural margins: Epistemology and methodology reconsidered. In L. Brown & S. Strega (Eds.), *Research as resistance: Critical, Indigenous, & anti-oppressive approaches* (pp. 199-235). Toronto, ON: Canadian Scholars' Press/Women's Press.

Suresh, K. P. (2011). An overview of randomization techniques: an unbiased assessment of outcome in clinical research. *Journal of human reproductive sciences*, *4*(1), 8-11. doi: 10.4103/0974-1208.82352.

Thompson, A. E., Greeson, J. K., & Brunsink, A. M. (2016). Natural mentoring among older youth in and aging out of foster care: A systematic review. *Children and Youth Services Review*, *61*, 40-50. doi: 10.1016/j.childyouth.2015.12.006.

Thulien, N. S., Gastaldo, D., Hwang, S. W., & McCay, E. (2018). The elusive goal of social integration: A critical examination of the socio-economic and psychosocial consequences experienced by homeless young people who obtain housing. *Canadian Journal of Public Health*, *109*(1), 89-98. doi: 10.17269/s41997-018-0029-6.

Toro, P. A., Rabideau, J. M. P., Bellavia, C. W., Daeschler, C. V., Wall, D. D., Thomas, D.

M., & Smith, S. J. (1997). Evaluating an intervention for homeless persons: results of a field experiment. *Journal of consulting and clinical psychology*, *65*(3), 476-484. doi: 10.1037//0022-006X.65.3.476.

Van Dam, L., Smit, D., Wildschut, B., Branje, S. J. T., Rhodes, J. E., Assink, M., & Stams, G. J. J. M. (2018). Does natural mentoring matter? A multilevel meta-analysis on the association between natural mentoring and youth outcomes. *American Journal of Community Psychology, 0,* 1-18. doi: 10.1002/ajcp.12248.

Wallerstein, N. & Duran, B. (2006). Using community-based participatory research to address health disparities. *Health Promotion Practice, 7*(3), 312-323. doi: 10.1177/1524839906289376.

Wallerstein, N. & Duran, B. (2010). Community-based participatory research contributions to intervention research: The intersection of science and practice to improve health equity. *American Journal of Public Health, 100*(S1), S40-S46. doi: 10.2105/AJPH.2009.

Wallerstein, N., Duran, B., Oetzel, J.G., & Minkler, M. (2018). *Community-based participatory research for health: Advancing social and health equity* (3rd ed.). San Francisco, CA: Jossey-Bass.
