# Supplementary material for: Effect of Portable Rent Subsidies and Mentorship on Socioeconomic Inclusion for Young People Exiting Homelessness: A Community-Based Pilot Randomized Clinical Trial
Source: JAMA Netw Open. 2022 Oct 27;5(10):e2238670. doi: 10.1001/jamanetworkopen.2022.38670 (PMC9614573; doi:10.1001/jamanetworkopen.2022.38670)
Supplement: Supplement 2. — Data Sharing Statement [file jamanetwopen-e2238670-s002.pdf]

Data Sharing Statement

Thulien. Effect of Portable Rent Subsidies and Mentorship on Socioeconomic Inclusion for Young People Exiting Homelessness. *JAMA Netw Open*. Published October 27, 2022. doi:10.1001/jamanetworkopen.2022.38670

Data
Data available: Yes
Data types: Deidentified participant data
How to access data: Requests can be made to Dr. Naomi Thulien (Naomi.thulien@unityhealth.to).
When available: With publication

Supporting Documents
Document types: None

Additional Information
Who can access the data: Data will be made available to researchers whose proposed use of the data has been approved.
Types of analyses: Secondary quantitative analysis.
Mechanisms of data availability: With a signed data access agreement.
